# Vanadium-Doped FeBP Microsphere Croissant for Significantly Enhanced Bi-Functional HER and OER Electrocatalyst

**DOI:** 10.3390/nano12193283

**Published:** 2022-09-21

**Authors:** Shalmali Burse, Rakesh Kulkarni, Rutuja Mandavkar, Md Ahasan Habib, Shusen Lin, Young-Uk Chung, Jae-Hun Jeong, Jihoon Lee

**Affiliations:** Department of Electronic Engineering, College of Electronics and Information, Kwangwoon University, Nowon-gu, Seoul 01897, Korea

**Keywords:** water splitting, V-FeBP, heteroatom doping, hydrothermal approach, soaking approach

## Abstract

Ultra-fine hydrogen produced by electrochemical water splitting without carbon emission is a high-density energy carrier, which could gradually substitute the usage of traditional fossil fuels. The development of high-performance electrocatalysts at affordable costs is one of the major research priorities in order to achieve the large-scale implementation of a green hydrogen supply chain. In this work, the development of a vanadium-doped FeBP (V-FeBP) microsphere croissant (MSC) electrocatalyst is demonstrated to exhibit efficient bi-functional water splitting for the first time. The FeBP MSC electrode is synthesized by a hydrothermal approach along with the systematic control of growth parameters such as precursor concentration, reaction duration, reaction temperature and post-annealing, etc. Then, the heteroatom doping of vanadium is performed on the best FeBP MSC by a simple soaking approach. The best optimized V-FeBP MSC demonstrates the low HER and OER overpotentials of 52 and 180 mV at 50 mA/cm^2^ in 1 M KOH in a three-electrode system. In addition, the two-electrode system, i.e., V-FeBP || V-FeBP, demonstrates a comparable water-splitting performance to the benchmark electrodes of Pt/C || RuO_2_ in 1 M KOH. Similarly, exceptional performance is also observed in natural sea water. The 3D MSC flower-like structure provides a very high surface area that favors rapid mass/electron-transport pathways, which improves the electrocatalytic activity. Further, the V-FeBP electrode is examined in different pH solutions and in terms of its stability under industrial operational conditions at 60 °C in 6 M KOH, and it shows excellent stability.

## 1. Introduction

Hydrogen is an efficient green-energy resource with its high gravimetric energy density and carbon-free nature. Hydrogen has emerged as a promising substitution for fossil fuels, which can then gradually decrease climate change and global warming [1,2,3,4,5]. Hydrogen also offers excellent transportability and is convenient to store in a compressed gas and liquid form, much like natural gas and oil. Currently, noble-metal-based electrocatalysts such as Pt/Pd and RuO_2_/IrO_2_ are the benchmark electrodes for water splitting. However, the practical production of ultra-fine hydrogen by water electrolysis is hindered due to the limited availability of these elements and high costs [6,7,8]. The development of highly active electrocatalysts at an affordable cost remains to be one of the major research priorities for the green hydrogen supply chain.

Over the last decade, transition metals (TMs) such as Co, Ni, Cu, Fe, W, Mo, Mn, V, etc., combined with several non-metallic elements including phosphorus, selenium, carbon, sulfur, nitrogen, etc., have been widely researched as efficient water-splitting catalysts [9,10,11,12,13,14,15]. The TMs possess fewer filled d-orbitals and their combination with non-metallic elements can offer superior intrinsic water-splitting capabilities and significantly enhance HER and OER kinetics [16,17]. Among them, iron (Fe) is one of the most earth-abundant elements and can offer good stability; thus, the iron-based electrodes can be a cost-effective alternative for the practical industrialization of water splitting. Fe-based compounds and structures demonstrated effective adsorption/desorption of reaction intermediates in the overall water-splitting process [9]. For example, the ultra-thin FeP nanosheets exhibited an excellent electrocatalytic oxygen-evolution performance with the Fe defects on the FeP nanosheets, which facilitated the adsorption of oxygenated intermediates and a low overpotential [18]. The Fe_2_O_3_/FeP heterostructure demonstrated excellent OER with the reduced reaction barrier due to the large surface area and lower charge-transfer resistance [5].

Meanwhile, phosphorus (P) is one of the most widely studied non-metallic elements that has been frequently compounded with TMs in various combinations, and remarkable advances have been made up to now [19,20,21,22]. More recently, boron (B) has emerged as another potential non-metallic element that can be combined with TMs due to its multi-centered bonding characteristics and significant charge-transfer nature [16,19,23,24,25].The metallic sites can be electronegative and thus can offer improved intrinsic electrocatalytic kinetics [26]. In addition, the insertion of B into the TM matrix can stabilize the atomic configurations and thus can offer enhanced stability [27]. However, the combination of B and P together with the TM has been very rarely studied up to now. As an example, the Co-B-P catalyst demonstrated significantly improved adsorption and desorption capabilities due to the faster charge-transfer kinetics [19], and the good balance between B and P in the TM matrix can induce interesting synergetic effects such as lowering the reaction-energy barrier and increasing the rate of the catalytic process.

At the same time, heteroatom doping is another technique that can be utilized for improved water-splitting performance by increasing the number of active catalytic sites and modifying the electronic states [28,29]. The heteroatom doping of metallic elements such as W, Ru, Mo, Cr and V into the existing material matrix has demonstrated improved water-splitting performances [30,31,32,33]. Among these, V doping is a promising candidate due to its multiple valence states ranging from ^+^2 to ^+^5 that can induce strong electronic interactions with other metal cations. Thus, the incorporation of V can facilitate improved intrinsic catalytic activity by increasing the number of active sites and the structural flexibility, resulting in improved overall water-splitting performances [34]. In addition, V-doping has been barely studied. To this end, the fabrication of well-balanced B and P with Fe and the incorporation of V into the FeBP matrix can be an interesting attempt at improving HER and OER kinetics. With the V-doped FeBP, enhanced electrode stability can be obtained by protecting the metallic cores due to the difference in the electronegativity [35,36]. The V-doped FeBP electrocatalysts can be a cost-effective water-splitting electrocatalyst.

In this study, the FeBP electrocatalyst was fabricated by the systematic parameter control of the hydrothermal approach, and then the heteroatom doping of vanadium was demonstrated by the soaking approach as seen in Figure S1 for efficient overall water splitting for the first time. The V-FeBP MSC demonstrates a bi-functional capability for the HER and OER operations. The two-electrode configuration of V-FeBP || V-FeBP shows a comparable performance as compared to the benchmark electrodes of Pt/C and RuO_2_ in 1 M KOH. The V-FeBP MSC demonstrates the low 2-E overpotential of 1.48 V as compared to the 1.46 V of Pt/C and RuO_2_. In addition, the 2-E system demonstrates nearly the same performance in real sea water as compared with the benchmarks.

## 2. Experimental Section

### 2.1. V-FeBP Electrode Fabrication

For the fabrication of the V-FeBP electrode, nickel foam (NF) was used as a substrate after the ultrasonication in 6 M HCl for 20 min. Figures S2 and S3 show the morphological and elemental analyses of the bare NF. The Fe(NO_3_)_3_·9H_2_O, H_3_BO_3_ and NaH_2_PO_2_·H_2_O were utilized as precursors for the Fe, B and P. The chemicals utilized for the fabrication of the V-FeBP electrode were analytical grades of high purity (Sigma-Aldrich, St. Louis, MO, USA). The FeBP electrode was firstly optimized by the hydrothermal approach in terms of the molarity of precursors, concentration ratio, reaction time, and temperature. The CH_4_N_2_O (urea) was utilized as a surface-active agent to induce the 3D microstructure formation. The precursor solution was placed into a Teflon-lined autoclave with the NF, which was followed by baking at different temperatures and durations. After the FeBP electrode optimization, the vanadium (V) was doped by a soaking approach. The V concentration, soaking duration and temperature were considered for the V-doping optimization.

### 2.2. Morphological, Elemental, and Optical Characterizations

A scanning electronic microscope (SEM, COXEM, Daejeon, Korea) was utilized for the morphology analyses of the various FeBP and V-FeBP electrodes. Energy-dispersive X-ray spectroscopy (EDS, Thermo Fisher, Waltham, MA, USA) was adapted to characterize the elemental phases of the electrodes. The Raman measurement was performed in a NOST system (Nostoptiks, Gyeonggi-do, Korea), equipped with a 532 nm laser, spectrograph (ANDOR, SR-500, Belfast, UK), and charge-coupled device (CCD). The X-ray diffraction (XRD, D8 Advance, Bruker, Billerica, MA, USA) patterns were collected under the illumination of Cu Kα (λ = 1.5406 Å) at a scan rate of 2 °/min.

### 2.3. Electrochemical Characterization

The 3-electrode (3-E) electrochemical characterizations of the FeBP and V-FeBP electrodes were performed with the target electrode as a working electrode, Pt plate as a counter electrode and Ag/AgCl as a reference in an electrochemical workstation (Wizmac, Daejeon, Korea). The reversible hydrogen electrode (RHE) potential (E) was based on the following relation for the HER and OER: E [V vs. RHE] = E + 0.059 × pH + 0.197 (Ag/AgCl). The polarization curves were obtained using linear-sweep voltammetry (LSV) at a scan rate of 5 mV/s between 0.2 and −0.6 V for the HER and 1.2 and 2.2 V for the OER in 1 M KOH. No iR compensation was adapted in any of the electrochemical characterizations and the data were plotted as received. The electrochemical impedance spectroscopy (EIS) was measured in the range of 100 kHz to 0.1 Hz at the voltage corresponding to 10 mA/cm^2^ vs. RHE for the HER and OER catalytic turnover region with an amplitude of 5 mV as shown in Figure S4. Cyclic voltammetry (CV) was performed at different scan rates ranging from 40 to 180 mV/s in a non-faradic region between 0.1 and 0.3 V for the HER and 1.04 and 1.14 V for the OER. From the CV plots, the anodic and cathodic currents were obtained at specific potentials for HER and OER. The electrochemical double-layer capacitance (*C_dl_*) plots were obtained based on ΔJ = (J_a_ − J_c_)/2 as shown in Figures S5 and S6. The slope of the *C_dl_* plot was used to estimate the electrochemical surface-active area (ECSA) in Figure S7. The different *C_dl_* values for the HER and OER reactions suggest different reaction processes. In addition, the 3-E and 2-E water-splitting performances were measured in different pH waters using 1 M KOH (alkaline), 0.5 M H_2_SO_4_ (acidic), and 1 M PBS (neutral). The natural sea water was collected from the Yellow Sea (Incheon, Korea) and river water was obtained from the Han River (Seoul, Korea).

## 3. Results and Discussion

**Figure 1:**
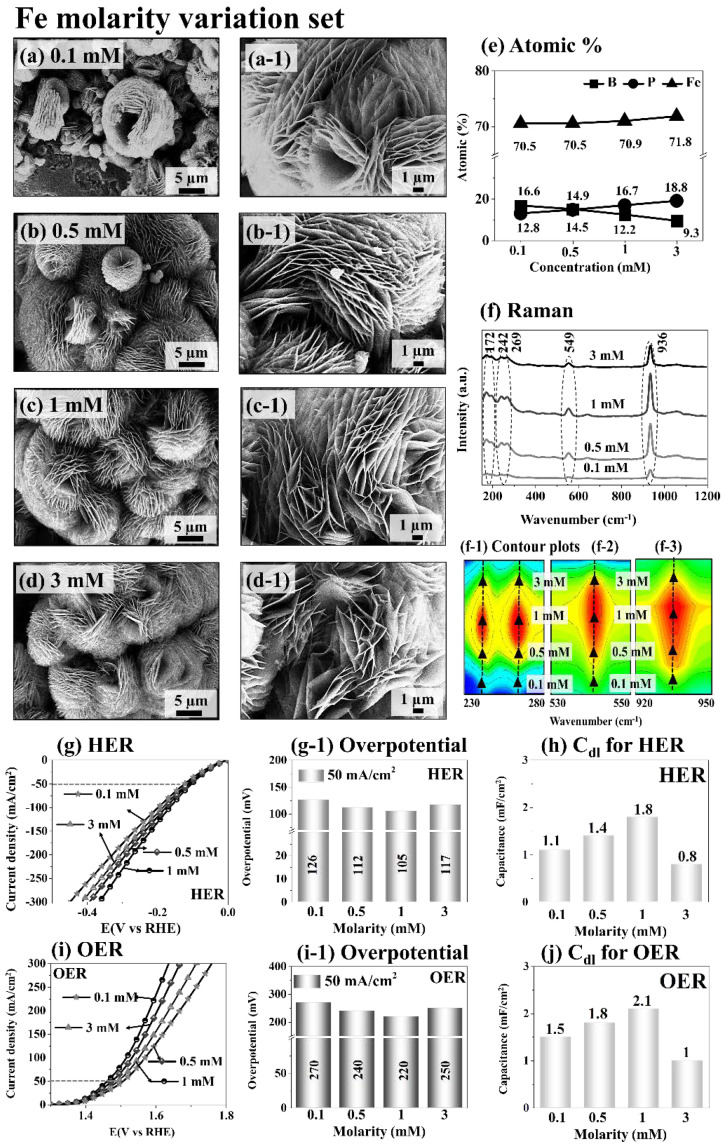
Fe concentration variation between 0.1~3 millimolar (mM) for the fabrication of FeBP MSC electrocatalysts at 100 °C for 12 h. A total of 20 mmol of CH_4_N_2_O, 6 mM NaH_2_PO_2_.H_2_O and 6 mM H_3_BO_3_ were used. (**a**–**d**) SEM images of FeBP electrodes. (**a-1**–**d-1**) Magnified SEM micrographs. (**e**) Atomic% graph. (**f**) Raman spectra of FeBP. (**f-1**–**f-3**) Contour plots of Raman peaks. (**g**,**i**) LSV measurements in 1 M KOH. (**g-1**,**i-1**) Overpotential bar chart at 50 mA/cm^2^. (**h**) and (**j**) C_dl_ values for HER and OER.

In this work, the FeBP electrodes were firstly optimized, and the vanadium (V) doping was optimized on the best FeBP. Firstly, the Fe concentration (FeN_3_O_9_·9H_2_O) variation was performed between 0.1 and 3 mM for the FeBP electrode optimization as shown in Figure 1. Generally, the microspherical structures were fabricated as seen in Figure 1a–d and larger-scale images can be found in Figure S8. The microspheres were constructed with highly dense layers of croissant-like structures as seen in Figure 1(a-1–d-1). Thus, it was named ‘microsphere croissant (MSC)’ for the various layers of croissant bread. Along with the increased FeN_3_O_9_·9H_2_O concentration, the density of MSCs was gradually increased as seen Figure 1a–d and Figure S8. The size of the MSC was up to 20~30 µm. The MSC morphology with the layer-like structures can be largely advantageous for catalytic reactions due to the significantly increased surface area, allowing effective ion access and reactions [37]. The formation of the highly layered 3D structure of Fe-B-P electrocatalyst can be described as below in Equations (1)–(3).

Fe(NO_3_)_3_·9H_2_O + H_3_BO_3_ + NaH_2_PO_2_·H_2_O + CH_4_N_2_O + NH_4_F + n·H_2_O (Precursor solution)(1)

Fe(NO_3_)_3_·9H_2_O + H_2_O → Fe^3+^ + 3NO_3_^−^ + 10H^+^ + 10OH^−^ (2)

2H_3_BO_3_ + 2H_2_O → 2[B(OH)_4_]^−^ + 2H^+^ (3)

NaH_2_PO_2_·H_2_O → NaH_2_PO_2_ + H_2_O → Na^+^ + H_2_PO_2_^−^ + H^+^ + OH^−^ (4)

NH_4_F +H_2_O → NH_4_^+^ + F^−^ + H^+^ + OH^−^ (5)

CH_4_N_2_O + H_2_O → 2NH_3_ + CO_2_ ↑ → 2NH_3_ + 2H_2_O → 2NH_4_^+^ + 2OH^−^ (6)

The overall deposition process is:

Fe^3+^ + 2[B(OH)_4_]^−^ + H_2_PO_2_^−^ + 3NO_3_^−^ + Na^+^ + 14H^+^ + 14OH^−^ + 3NH_4_^+^+ F^−^ →

Fe-B-P + 3NO_3_^−^ + Na^+^ + 14H^+^ + 22OH^−^ + 3NH_4_^+^ + HF + 2H_2_O (7)

For the fabrication of the FeBP electrode, all the precursors were taken as shown in Equation (1). The Fe, B and P were formed by the corresponding precursors as seen in Equations (2)–(4). The Fe(NO_3_)_3_·H_2_O (iron (III) nitrate nonahydrate) generates the Fe^3+^ in Equation (2) and the H_3_BO_3_ (boric acid) produces the B[(OH)_4_]^−^ as the reaction intermediates in Equation (3). The NaH_2_PO_2_ yields the H_2_PO_2_^−^ complex compound in Equation (4). Then, NH_4_F breaks down into NH4^+^ and F^−^ in Equation (5). The generated NH_4_^+^ ions can help to stabilize the pH in the solution and the highly electronegative F^−^ ions help to form H bonds, which can increase the solution conductivity and increase the reaction speed in the ionic solution. Further, CO(NH_2_)_2_ (urea) splits into 2NH_3_ and CO_2_ ↑ in Equation (6). In this process, the generated 2NH_3_ reacts with the water molecules to produce two ammonium (NH_4_^+^) and two hydroxyl (OH^−^) ions in Equation (6). As discussed, the ammonium and hydroxyl ions can also boost the solution conductivity and reaction speed. Finally, the possible fabrication reaction can be described as shown in Equation (7), where the main precursors take part in the formation of FeBP. The boric acid and the formation of HF during the reaction can react with water (HF + H_2_O → H_3_O + F^−^) and helps to form more hydronium ions. During the reaction, the formation of hydronium ions induces the formation of bubbles. The bubble formation helps in layered crystal growth that offers a highly electrochemically active surface area.

**Figure 2:**
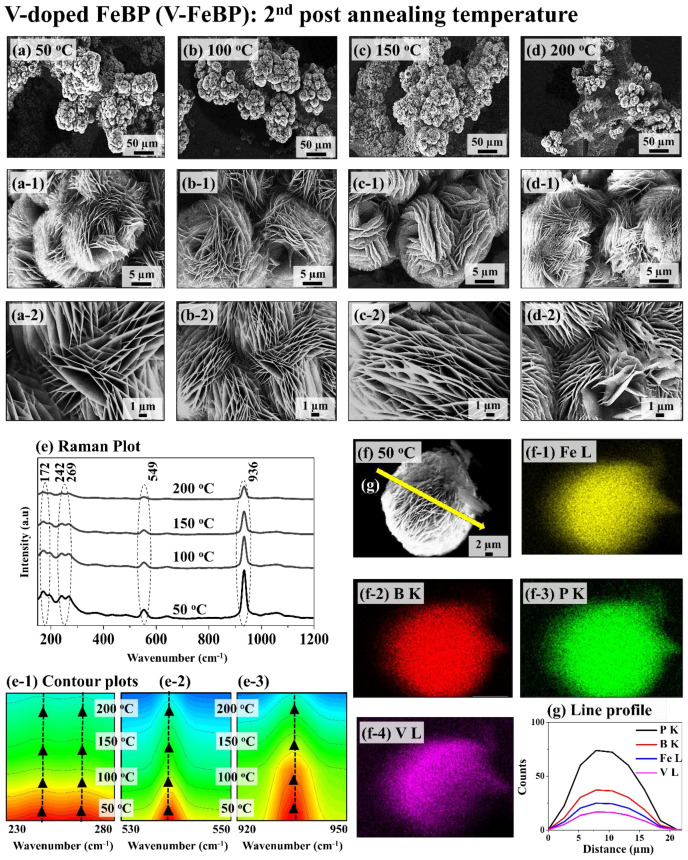
Post-annealing temperature variation (2nd annealing) of vanadium (V)-doped FeBP MSC electrodes between 50~200 °C for 15 min. The best FeBP was fabricated at 100 °C for 12 h with the 1 mM Fe, 3.6 mM H_3_BO_3_, 8.4 mM NaH_2_PO_2_ and 20 mM CH_4_N_2_O. The best FeBP was post-annealed (1st) and doped with V by a soaking approach in 0.2 mM V solution for 15 min at room temperature. (**a**–**d**) SEM micrographs. (**a-1**–**d-1**,**a-2**–**d-2**) Magnified SEM images. (**e**) Raman plot. (**e-1**–**e-3**) Contour plots of Raman peaks. (**f**–**f-4**) EDS maps of Fe L, B K, P K and V L. (**g**) Corresponding EDS line profiles.

**Figure 3:**
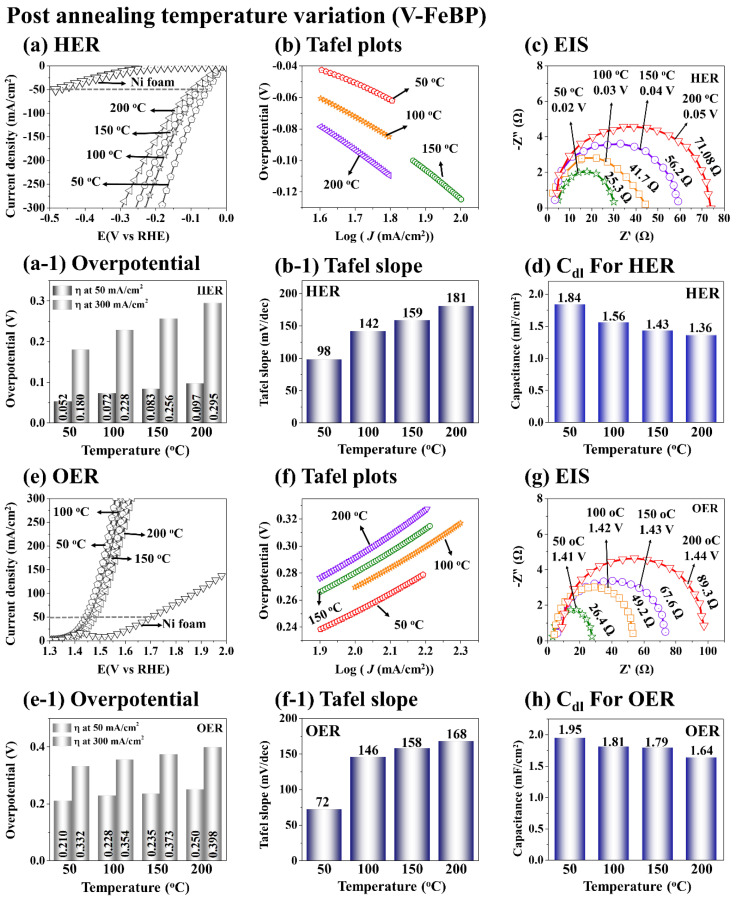
3-electrode (3-E) electrochemical performance of the post-annealing temperature variation set (V-FeBP MSC electrodes). (**a**,**e**) HER and OER polarization curves of V-FeBP electrodes in 1 M KOH. (**a-1**,**e-1**) Overpotentials at 50 and 300 mA/cm^2^. (**b**,**f**) Tafel slopes derived from the polarization curves. (**b-1**,**f-1**) Tafel slope values. (**c**,**g**) HER and OER Nyquist plots obtained at the fixed current of 20 mA/cm^2^. (**d**,**h**) HER and OER *C_dl_* values. All the electrochemical measurements were plotted as received without iR drop compensation.

Figure 1e presents the At.% plots of Fe, B and P in the Fe concentration variation set. The At.% showed a gradually increased incorporation of Fe atoms with the increased Fe molarity. B was also more incorporated. However, P showed a gradually decreased incorporation, perhaps due to the high affinity of Fe and B. Additional full-range EDS spectra are provided in Figure S9. Figure 1f shows the Raman spectra of FeBP MSCs with the characteristic peaks at 172, 242, 269, 549, and 936 cm^−1^. The Raman contour plots are shown in Figure 1(f-1–f-3). The highest Raman peaks demonstrated by the 1 mM Fe indicates the highest local crystallinity of FeBP MSC structures. Raman intensity was decreased for the other Fe concentrations. The highly local-crystalline FeBP MSC with microsheets can provide faster electron transfer and increased intrinsic electrocatalytic activity by lowering charge-transfer resistance [13]. In terms of electrochemical performance, the HER and OER LSV curves of the FeBP MSC electrodes are provided in Figure 1g,i with the corresponding overpotential values in Figure 1(g-1,i-1). The HER reaction in an alkaline medium can be described by the Volmer, Heyrovsky and Tafel steps, where the metal active sites can react with the H_2_O and generate a metal–hydride bond to produce H_2_ [35,38]. Volmer step: H_2_O + M + e^−^ → M-H* + OH^−^, Heyrovsky step: M-H* + H_2_O + e^−^ → M + OH^−^ + H_2_, Tafel step: 2M-H* → 2M + H_2_. The Volmer reaction is the production of M-H*, followed by the Heyrovsky step. The Tafel steps explain the whole process of producing H_2_. In the water electrolysis process, the HER is a crucial half-reaction to produce hydrogen at the cathode through a two-electron transfer process with the generation of hydroxyl (OH^−^) ions. In contrast, the OER entails four-proton–electron transfer reactions at the anodic metallic atomic sites [39]. OH^−^ + * → HO* + e^−^, HO* + OH^−^ → O* + H_2_O + e^−^, O* + OH^−^ → HOO* + e^−^, HOO* + OH^−^ → * + O_2_(g) + H_2_O + e^−^. Starting from the hydroxyl (OH^−^) generated from the HER, O_2_ is evolved through the protonation of HOO* coupled with the regeneration of 2H_2_O at the active sites. In general, the electrical current splits the water molecules into hydrogen and oxygen in alkaline water electrolysis in the presence of metal (M) sites [39]. In the HER and OER reactions, the strong M-H* and M-OH bindings are the key components of the catalytic surface and thus, the strong binding nature of H atoms and hydroxyl ions with a large surface area is important in water electrolysis. The FeBP MSC with the 1 mM Fe demonstrated the best HER and OER performances with the lowest overpotential of 105 and 220 mV at 50 mA/cm^2^, as summarized in Figure 1(g-1,i-1). The 1 mM Fe demonstrated the highest double-layer capacitance (C*_dl_*) values of 1.8 and 2.1 mF/cm^2^ for the HER and OER, as seen in Figure 1h,j, which suggests the largest electrochemical surface area (ECSA) of the 1 mM Fe. The improved performance of the FeBP MSC can be attributed to the improved local-crystalline quality and the balance between the ternary Fe, B and P elements with the MSC morphology, which can boost the catalytic activity in an alkaline environment [40]. The MSC structure formed with the appropriate number of Fe, B and P atoms can offer rich active sites for the H and OH^−^ groups, and such a hierarchical structure can benefit the high reaction rate due to the large electrochemical surface area and the acceleration of charge transfer [16]. In addition, the P and B groups can act as electron donors to the d-orbitals of transition metals in the FeBP system, resulting in a high electron concentration of the Fe atoms, which can lower the reaction barriers for the H_2_O and OH^−^ [33].

In addition to the Fe concentration variation (related data Figures S5–S10), the 100 °C reaction temperature (related data Figures S11–S14), 20 mM urea (related data Figures S15 and S16), 30% B and 70% P (FeB_30_P_70_) (related data Figures S17–S21), and 100 °C post-annealing treatment (related data Figures S22–S26) were found to offer the best optimized performance. Figure S19 shows the XRD patterns of FeBP, FeB and, FeP. The two common peaks at 44.4 and 51.8° correspond to the (111) and (200) planes of the nickel substrate in the XRD patterns [41]. Generally, the FeBP showed broader peaks with a lower intensity as compared with the FeP and FeB in Figure S19, which could be due to the increase in the short-range polycrystalline phases of FeBP. Generally, the polycrystalline phase can indicate a low electron transfer and high resistance. Thus, a lower electrochemical performance can be expected. However, a recent study showed that the short-range polycrystalline or amorphous phases can be beneficial to the improved electrochemical performance in water electrolysis [42,43]. The polycrystalline phase can offer abundant active sites and higher intrinsic electrochemical activity due to the structural flexibility and stability of the electrocatalysts.

**Figure 4:**
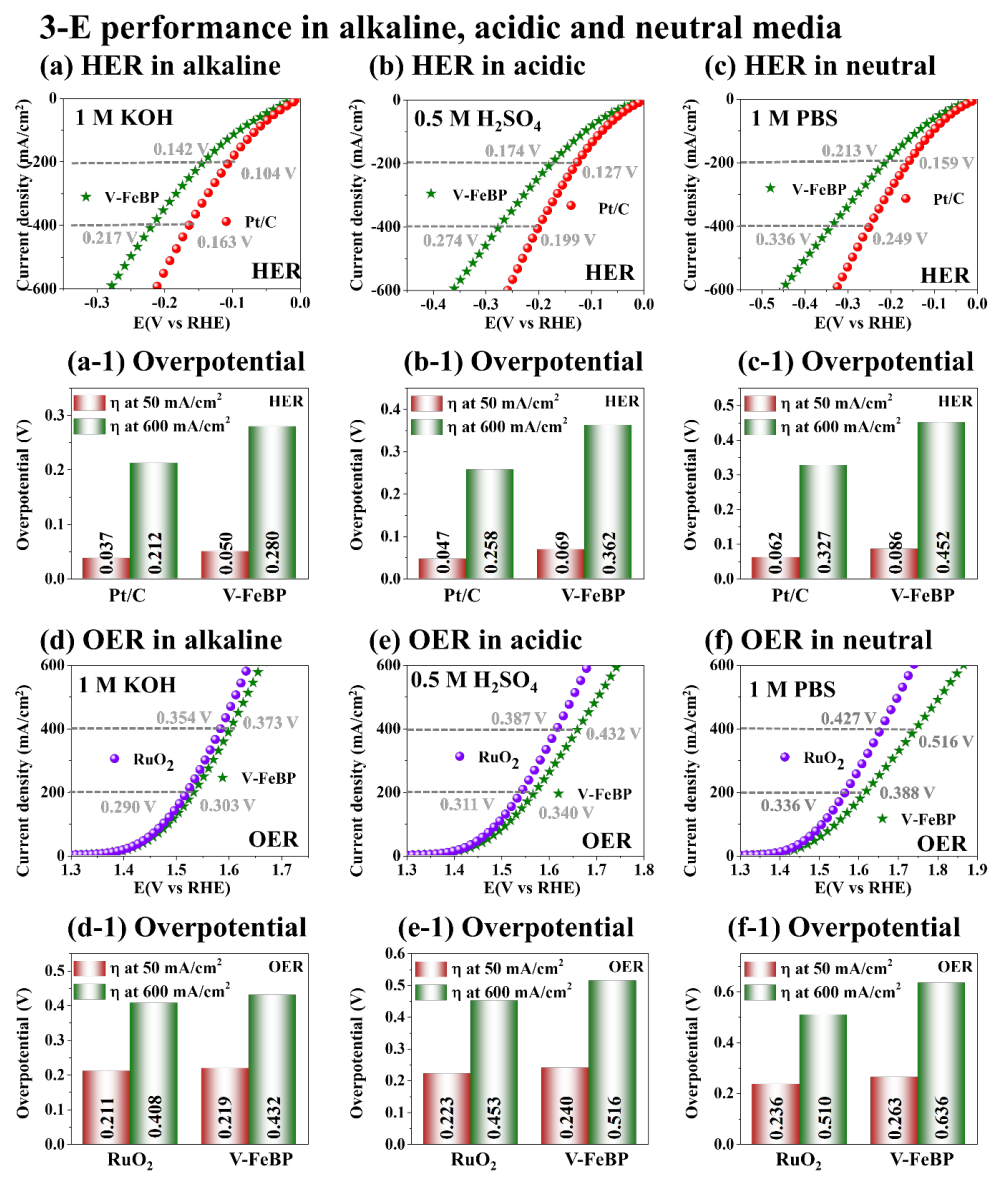
3-E electrochemical performance comparison of V-FeBP and benchmark electrodes (Pt/C and RuO_2_) in alkaline, acidic, and neutral waters. (**a**–**c**) HER curves in 1.0 M KOH, 0.5 M H_2_SO_4_, 1 M PBS. (**a-1**–**c-1**) Corresponding overpotential bar plots at 50 and 600 mA/cm^2^. (**d**–**f**) HER curves. (**d-1**–**f-1**) Corresponding overpotential bar plots.

Figure 2 shows the vanadium (V)-doped FeBP MSC electrodes after the 2nd-stage post-annealing temperature optimization. The best FeBP electrode was taken for the vanadium (V) doping by controlling the doping temperature between 25 and 80 °C in Figures S27–S29, the vanadium concentration between 0.05 and 0.4 mM in Figures S30–S32, and the soaking duration between 15~120 min in Figures S33–S35. The 0.2 mM V in the 15 min soaking reaction at 25 °C demonstrated the best HER and OER performances in Figure S35 without any change in the morphology. An adequate amount of V incorporation can induce the water-dissociation capacity and can decrease the energy barrier and reduce the impedance of charge transfer. In addition, post-annealing at an appropriate temperature can improve the crystallinity of electrodes by the reduction in point and line defects with the thermal diffusion of atoms [44,45]. In terms of the 2nd post-annealing duration optimization, the 15 min duration showed the best result in Figures S36–S38. Along with post-annealing at various temperatures for 15 min, the 50~100 °C samples showed similar morphologies before and after the annealing in Figure 2(a–b-2). However, the high-temperature-annealed samples showed a slight deformation of croissant layers at 150 °C and more deformation at 200 °C in Figure 2(c-2–d-2). The temperature of 200 °C also had a much lower density of the microsphere croissant (MSC) in Figure 2d. Excess diffusion energy at a high temperature can damage crystallinity due to defect formation and can separate the MSC from the NF during the annealing process. Further, the Raman analyses demonstrated the best intensity with the 50 °C sample, as clearly seen in Figure 2(e–e-3). It clearly demonstrates that the 50 °C sample had better crystallinity, which helps to obtain stable electrochemical activity. In addition, the 50 °C sample demonstrated uniform distributions of Fe L, B K, P K, and V L peaks, indicating the even diffusion of vanadium into the FeBP matrix, as shown by the EDS maps and line profiles in Figure 2(f–f-4,g).

Figure 3 shows the electrochemical characterizations of the V-FeBP MSC electrodes at the post-annealing optimization in terms of LSV, Tafel, EIS and C_dl_. As shown in Figure 3a,e, the V-FeBP electrode annealed at 50 °C demonstrated the best HER and OER performances, and the performance gradually became worse with the increased temperature. The 50 °C sample demonstrated the lowest overpotentials of 52 mV and 210 mV at 50 mA/cm^2^ for the HER and OER, as summarized in Figure 3(a-1,e-1). The bar plots in Figure 3(a-1,e-1) clearly show the overpotential values, which followed the sequence of 50 < 100 < 150 < 200 °C. The improved HER and OER performances could be due to the reduced lattice defects and better electrocatalytic activity following the appropriate heat treatment for an appropriate duration [37]. After the V doping and post-annealing optimization, the surface structure of the electrode can reorganize, and thus can introduce more active sites on the catalytic surface [46]. Additional active sites can speed up the electrochemical HER and OER reaction processes by increasing the conductivity to obtain better HER and OER performances. The V doping of the FeBP can largely improve the conductivity and electron density to enhance the electrocatalytic reaction. The addition of V can tune the electronic structure and activate more active sites. The partial electron transfer is possible from the V^2+^ to Fe^2+^ ions, which might help to improve the adsorption capacity of hydrogen protons and hydroxyl groups and improve the HER and OER processes [47]. The HER and OER Tafel analyses are shown in Figure 3b,f. The Tafel slopes can be acquired from the linear range of the HER and OER curves as shown in Figure 3b,f. The Tafel slope values in Figure 3(b-1,f-1) indicate the degree of the reaction and charge-transfer rates. The lower slope values indicate a higher electron transfer and thus a greater reaction rate. The 50 °C sample demonstrated the lowest HER and OER Tafel slope values of 98 and 72 mV/dec, as summarized in Figure 3(b-1,f-1). The HER and OER EIS measurements were performed to understand the transport characteristics of the V-FeBP electrodes. The HER and OER EIS were measured at different overpotential voltages based on the fixed current of 20 mA/cm^2^ for the consistency between samples. The EIS measurements showed different R_ct_ values at different voltages around the turnover region, as seen in Figure S4 [1]. The higher voltage application showed smaller R_ct_ values and vice versa. In both the HER and OER EIS plots, the charge-transfer resistance (R_ct_) was gradually decreased with the lower annealing temperatures, and the V-FeBP electrode annealed at 50 °C demonstrated the lowest (R_ct_) of 25.3 and 26.4 Ω for the HER and OER EIS, which indicates that the 50 °C sample demonstrated the lower conductivity and outstanding charge-transport characteristics [1]. Further, the double-layer capacitance (C*_dl_*) measurements based on the CV plots indicated the highest electrochemical active surface area of the 50 °C sample with 1.84 and 1.95 mF/cm^2^ in Figure 3d,h. After doping, the electrochemical surface area of the V-FeBP MSC was significantly increased, indicating a higher electrochemical activity of electrode. The CV curves and anodic and cathodic current densities are provided in Figures S40 and S41.

**Figure 5:**
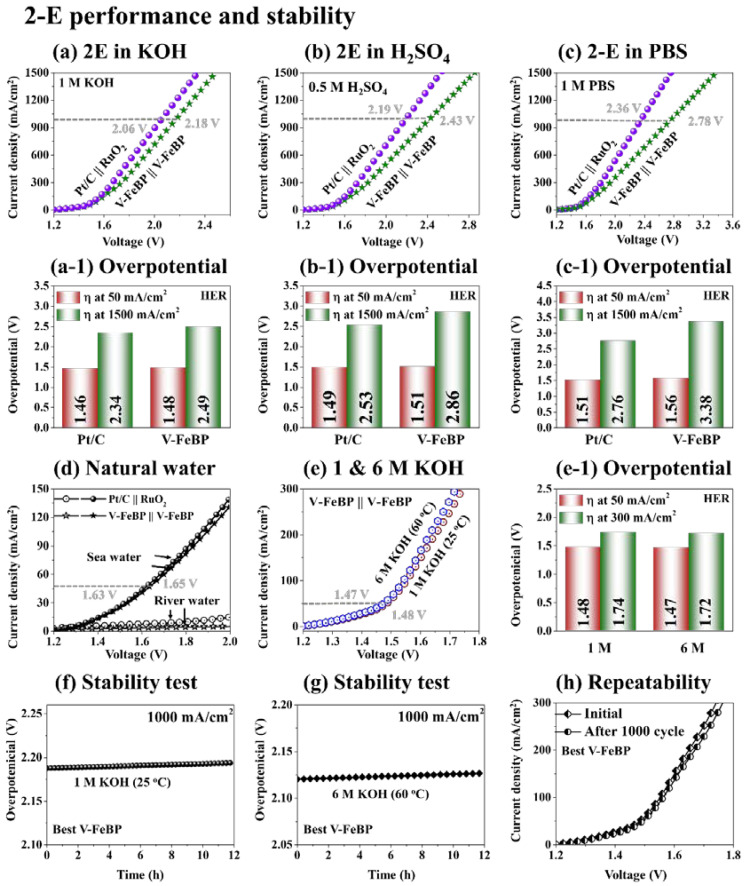
2-E electrochemical performance in alkaline, acidic and neutral media and stability test. (**a**–**c**) 2-E LSV curves in 1 M KOH, 0.5 M H_2_SO_4_ and 1 M PBS. (**a-1**–**c-1**) Overpotentials at 50 and 1500 mA/cm^2^. (**d**) 2-E LSV plots in natural sea and river waters. (**e**) 2-E LSV in 1 and 6 M KOH. (**e-1**) Overpotentials at 50 and 300 mA/cm^2^. (**f**,**g**) Stability test of V-FeBP for 12 h in 1 M (25 °C) and 6 M KOH (60 °C) at 1000 mA/cm^2^. (**h**) 2-electrode repeatability test of V-FeBP before and after 1000 cycles in 1 M KOH.

Figure 4 shows the 3-E electrochemical performance comparison of V-FeBP and benchmark electrodes of Pt/C and RuO_2_ in alkaline, acidic, and neutral waters. Different pH waters were prepared by 1 M KOH (pH 14), 0.5 M H_2_SO_4_ (pH 0) and 1 M PBS (pH 7.4). The morphological and elemental analyses of the benchmark electrodes of Pt/C and RuO_2_ are provided in Figures S42 and S43. Overall, the V-FeBP and benchmark electrodes demonstrated quite stable operations in alkaline, acidic, and neutral waters in Figure 4a–f. At the same time, the benchmark electrodes demonstrated better HER and OER performances in all three solutions, as clearly seen in Figure 4(a-1–f-1). Both V-FeBP and the benchmark electrodes demonstrated similar trends in terms of performance with the overpotentials in alkaline < acidic < neutral waters, indicating that both electrode configurations demonstrated the best performances in 1 M KOH water. The higher performance in KOH can be attributed to the high electrochemical conductivity due to the ionization of OH^−^ [48]. KOH can offer high current density and electrode stability. In the electrochemical reaction process, the cation K^+^ plays a crucial role in lowering the activation barrier for the dissociation of H_2_O into OH^−^ + H^+^ + e^−^. KOH dissociates into K^+^ and OH^−^ in water and H_2_O can be dissociated more easily into OH^−^ and H^+^ [49]. The lower HER and OER performances in the acidic solution could be due to the slow reaction rate with the electrode degradation in the low-pH water [50]. Similarly, the lowest performances in the neutral media could be due to the low ion migration in PBS solution, which could have resulted in the lowest kinetics during the HER and OER operations [3]. The lack of hydrogen protons or hydroxyl ions can obstruct the mass transport and cause extra energy consumption to dissociate water molecules under neutral conditions [51]. In short, the V-FeBP demonstrated good electrochemical performances with all the optimizations in terms of the LSV, Tafel, EIS, C_dl_, TOF and stability. This could be due to the good balance between the V, Fe, B and P components and the good crystalline quality, along with the unique microsphere croissant (MSC) morphology as discussed. Additionally, the HER and OER steady-state current observations were performed by the comparison of the LSV and CA currents in a 3-E system in Figures S44 and S45. This was to show the stability of the electrodes at different current densities [52]. The V-FeBP annealed at 50 °C demonstrated stable operations at various voltages as summarized in Figures S44 and S45, indicating a good stability of the V-FeBP electrode. One thing to notice here is that the V-FeBP achieved a comparable OER result in 1 M KOH in Figure 5d, indicating that the 2-E operation of V-FeBP electrodes can largely benefit from the good OER performance.

Figure 5 shows the 2-E electrochemical performance of V-FeBP and benchmark electrodes in alkaline, acidic, and neutral media and the stability test. In the 2-E configuration, the Pt/C ‖ RuO_2_ were used as the cathode and anode, and two V-FeBP electrodes were adapted as bi-functional electrodes, i.e., V-FeBP ‖ V-FeBP. Generally, the 2-E water-splitting performance trend was similar to the 3-E, i.e., alkaline < acidic < neutral waters, in Figure 5a–c. The specific overpotentials at 50 and 1500 mA/cm^2^ are summarized in Figure 5(a-1–c-1). The overpotentials were 1.46 and 1.48 V at 50 mA/cm^2^ and then reached 2.34 and 2.49 V at 1500 mA/cm^2^ in 1 M KOH for the Pt/C ‖ RuO_2_ and V-FeBP ‖ V-FeBP in Figure 5(a-1). The overpotentials were 1.49 and 1.51 V at 50 mA/cm^2^ and 2.53 and 2.86 V at 1500 mA/cm^2^ in 0.5 M H_2_SO_4_ in Figure 5(b-1). Similarly, the overpotentials were 1.51 and 1.56 V at 50 mA/cm^2^ and 2.76 and 3.68 V at 1500 mA/cm^2^ in 1 M PBS in Figure 5(c-1). The benchmark configuration demonstrated better water-splitting performances over the V-FeBP ‖ V-FeBP configuration due to the superior intrinsic electrochemical properties of Pt/C and RuO_2_ for the HER and OER operations. Notably, the bi-functional configuration of V-FeBP demonstrated 2.18 V as compared with 2.06 V of the Pt/C ‖ RuO_2_ at 1000 mA/cm^2^ as identified in Figure 5a, which is a quite comparable performance to the benchmarks. This indicates that V-FeBP ‖ V-FeBP can demonstrate a compatible water-splitting performance as compared with the Pt/C ‖ RuO_2_ in 1 M KOH water, with the costs of the electrode materials being several orders less.

The 2-E performance of V-FeBP ‖ V-FeBP and Pt/C ‖ RuO_2_ in natural sea and river waters are shown in Figure 5d. The V-FeBP ‖ V-FeBP demonstrated a comparable overpotential of 1.63 V at 50 mA/cm^2^ as compared to the 1.65 V of Pt/C ‖ RuO_2_ in sea water. The river water showed a very low current for both electrode configurations. The sea water generally demonstrated a better performance due to the presence of numerous Na^+^ and Cl^−^ ions, which can increase the conductivity in the water, and thus the water-splitting performance can be improved. Meanwhile, the river water also includes various kinds of ion species such as Ca^+^, Mg^+^, Br^−^, HCO_3_^−^, SiO_2_, SO_4_^−^, Cl^−^, F^−^, etc. [53]. These anions and cations in the river water can slow down the reaction process and lower the overall current density. While the elemental compositions in both sea and river waters are similar, the majority of the ionic species in sea water ions are Na^+^ and Cl^−^ (over 90%) and HCO_3_^−^, Ca^+^, SiO_2_, SO_4_^−^ constitute over 90% of the ionic species in river waters. In addition, the V-FeBP ‖ V-FeBP demonstrated a slightly improved water-splitting performance in 6 M KOH at 60 °C as compared to the 1 M KOH at 25 °C as seen in Figure 5e. The overpotential values are shown in Figure 5(e-1). The V-FeBP ‖ V-FeBP demonstrated quite a stable current in 1 M KOH at 25 °C and in 6 M KOH at 60 °C at 1000 mA/cm^2^ in Figure 5f,g, which indicates a good stability of V-FeBP in industrial water-splitting conditions. The stability test at the high current of 1000 mA/cm^2^ for 12 h did not show any significant difference, but there was a slightly increasing trend, likely due to the oxidation of metallic atoms and surface modifications, as shown in Figure 5f. Similarly, the chronoamperometry test did not show any degradation in the harsh industrial condition of 6 M KOH, indicating the excellent stability of V-FeBP, as shown in Figure 5g. The V-FeBP ‖ V-FeBP also demonstrated excellent repeatability after 1000 cycles in 1 M KOH, as shown in Figure 5h. The two-electrode activity after 1000 cycles showed a very negligible difference in performance, which clearly shows that the V-FeBP has good repeatability after a long operation. In addition, the HER and OER turnover frequency (TOF) of the post-annealing temperature variation set of the V-FeBP electrocatalysts was evaluated for the vanadium and iron active sites at 150 mV/cm^2^, as shown in Figure S46. The TOF indicates the number of H_2_ and O_2_ molecules generated per atomic site per unit of time at the turnover. The TOF can be used to indicate the intrinsic water-splitting activity of each catalytic atomic active site under a specified reaction condition [38,54]. As summarized in Figure S46, the V-FeBP annealed at 50 °C demonstrated the highest HER and OER TOF values of 3.32 and 2.10 site^−1^ s^−1^. In addition, the 2-E LSV and CA comparison of V-FeBP ‖ V-FeBP is shown in Figure S47, and the steady-state LSV and CA currents showed minor differences, as summarized in Figure S47c,d, indicating a good stability and stable operations at various voltages. The comparison of the two-electrode performance with the state-of-the-art Fe-based electrodes and transition-metal-based electrodes at 50 mA/cm^2^ in 1 M KOH are shown in Figures S48 and S49 and Table 1 and Table S1. The V-FeBP was 2nd in the overpotential comparisons. Further, the three-electrode comparison with the state-of-the-art transition-metal-based electrodes at 10 mA/cm^2^ in 1 M KOH is summarized in Figure S50 and Table S2. Again, the V-FeBP was one of the best.

## 4. Conclusions

A unique microsphere croissant (MSC) configuration of a V-FeBP electrode was demonstrated on a form of bare nickel substrate. The FeBP MSC was first optimized in terms of various synthesis parameters, and then the vanadium doping was further optimized. Generally, the well-balanced F-B-P elements showed better electrochemical performances over the FeB and FeP. The post-annealing played an important role in improving the crystallinity of FeBP MSCs. Overall, the V-FeBP electrode demonstrated quite a comparable performance as compared with the benchmark electrodes with the low overpotential of 52 and 210 mV at 50 mA/cm^2^ for the HER and OER in a three-electrode configuration in 1 M KOH. The V-FeBP || V-FeBP also demonstrated a comparable overpotential of 1.48 V at mA/cm^2^ as compared with the PtC || RuO_2_. This clearly indicates that V-FeBP can offer a compatible water-splitting performance in 1 M KOH water. In addition, the V-FeBP MSC demonstrated excellent stability and repeatability under industrial water-splitting conditions. This study presents an efficient approach based on the combination of the transition metal Fe combined with the non-metallic elements B and P, and the heteroatom doping of V, which can offer an alternative option for large-scale water electrolysis.

## Figures and Tables

**Table 1:** Comparison of 2-electrode performance with the state-of-art Fe-based electrodes at density of 50 mA/cm^2^ in 1 M KOH.

Electrocatalysts	Electrolyte Solution	Overpotential [V] at 50 mA/cm^2^	Year	Reference
**FeNiSe**	1 M KOH	1.36	2022	[55]
**V/FeBP**	1 M KOH	1.48	-	(This work)
**Ni-Fe-MoN NTs**	1 M KOH	1.62	2018	[56]
**NiFe LDH@DG10**	1 M KOH	1.65	2017	[57]
**Fe-Ni_5_P_4_/NiFeOH-350**	1 M KOH	1.66	2021	[9]
**Fe_7_._4%_-NiSe**	1 M KOH	1.68	2019	[58]
**NFC@CNSs-700**	1 M KOH	1.70	2021	[59]
**R-Fe-Ni_2_P**	1 M KOH	1.75	2020	[60]
**(FeO)_2_.(MoO_2_)_3_/MoO_2_**	1 M KOH	1.76	2020	[61]
**Ni-Fe-P/NF_0_**	1 M KOH	1.77	2019	[62]
**Fe-Cu@CN3**	1 M KOH	1.83	2021	[63]
**Fe-Ni_3_ S_2_/NF**	1 M KOH	1.84	2020	[64]
**CoFeO NFs/NPCNT**	1 M KOH	1.86	2019	[65]

## Data Availability

The data presented in this study are available on request from the corresponding author.

## Supplementary Materials

The following are available online at https://www.mdpi.com/article/10.3390/nano12193283/s1, Figure S1: (a) Schematic representation of V-doped FeBP microsphere croissant (MSC) structure fabrication, namely V-FeBP MSC electrocatalyst. The V-FeBP MSC were fabricated by the combination of hydrothermal method and soaking approach. (a-1) V-doped FeBP MSC by the soaking approach., Figure S2. (a)–(c) SEM images of bare Ni foam (NF), Figure S3. (a)–(a-2) EDS maps of Ni and O. (b)–(b-1) Elemental line-profiles from the arrow location in (a). (c) EDS spectrum and atomic percentage of NF, Figure S4. Electrochemical impedance spectroscopy (EIS) measured at different voltages for the best V-FeBP. (a) EIS for HER. (b) EIS for OER. The EIS measurements showed different R_ct_ values at different voltages around the turnover region. The higher voltage application showed the smaller R_ct_ values and vice versa. Thus, the EIS was measured at a fixed current of 20 mA/cm^2^ for the consistency between samples. The EIS was measured between 100 kHz to 0.1 Hz with an amplitude of 5 mV, Figure S5. (a)–(d) HER CV curves of various FeBP electrocatalyst with Fe concentration variation set. The CV curves were measured in the non-faradic region where there is no charge transfer reaction occur in between 0.2 and 0.3 E below 1.023 V based on the E_RHE_ = E + 0.059 × pH + 0.197 (Ag/AgCl). The actual reverse sweeping voltage (E) lies between −0.723 and −0.823 V. The scan rate was varied from 40 to 180 mV/s at the interval of 20 mV/s, Figure S6. (a)–(d) OER CV curves of various V-FeBP catalyst with Fe concentration variation set. The CV scan rate is varied from 40 to 180 mV/s. The CV measurements were taken in a non-faradic region from 1.04 to 1.14 E below 1.23 V based on E_RHE_ = E + 0.059 × pH + 0.197 (Ag/AgCl). The actual forward sweeping voltage (E) was between 0.017 and 0.117 V, Figure S7. (a) and (d) HER and OER anodic and cathodic current density vs scan rate plots. (b) and (e) HER and OER double layer capacitance (*C_dl_*) plots. The *C_dl_* plots were obtained from the anodic and cathodic current density graphs: Δ*j**H_0.25_* = (*Ja* -*Jc*)/2 and Δ*j**O_1.09_* = (*Ja* -*Jc*)/2, where *Ja* and *Jc* is the anodic and cathodic current. (c) and (f) Bar plots of *C_dl_* values. The HER and OER C_dl_ are obtained from the extracting slope of *C_dl_* plots in (b) and (e). The HER and OER *C_dl_* values represent the HER and OER electrochemical surface-active area (ECSA), Figure S8. (a)–(d) SEM images of FeBP electrode fabricated with Fe concentration variation, Figure S9. (a)–(d) EDS spectra of Fe concentration variation set with corresponding atomic percentage in the given table, Figure S10. Reaction temperature variation for the FeBP electrocatalyst fabrication. The reaction temperature is varied between 80 and 140 °C. (a)–(d) SEM images. (a-1)–(d-1) Enlarged SEM images for the corresponding samples. (e) EDS spectra of 100 °C electrode. (f)–(f-3) SEM and corresponding EDS phase maps of Fe, B and P. (g) Raman spectra for FeBP. (h) and (i) Hydrogen evolution reaction (HER) and oxygen evolution reaction (OER) performance in 1 M KOH. (h-1) and (i-1) HER and OER overpotential values at 50 mA/cm^2^, Figure S11. (a)–(c) EDS spectra of reaction temperature variation set. Tables show the atomic and weight percentage of Fe, B and P. (d) Atomic % of Fe, B and P, Figure S12. (a)–(d) HER CV curves of various V-FeBP electrocatalyst with reaction temperature variation set measured in the non-faradic region between 0.2 and 0.3 E. The scan rate was varied from 40 to 180 mV/s at the interval of 20 mV/s, Figure S13. (a)–(d) OER CV curves of various V-FeBP catalyst with the reaction temperature variation. The CV scan rate is varied from 40 to 180 mV/s, Figure S14. (a) and (c) HER and OER anodic and cathodic current density vs scan rate plots. (b) and (d) HER and OER double layer capacitance (*C_dl_*) plots. (c) and (f) Bar plots showing *C_dl_* values, Figure S15. (a) and (b) Polarization curves of HER and OER for the FeBP electrodes with the urea concentration variation, Figure S16. (a)–(c) SEM images of FeBP electrode fabricated with B and P concentration variation. (a-1)–(c-1) Enlarged SEM images for the corresponding electrodes. Other images are in the main figures, Figure S17. B and P concentration variation at 100 °C for 12 h. The total molarity of B and P was fixed at 20 mM and the ratio was varied accordingly: i.e., the B_30_P_70_ indicates 3.6 mM B and 8.4 mM of P. The Fe concentration was fixed at 1 mM. (a)–(d) SEM images for FeBP MSC electrocatalysts. (a-1)–(d-1) Enlarged SEM images. (e) Atomic % plot. (f) Raman spectra. (f-1)–(f-3) Contour plots of Raman peaks. (g) and (i) HER and OER LSV curves. (g-1) and (i-1) Overpotential values at 50 mA/Cm2 for HER and OER. (h) and (j) HER and OER C_dl_ values, Figure S18. (a)–(g) EDS spectra of B and P concentration variation set as labeled and corresponding tables of the atomic and weight percentage of Fe, B and P, Figure S19. X-ray diffraction (XRD) analysis of FeB, FeP and FeBP. Generally, the FeP and FeB demonstrated sharper peaks and the FeBP demonstrated broad peaks. This could be due to the short-range polycrystalline phase of FeBP, Figure S20. (a)–(g) HER CV curves of B and P concentration variation set as labeled. (h) Linear plots for the anodic and cathodic current density versus scan rates of the CV. (i) HER double layer capacitance (*C_dl_*) plots, Figure S21. (a)–(g) OER CV curves of B and P concentration variation set as labeled. (h) Linear plots of anodic and cathodic current density versus scan rates of the CV plot. (i) OER double layer capacitance (*C_dl_*) plots, Figure S22. Post annealing temperature variation between 100 and 500 °C. The best sample (1 mM Fe, 20 mM CH_4_N_2_O, 3.6 mM H_3_BO_3_ and 8.4 mM NaH_2_PO_2_. H_2_O) fabricated at 100 °C for 12 hrs was adapted for annealing. (a)–(d) SEM images. (a-1)–(d-1) and (a-2)–(d-2) Enlarged SEM images. (e)–(e-3) SEM images and its corresponding EDS maps of Fe K, B K and P K. (f) EDS line profile plot corresponds to yellow line. (g) Atomic percentage plot. (h)–(h-3) Raman spectra and contour plots, Figure S23. (a)–(d) EDS spectra of the post-annealing temperature variation set. Inset tables show the atomic and weighting percentages of Fe, B, and P, Figure S24. Electrochemical performance of post annealing temperature variation set. (a) and (e) HER and OER polarization curves of FeBP electrodes in 1 M KOH. (a-1) and (e-1) HER and OER overpotential bar graphs at 50 and 300 mA/cm^2^. (b) and (f) Tafel slopes derived from polarization curves. (b-1) and (f-1) Tafel slope values. (c) and (g) HER and OER Nyquist plots. (d) and (h) HER and OER *C_dl_* values, Figure S25. (a)–(d) HER CV curves of post annealing temperature variation as labeled. (e) Linear plots for the anodic and cathodic current density versus scan rates of the CV. (f) HER double layer capacitance (*C_dl_*) plots, Figure S26. (a)–(d) OER CV curves of post annealing temperature variation as labeled. (e) Linear plots for the anodic and cathodic current density versus scan rates of the CV. (f) OER *C_dl_* plots, Figure S27. (a)–(d) SEM images of V-FeBP electrodes, V-doped with soaking approach at different temperature as labelled. (a-1)–(d-1) Enlarged SEM images of the corresponding electrodes, Figure S28. (a)–(d) EDS spectra and corresponding atomic percentage tables of the V-FeBP electrodes by the soaking temperature variation, Figure S29. (a) and (b) HER and OER polarization curves of the V-FeBP electrodes by the soaking temperature variation. The room temperature (RT) sample demonstrated the best HER and OER performances, Figure S30. (a)–(d) SEM images of V-FeBP electrode fabricated with the V concentration variation. (a-1)–(d-1) Enlarged SEM images for the corresponding electrodes, Figure S31. (a)–(d) EDS spectra and corresponding atomic percentage tables of the V concertation variation set, Figure S32. (a) and (b) HER and OER LSV curves of the V-FeBP electrodes with the V concentration variation. The 0.2 mM sample demonstrated the best HER and OER performances, Figure S33. (a)–(d) SEM images of V-FeBP electrodes fabricated with the soaking duration variation. (a-1)–(d-1) Enlarged SEM images for the corresponding electrodes, Figure S34. (a)–(d) EDS spectra and corresponding atomic percentage tables of the soaking time variation set, Figure S35. (a) and (b) HER and OER polarization curves of the V-FeBP electrodes with the soaking time variation. The 25-min sample demonstrated the best HER and OER performances, Figure S36. (a)–(d) SEM images of V-FeBP electrode fabricated with post annealing duration variation at 100 °C annealing temperature. (a-1)–(d-1) Enlarged SEM images for the corresponding electrodes, Figure S37. (a)–(d) EDS spectra and corresponding atomic percentage tables of the post annealing duration variation set, Figure S38. (a) and (b) Polarization curves of HER and OER for the V-FeBP electrodes of the post annealing duration variations set. The 15-mim sample demonstrated the best HER and OER performances, Figure S39. (a)–(d) EDS spectra and corresponding atomic percentage tables of the post annealing temperature variation set followed by vanadium (V) doping (V-FeBP), Figure S40. (a)–(d) HER CV plots of post annealing temperature variation set (V-FeBP). (e) Linear plot for the anodic and cathodic current density Vs scan rate. (f) HER double layer capacitance (*C_dl_*) plots, Figure S41. (a)–(d) OER CV plots of post annealing temperature vernation set (V-FeBP). (e) Linear plot for the anodic and cathodic current density Vs scan rate. (f) OER *C_dl_* plots, Figure S42. (a)–(a-1) SEM images of Pt/C electrode. (b) EDS spectra with the atomic and weight percentage. For the Pt/C electrode fabrication, 20 mg of Pt/C and 60 µL of 5% Nafion (117 solutions, Sigma-Aldrich, St. Louis, MO, USA) were dispersed into an ethanol and DI water mixture solution (2 mL, 50:50 solution). The mixture solution was then ultrasonicated for 30 min for uniform dispersion. The bare Ni foam was then immersed in the solution for 30 min. The Pt/C electrode was dried at ambient, Figure S43. (a)–(a-1) Morphological analysis of RuO_2_ electrode. (b) EDS spectra and atomic and weight percentage. To fabricate the RuO_2_ electrode for the OER reference, 40 mg of RuO_2_ and 60 µL of 5% Nafion (117 solutions, Sigma-Aldrich, St. Louis, MO, USA) were dispersed into the 2 mL mixture of ethanol and DI water at the volume ratio 50:50. The mixture was ultrasonically mixed for 30 min. The Ni foam was then dipped in the dispersion solution for 30 min followed by being dried in the air, Figure S44. HER LSV and CA current comparison of the best electrode V-FeBP (50 °C) in 1 M KOH. (a) HER polarization curves. (b) CA response at −0.034, −0.059, −0.082 and −0.101 V. (c) Bar plot comparison of current density for LSV and CA. (d) Percentage difference, Figure S45. OER LSV and CA current comparison of the best electrode V-FeBP (50 °C) in 1 M KOH. (b) CA response at 1.42, 1.45, 1.47 and 1.49 V. (c) Bar plot comparison of current density for LSV and CA. (d) Percentage difference, Figure S46. HER and OER turnover frequency (TOF) of post annealing temperature variation set (V-FeBP). (a)–(b) Vanadium active sites at 150 mV/cm^2^. (c)–(d) Fe active sites at 150 mV/cm^2^, Figure S47. 2-E LSV and CA comparison of the V-FeBP ‖ V-FeBP in 1 M KOH. (a) HER polarization curves. (b)–(c-1) CA responses at 1.55, 1.65, 1.81, 1.93, 2.05, 2.18, 2.30, and 2.42 V. (c)–(c-1) Bar plots comparison of current density for the LSV and CA. (d) Percentage difference, Figure S48. Comparison of 2-electrode performance with the state-of-art Fe-based electrodes at the current density of 50 mA/cm^2^ in 1 M KOH. Related to Table 1 in the main text, Figure S49. Comparison of 2-electrode performance with the all state-of-art electrodes at the current density of 50 mA/cm^2^ in 1 M KOH. Related to Table S1, Figure S50. Comparison of 3-electrode performance with the all state-of-art electrodes at the current density of 10 mA/cm^2^ in 1 M KOH. (a) HER. (b) OER Related to Table S2, Table S1. Comparison of 2-electrode performance with the state-of-art transition metal-based electrodes at density of 50 mA/cm^2^ in 1 M KOH, Table S2. Comparison of 3-electrode performance with the state-of-art transition metal-based electrodes at density of 10 mA/cm^2^ in 1 M KOH. The references [66,67,68,69,70,71,72,73,74,75,76,77,78,79,80,81,82,83,84,85,86,87,88,89,90,91,92] are cited in Supplementary Materials.
