# Supplementary material for: Vanadium-Doped FeBP Microsphere Croissant for Significantly Enhanced Bi-Functional HER and OER Electrocatalyst
Source: Nanomaterials (Basel). 2022 Sep 21;12(19):3283. doi: 10.3390/nano12193283 (PMC9565602; doi:10.3390/nano12193283)
Supplement: Supplementary file 1 [file nanomaterials-12-03283-s001.zip › nanomaterials-1883165-supplementary.pdf]

nanomaterials

Supplementary Materials

Vanadium-Doped FeBP Microsphere Croissant for Significantly Enhanced Bi-Functional HER and OER Electrocatalyst

Shalmali Burse, Rakesh Kulkarni, Rutuja Mandavkar, Md Ahasan Habib, Shusen Lin, Young-Uk Chung, Jae-Hun Jeong * and Jihoon Lee *

Department of Electronic Engineering, College of Electronics and Information, Kwangwoon University, Nowon-gu, Seoul 01897, Korea
* Correspondence: myloveofjh@gmail.com (J.-H.J.); jihoonlee@kw.ac.kr (J.L.)

Fabrication Process of V-FeBP Micro Sphere Croissant (MSC)

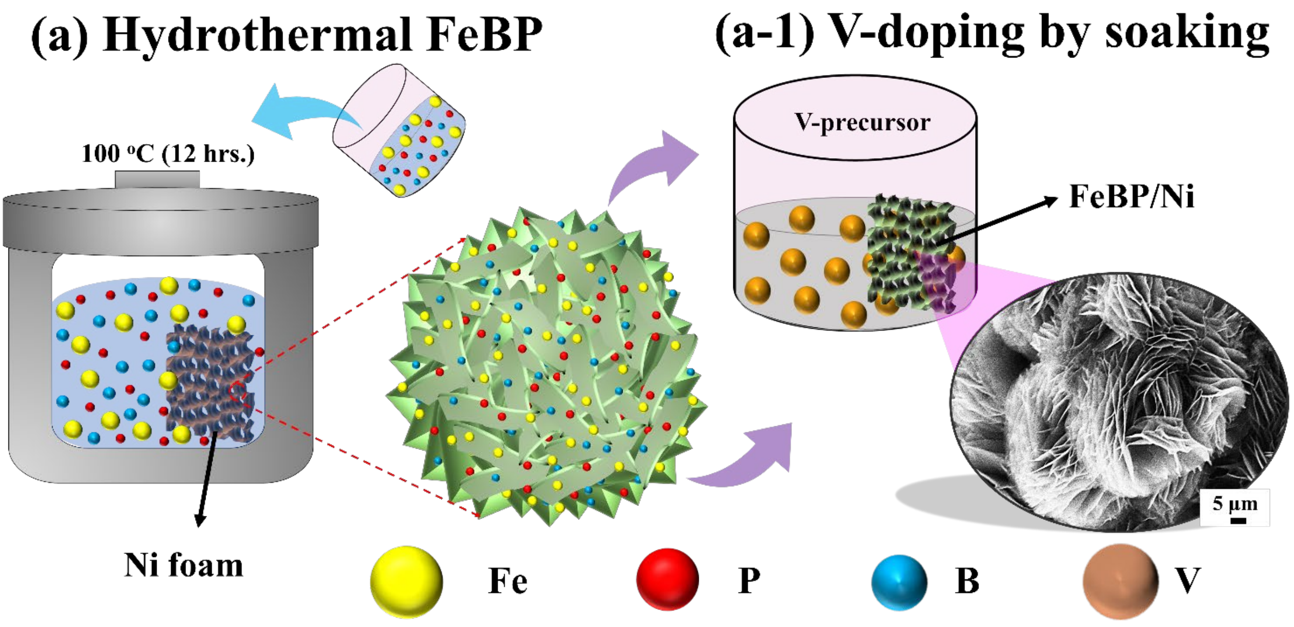

Figure S1. (**a**) Schematic representation of V-doped FeBP microsphere croissant (MSC) structure fabrication, namely V-FeBP MSC electrocatalyst. The V-FeBP MSC were fabricated by the combination of hydrothermal method and soaking approach. (**a-1**) V-doped FeBP MSC by the soaking approach.

Optimization of V-FeBP Micro Sphere Croissant (MSC) Electrocatalyst

For the fabrication of V-FeBP electrode, the FeBP electrode was firstly systematically optimized by the hydrothermal approach. For this, firstly the Fe concentration was varied between 0.1 mM to 3 mM and was fixed at 1 mM (related data: Figure 1 and Figures S5–S10). After this, the reaction temperature was optimized at 100 °C for 12 hours (related data: Figures S11–S14). This was followed by the urea concentration variation, which was fixed at 20 mM (related data: Figures S15–S16). Further, the boron and phosphate concentration ratio were optimized (related data: Figures S17–S21). The total molarity of B and P was fixed at 20 mM and the ratio was varied accordingly and fixed at $B_{30}P_{70}$. All the parameters were optimized by taking electrochemical HER and OER performance. Further,

to improve the performance of the FeBP electrode, the post-annealing was adapted to the best FeBP electrode (related data: Figures S22–S26). The post-annealing temperature and the duration were optimized at 100 °C for 30 minutes.

Furthermore, a simple soaking approach was used for V doping. A soaking method can offer simplicity and low cost. Many parameters, including soaking temperature (related data: Figures S27–S29), V concentration (related data: Figures S30–S32), and soaking duration concentration (related data: Figures S33–S35), were varied for the optimization. The optimized parameters are room temperature soaking with 0.2 mM V concentration for 15 min. Again, the V-doped FeBP electrode was treated with 2nd step annealing and optimized 2nd step annealing temperature and time ~ at 50 °C for 15 minutes (related data: Figures S36–S40). We indeed tested other approaches of doping such as single hydrothermal, dual-hydrothermal, electrochemical approaches but the simple soaking showed the best results.

Bare Ni Foam: Morphological Analysis

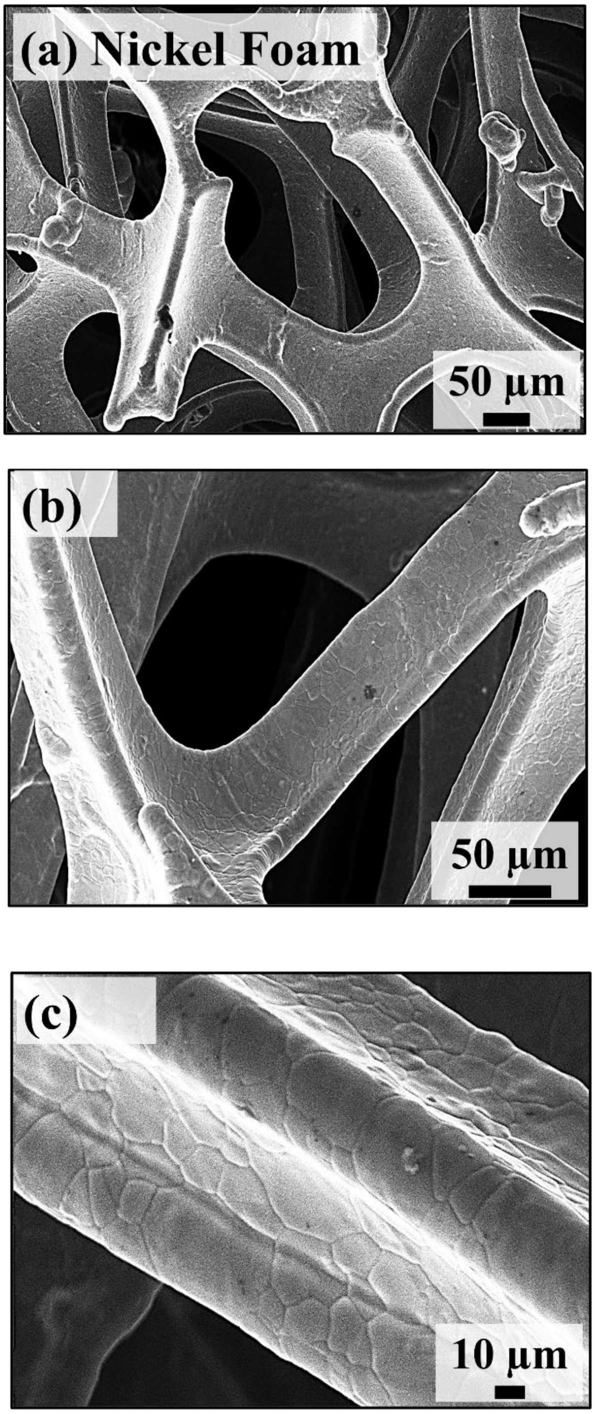

Figure S2. (**a**)–(**c**) SEM images of bare Ni foam (NF).

Bare Ni Foam: Elemental Analysis

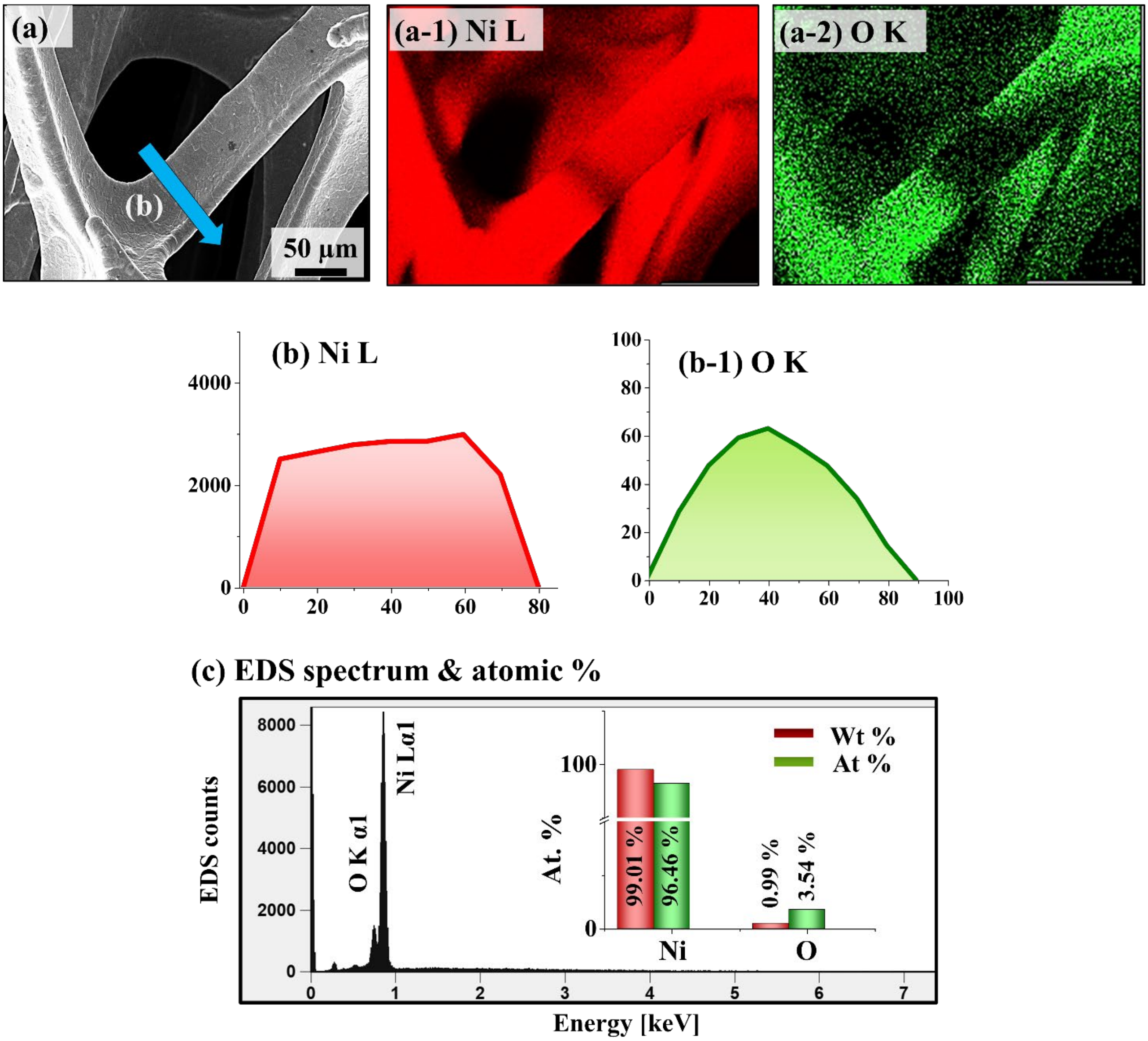

Figure S3. (**a**)–(**a-2**) EDS maps of Ni and O. (**b**)–(**b-1**) Elemental line-profiles from the arrow location in (**a**). (**c**) EDS spectrum and atomic percentage of NF.

EIS Voltage Variation of Best Sample

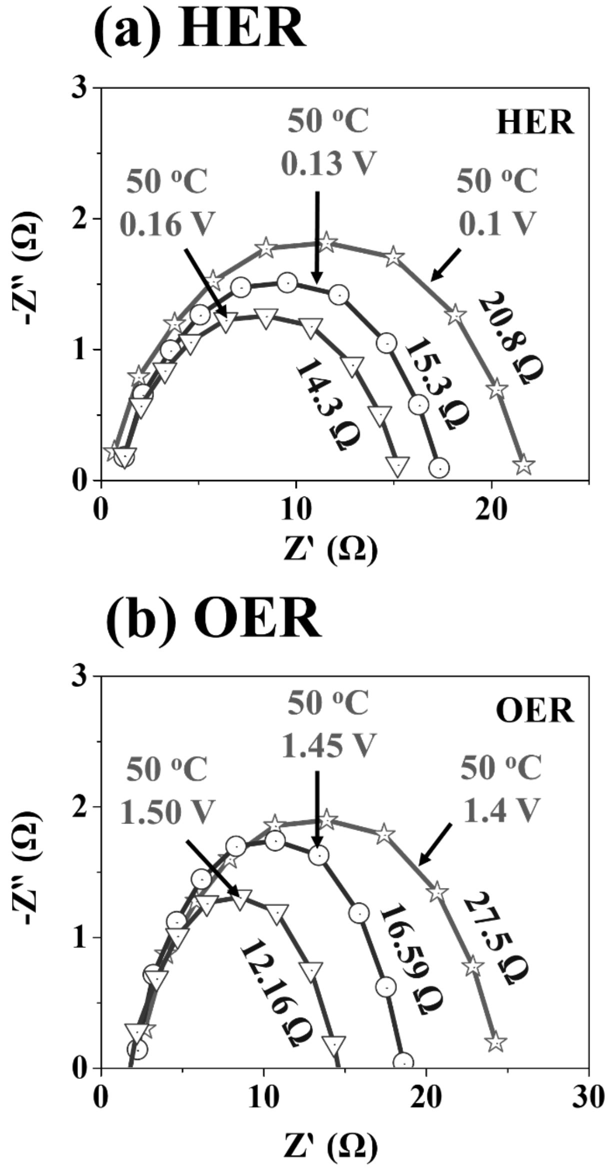

Figure S4. Electrochemical impedance spectroscopy (EIS) measured at different voltages for the best V-FeBP. (**a**) EIS for HER. (**b**) EIS for OER. The EIS measurements showed different R_{ct} values at different voltages around the turnover region [1]. The higher voltage application showed the smaller R_{ct} values and vice versa. Thus, the EIS was measured at a fixed current of 20 mA/cm^2 for the consistency between samples. The EIS was measured between 100 kHz to 0.1 Hz with an amplitude of 5 mV.

Fe Concentration Variation

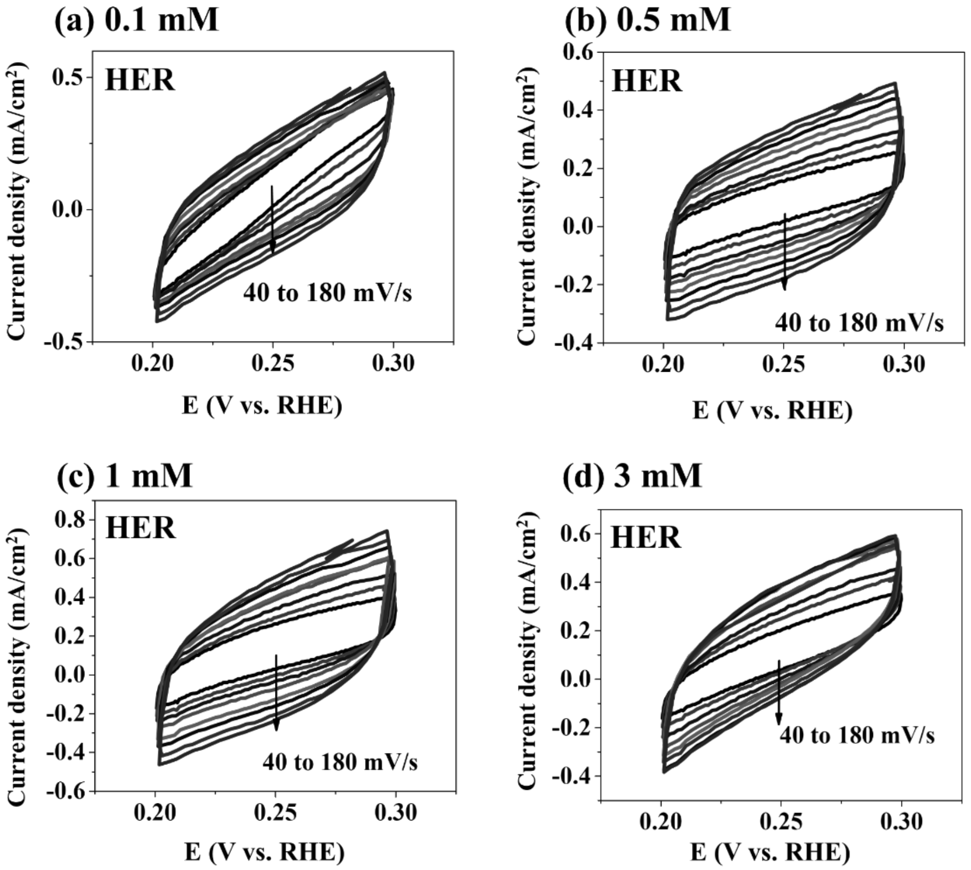

Figure S5. (**a**)–(**d**) HER CV curves of various FeBP electrocatalyst with Fe concentration variation set. The CV curves were measured in the non-faradic region where there is no charge transfer reaction occur in between 0.2 and 0.3 E below 1.023 V based on the $E_{RHE} = E + 0.059 \times pH + 0.197$ (Ag/AgCl) [2]. The actual reverse sweeping voltage (E) lies between -0.723 and -0.823 V. The scan rate was varied from 40 to 180 mV/s at the interval of 20 mV/s.

Fe Concentration Variation

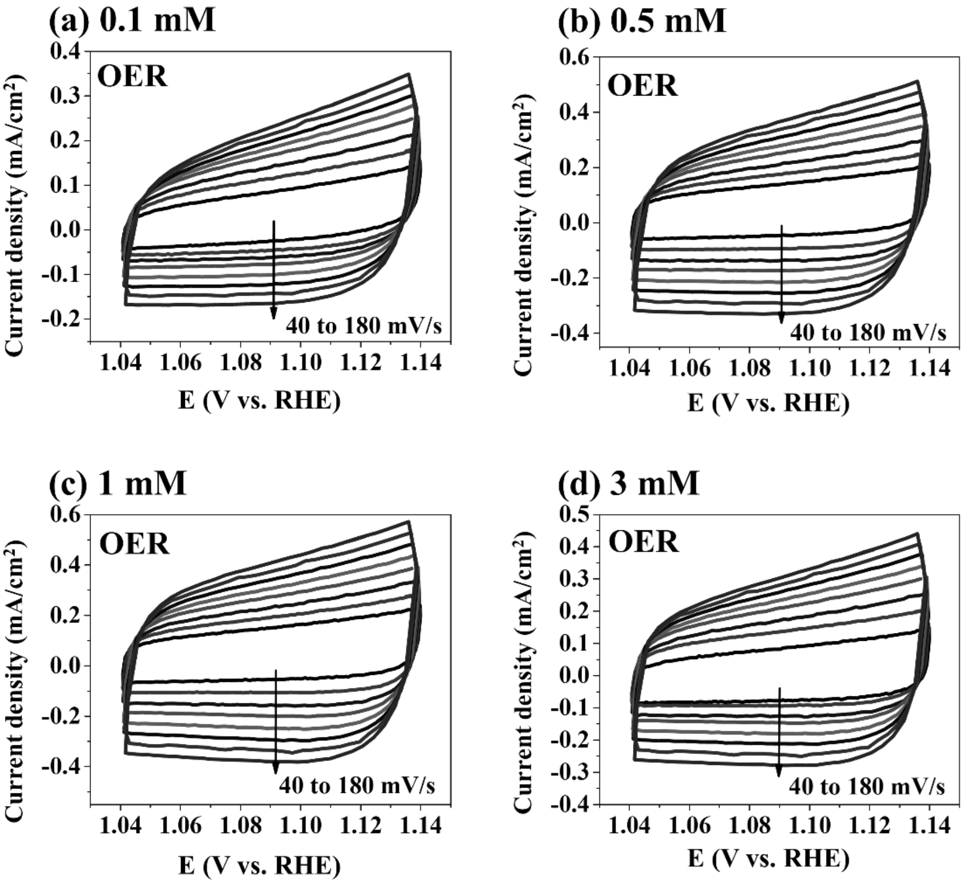

Figure S6. (**a**)–(**d**) OER CV curves of various V-FeBP catalyst with Fe concentration variation set. The CV scan rate is varied from 40 to 180 mV/s. The CV measurements were taken in a non-faradic region from 1.04 to 1.14 E below 1.23 V based on E_{RHE} = E + 0.059 × pH + 0.197 (Ag/AgCl) [3]. The actual forward sweeping voltage (E) was between 0.017 and 0.117 V.

Fe Concentration Variation

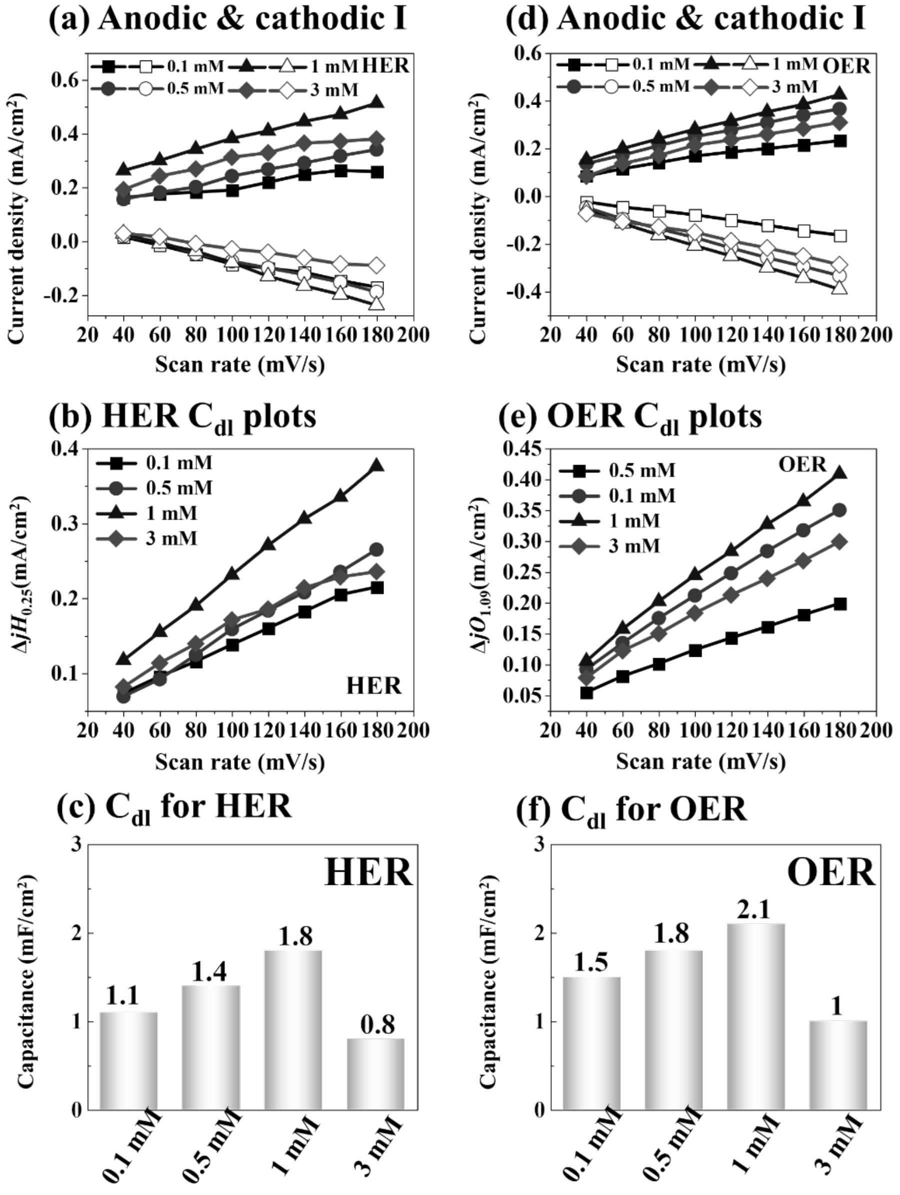

Figure S7. (**a**) and (**d**) HER and OER anodic and cathodic current density vs scan rate plots. (**b**) and (**e**) HER and OER double layer capacitance (C_{dl}) plots. The C_{dl} plots were obtained from the anodic and cathodic current density graphs: $\Delta jH_{0.25} = (Ja - Jc)/2$ and $\Delta jO_{1.09} = (Ja - Jc)/2$, where *Ja* and *Jc* is the anodic and cathodic current. (**c**) and (**f**) Bar plots of C_{dl} values. The HER and OER C_{dl} are obtained from the extracting slope of C_{dl} plots in (b) and (e). The HER and OER C_{dl} values represent the HER and OER electrochemical surface-active area (ECSA) [4].

Fe Concentration Variation

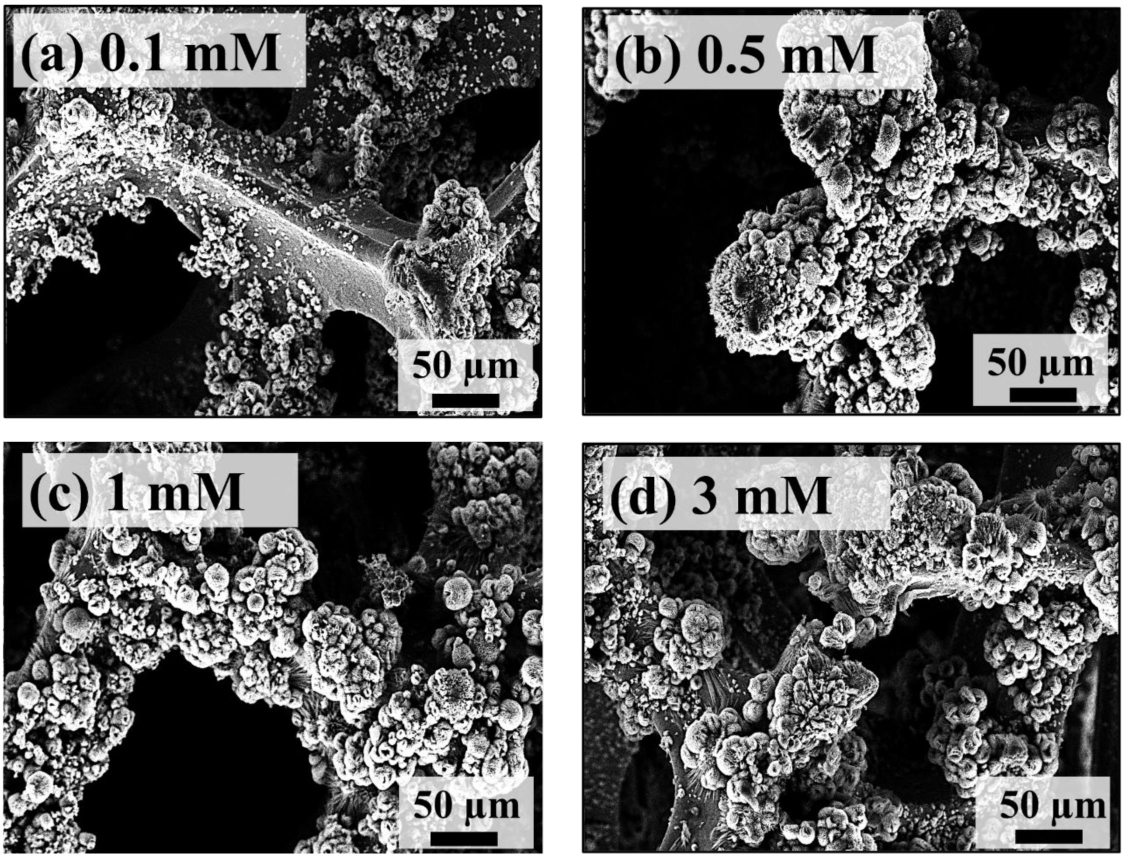

Figure S8. (**a**)–(**d**) SEM images of FeBP electrode fabricated with Fe concentration variation.

Fe Concentration Variation

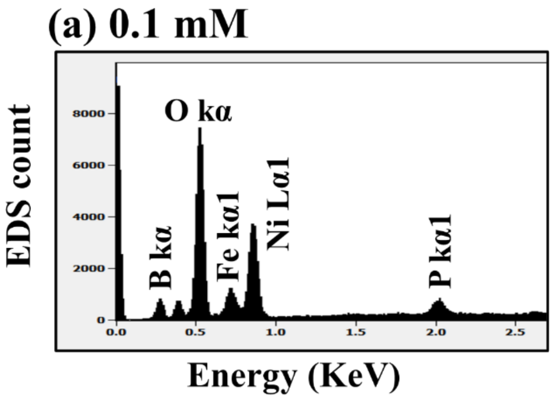

Element	Wt %	At %
B K	3.98	16.63
P K	8.82	12.86
Fe K	87.2	70.51

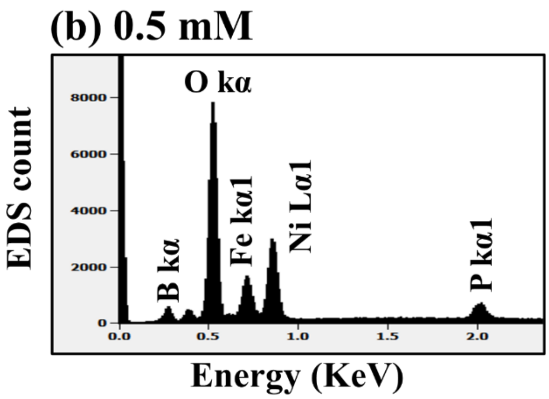

Element	Wt %	At %
B K	3.54	14.90
P K	9.92	14.58
Fe K	86.54	70.52

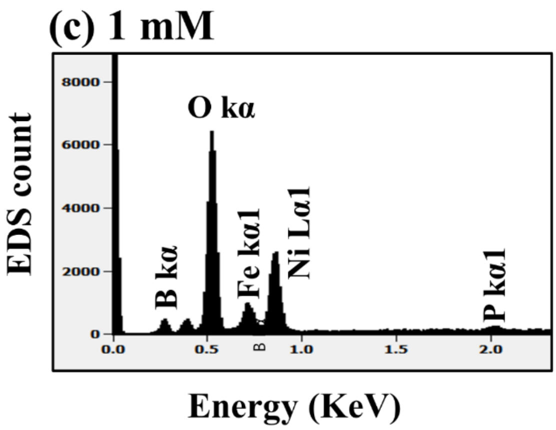

Element	Wt %	At %
B K	2.87	12.25
P K	11.26	16.78
Fe K	85.87	70.97

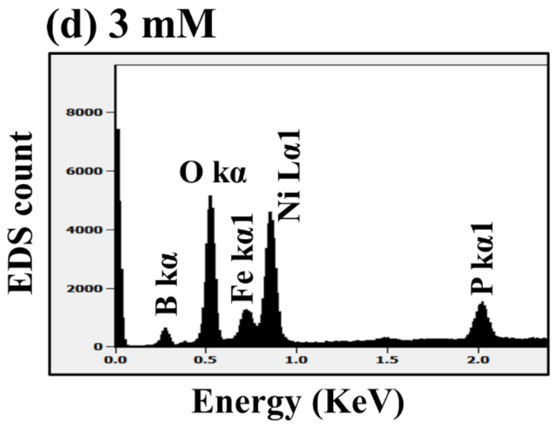

Element	Wt %	At %
B K	2.14	9.30
P K	12.46	18.89
Fe K	85.4	71.81

Figure S9. (**a**)–(**d**) EDS spectra of Fe concentration variation set with corresponding atomic percentage in the given table.

Reaction Temperature Variation

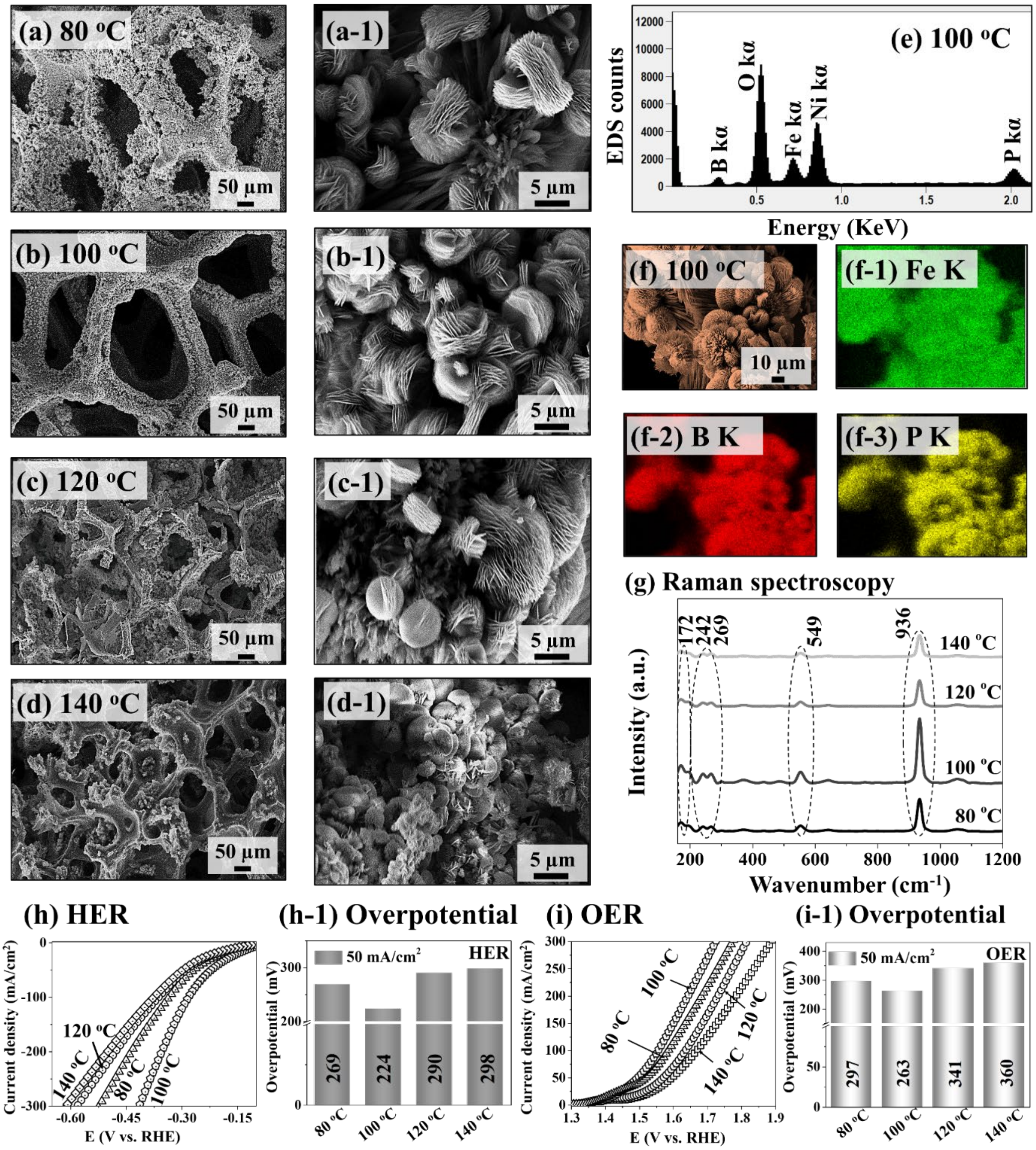

Figure S10. Reaction temperature variation for the FeBP electrocatalyst fabrication. The reaction temperature is varied between 80 and 140 °C. (**a**)–(**d**) SEM images. (**a-1**)–(**d-1**) Enlarged SEM images for the corresponding samples. (**e**) EDS spectra of 100 °C electrode. (**f**)–(**f-3**) SEM and corresponding EDS phase maps of Fe, B and P. (**g**) Raman spectra for FeBP. (**h**) and (**i**) Hydrogen evolution reaction (HER) and oxygen evolution reaction (OER) performance in 1 M KOH. (**h-1**) and (**i-1**) HER and OER overpotential values at 50 mA/cm^2.

Reaction Temperature Variation

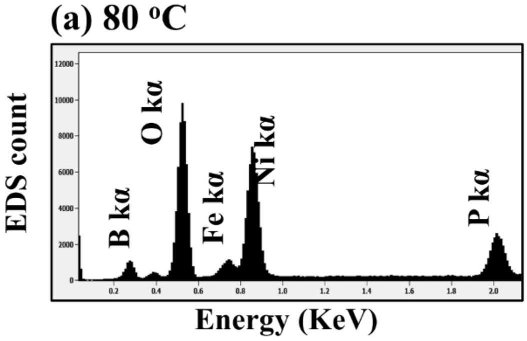

Element	Wt %	At %
B K	1.83	8.32
P K	5.56	8.82
Fe K	92.61	82.86

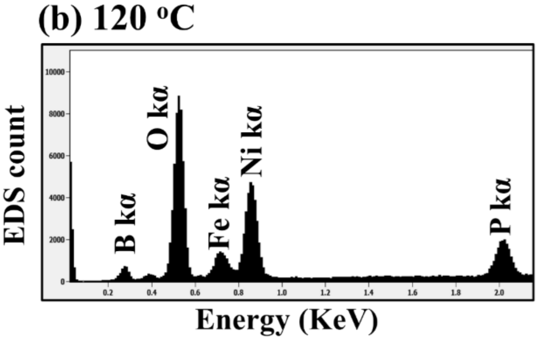

Element	Wt %	At %
B K	1.94	8.81
P K	5.20	8.24
Fe K	92.86	82.95

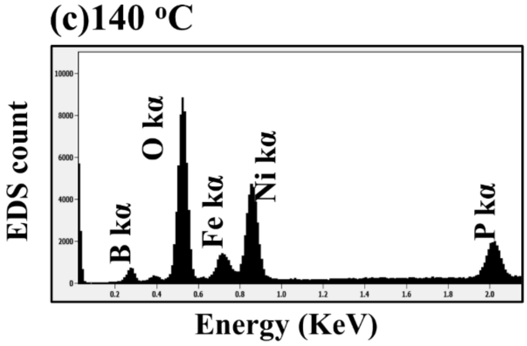

Element	Wt %	At %
B K	1.99	9.04
P K	4.85	7.69
Fe K	93.16	83.27

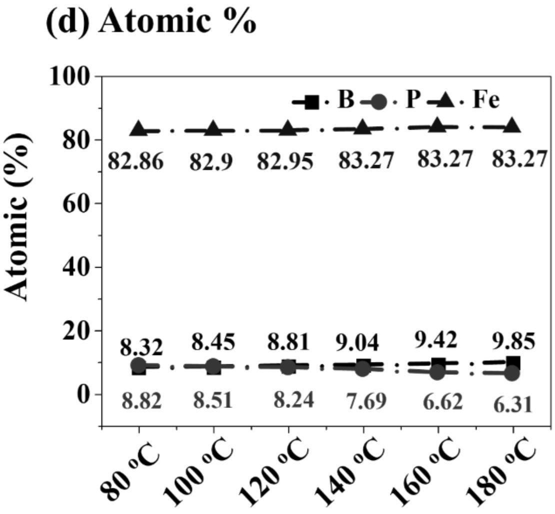

Figure S11. (**a**)–(**c**) EDS spectra of reaction temperature variation set. Tables show the atomic and weight percentage of Fe, B and P. (**d**) Atomic % of Fe, B and P.

Reaction Temperature Variation

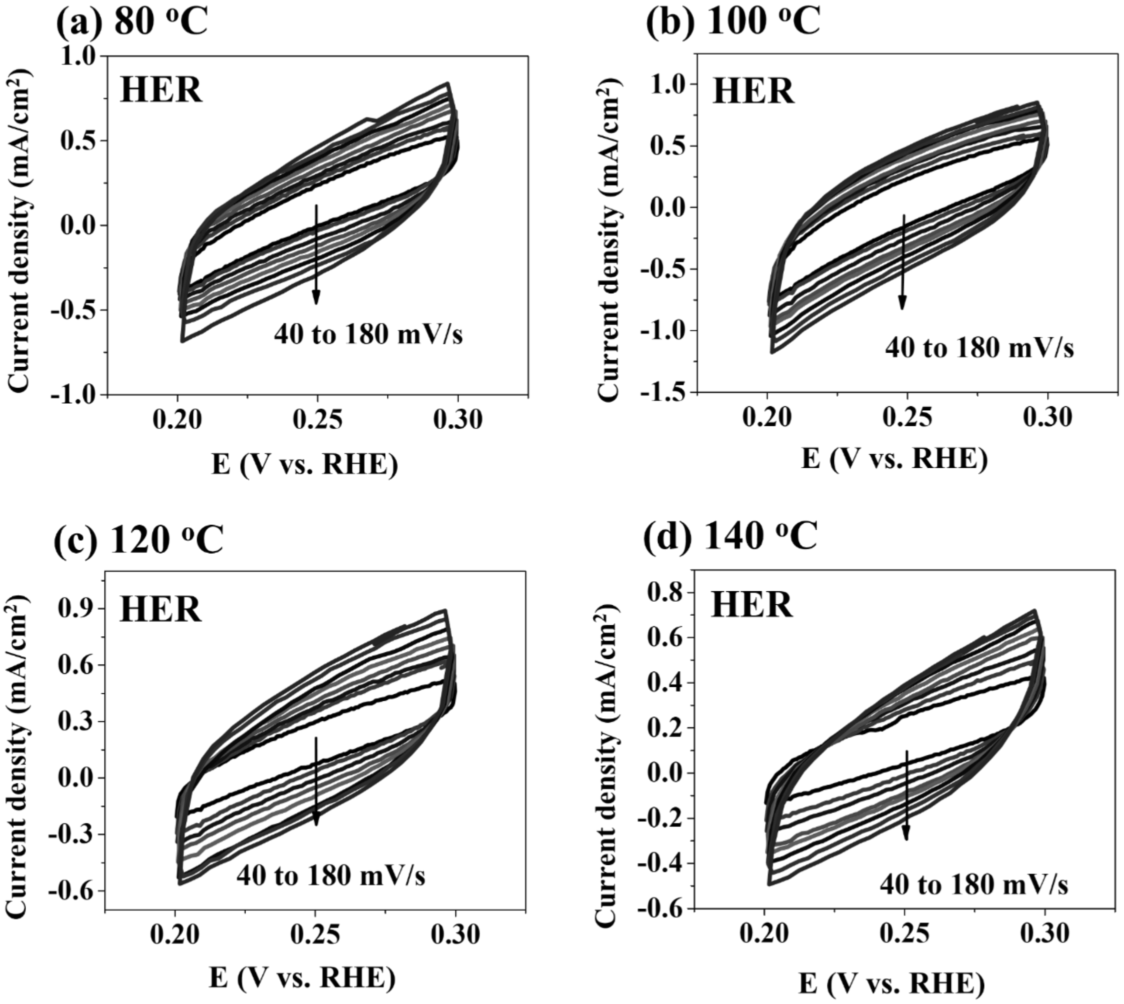

Figure S12. (**a**)–(**d**) HER CV curves of various V-FeBP electrocatlyst with reaction temperature variation set measured in the non-faradic region between 0.2 and 0.3 E. The scan rate was varied from 40 to 180 mV/s at the interval of 20 mV/s.

Reaction Temperature Variation

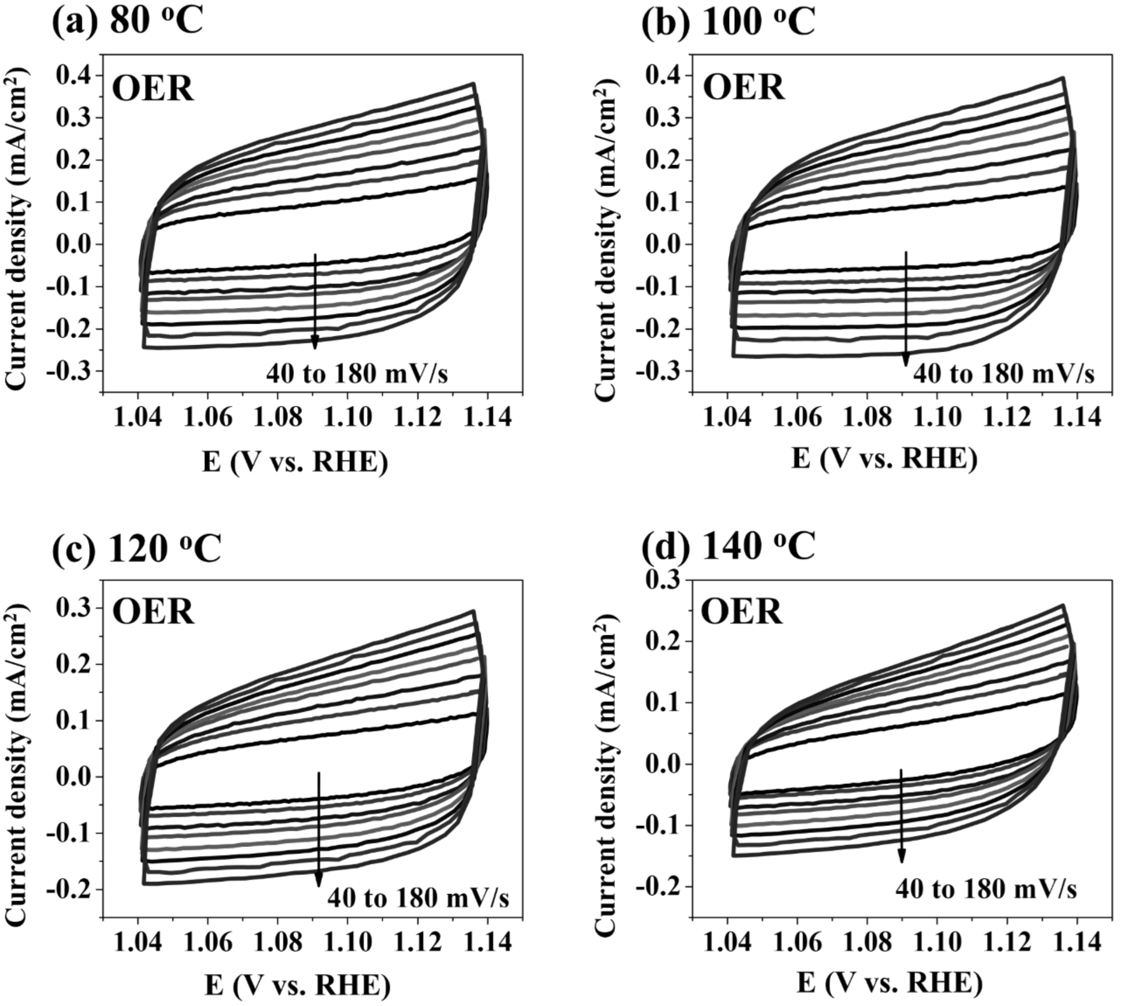

Figure S13. (**a**)–(**d**) OER CV curves of various V-FeBP catalyst with the reaction temperature variation. The CV scan rate is varied from 40 to 180 mV/s.

Reaction Temperature Variation

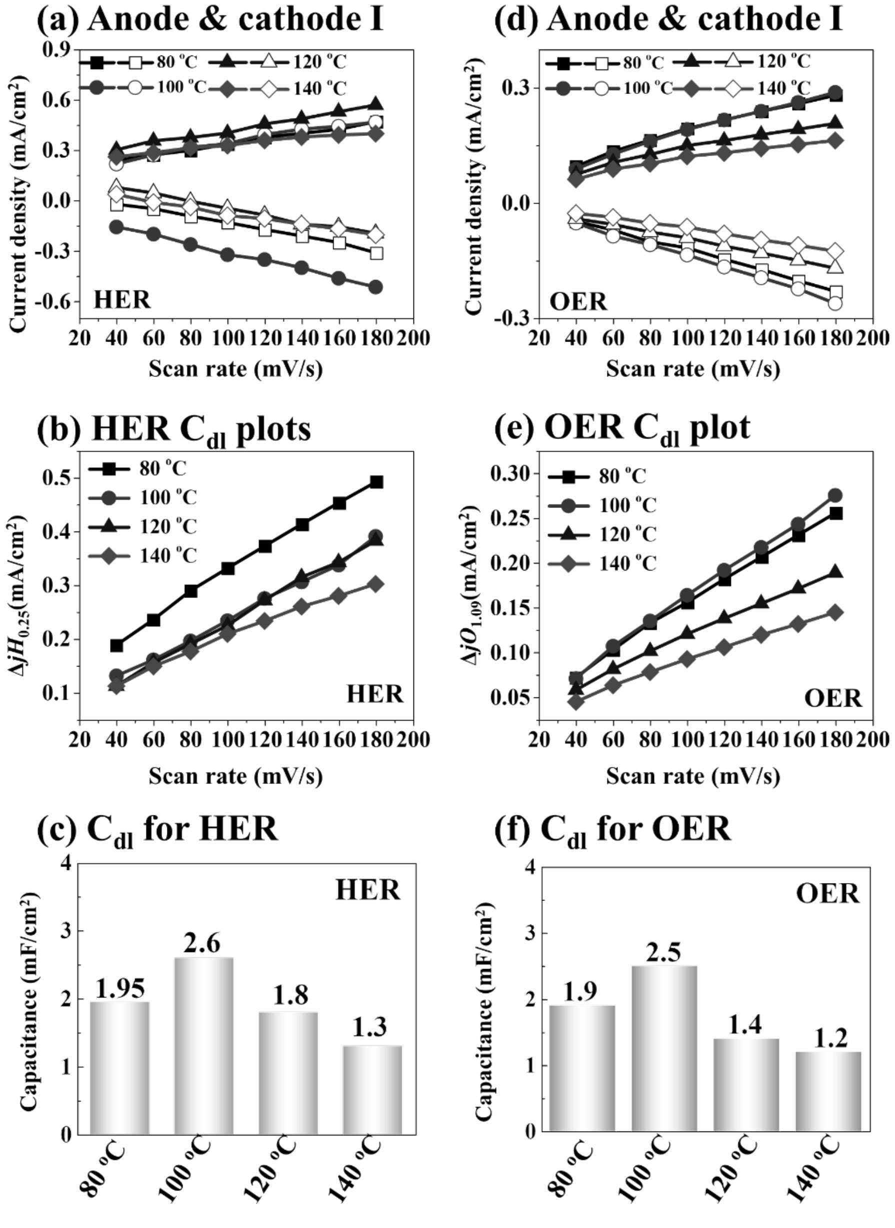

Figure S14. (**a**) and (**c**) HER and OER anodic and cathodic current density vs scan rate plots. (**b**) and (**d**) HER and OER double layer capacitance (C_{dl}) plots. (**c**) and (**f**) Bar plots showing C_{dl} values.

Urea Concentration Variation

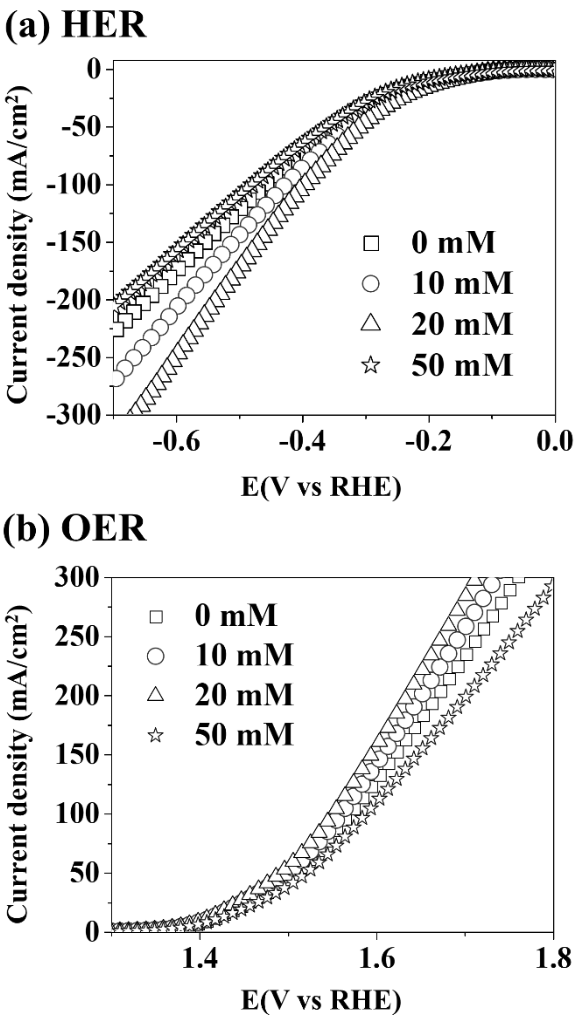

Figure S15. (**a**) and (**b**) Polarization curves of HER and OER for the FeBP electrodes with the urea concentration variation.

Boron and Phosphorus Concentration Variation

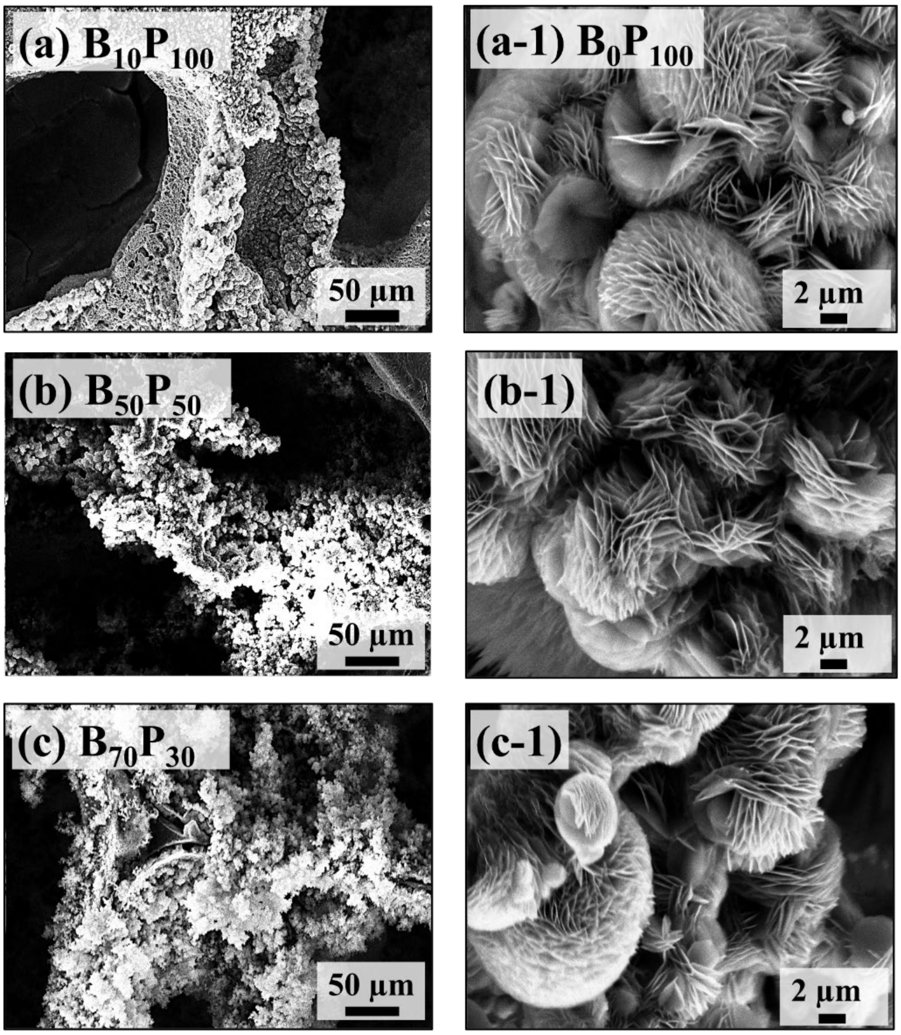

Figure S16. (**a**)–(**c**) SEM images of FeBP electrode fabricated with B and P concentration variation. (**a-1**)–(**c-1**) Enlarged SEM images for the corresponding electrodes. Other images are in the main figures.

Boron and Phosphorus Concentration Variation

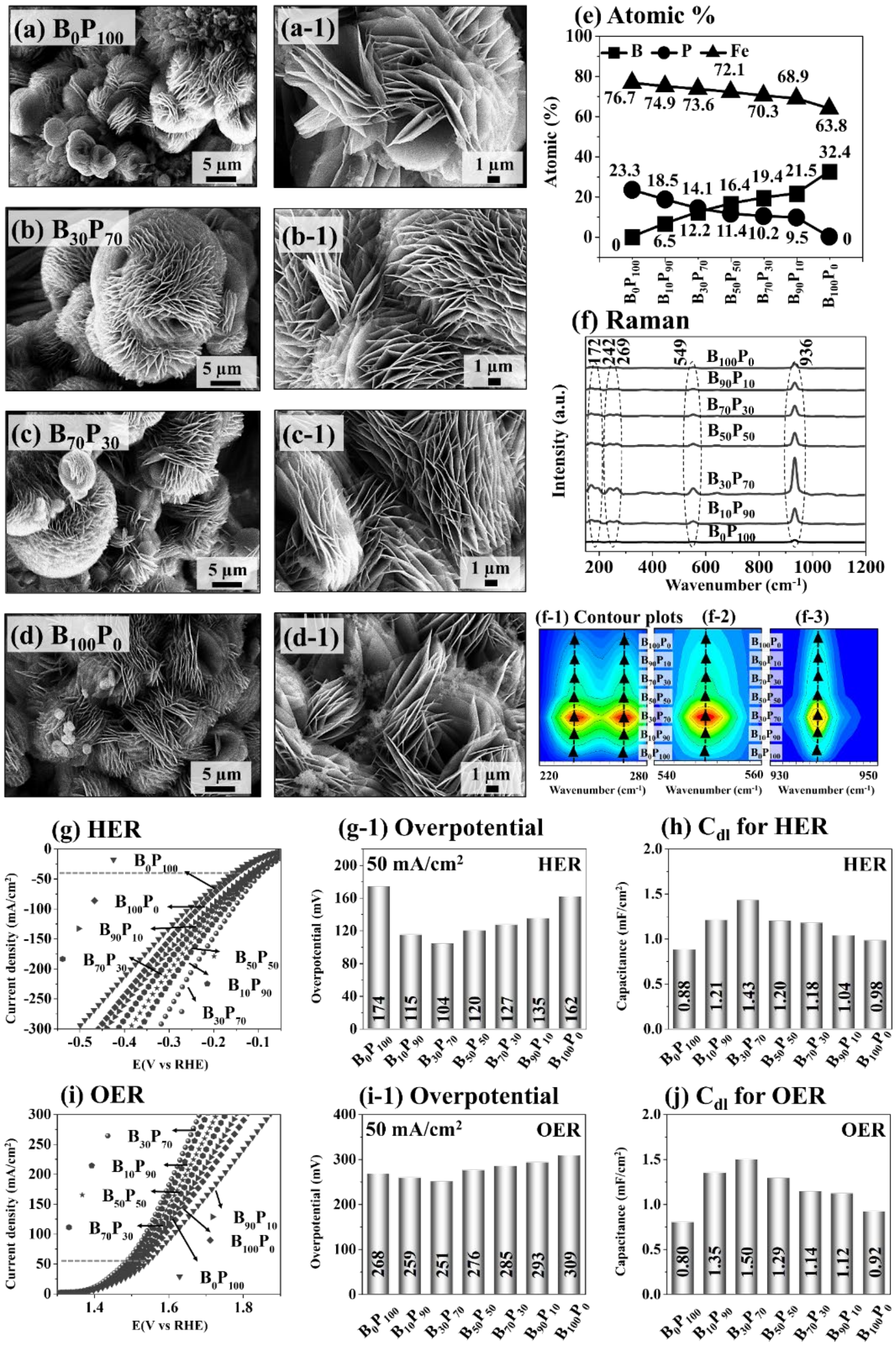

Figure S17. B and P concentration variation at 100 °C for 12 h. The total molarity of B and P was fixed at 20 mM and the ratio was varied accordingly: i.e., the $B_{30}P_{70}$ indicates 3.6 mM B and 8.4 mM of P. The Fe concentration was fixed at 1 mM. (**a**)–(**d**) SEM images for FeBP MSC electrocatalysts. (**a-1**)–(**d-1**) Enlarged SEM images. (**e**) Atomic % plot. (f) Raman spectra. (**f-1**)–(**f-3**) Contour plots of Raman peaks. (**g**) and (**i**) HER and OER LSV curves. (**g-1**) and (**i-1**) Overpotential values at 50 mA/Cm2 for HER and OER. (**h**) and (**j**) HER and OER C_{dl} values.

Figure S17 shows the B and P concertation variation for the fabrication of FeBP MSC electrocatalyst. The precursor concentration and conditions were fixed at 1 mM $FeN_3O_9{\cdot}9H_2O$ and 20 mM urea at 100 °C for 12 h reaction as optimized. The porous micro sphere croissant (MSC) was commonly found in all samples along with the increased B

concentration in Figures. S17(a)–S17(d). The size of FeBP MSCs was found to be larger for the FeBP samples as compared with the FeB and FeP. Additional SEM images with the other concentrations can be found in Figures S16. Along with the gradually increased B concentration (or decreased P concentration), the P incorporation was progressively decreased in Figure S17(e). Also, a gradual decrease in the Fe incorporation was observed with the increased B incorporation. The EDS spectra of this set can be found in Figure S17. In terms of the crystallinity, the $B_{30}P_{70}$ sample showed the best quality with the highest peak intensities as clearly seen in the Raman and contour plots in Figures S17(f)–S17(f-3). Further increase or decrease in the B concentration resulted in the reduced Raman peak intensities, indicating lower crystallinity. The electrochemical performances of this set are summarized in Figures S17(g) and S17(j). Generally, the FeP and FeB showed lower performances and the FeBP compounds showed higher performances in both HER and OER as summarized in Figures S17(g-1) and S17(i-1). The $B_{30}P_{70}$ sample showed the best performances with the lowest overpotentials of 104 mV for the HER and 251 mV for the OER at the current density of 50 mA/cm^2. In terms of the double-layer capacitance (C_{dl}), similarly the $B_{30}P_{70}$ sample demonstrated the highest values of 1.43 and 1.50 mF/cm2 for the HER and OER in Figures S17(h) and S17(j). The HER and OER CV plots can be found in Figures S20 and S21. Overall, the balanced FeBP MSC electrocatalyst demonstrated the better crystalline quality, high surface area and thus, the HER and OER electrochemical performances were superior. As mentioned, in the transition metal-based hybrid FeBP system, the balanced B and P groups can offer the high electron density on the Fe sites and thus the negative Fe atoms can effectively break water molecules and OH^- ions with the lower reaction barriers. Also, the B and P sites can effectively adsorb the negative ions such as hydroxyl ions and thus can be effective in the OER process [5].

Boron and Phosphorus Concentration Variation

(a) B_0P_{100}

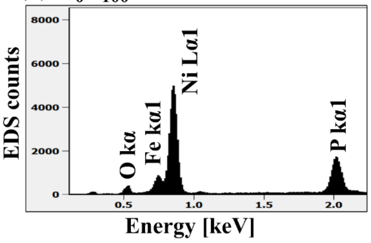

Element	Wt %	At %
B K	0.0	0.0
P K	14.42	23.30
Fe K	85.58	76.7

(b) $B_{10}P_{90}$

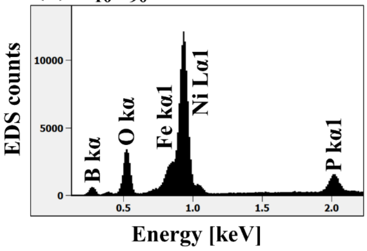

Element	Wt %	At %
B K	4.01	6.54
P K	11.24	18.52
Fe K	84.75	74.94

(c) $B_{30}P_{70}$

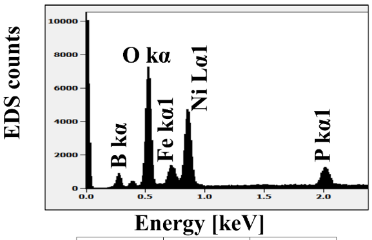

Element	Wt %	At %
B K	6.11	12.25
P K	10.23	14.11
Fe K	83.66	73.64

(d) $B_{50}P_{50}$

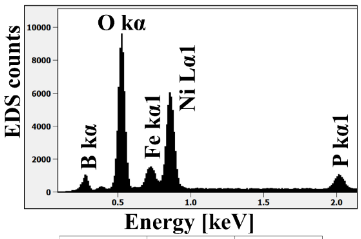

Element	Wt %	At %
B K	8.81	16.47
P K	9.18	11.43
Fe K	82.01	72.1

(e) $B_{70}P_{30}$

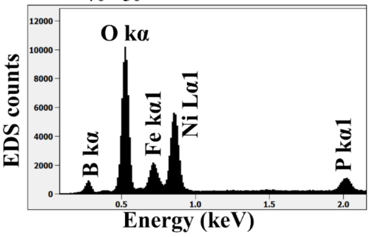

Element	Wt %	At %
B K	11.25	19.45
P K	7.45	10.24
Fe K	81.3	70.31

(f) $B_{90}P_{10}$

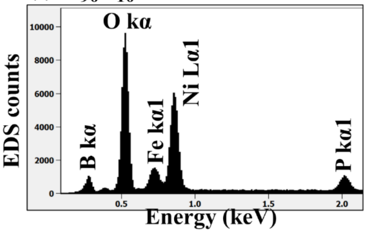

Element	Wt %	At %
B K	12.23	21.57
P K	7.23	9.52
Fe K	80.54	68.91

(g) $B_{100}P_0$

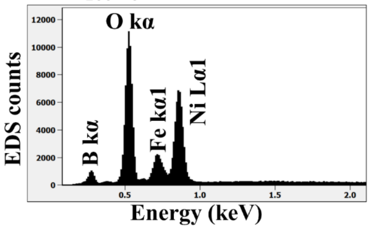

Element	Wt %	At %
B K	20.85	32.45
P K	0.0	0.0
Fe K	79.15	63.89

Figure S18. (**a**)–(**g**) EDS spectra of B and P concentration variation set as labeled and corresponding tables of the atomic and weight percentage of Fe, B and P.

XRD Analysis

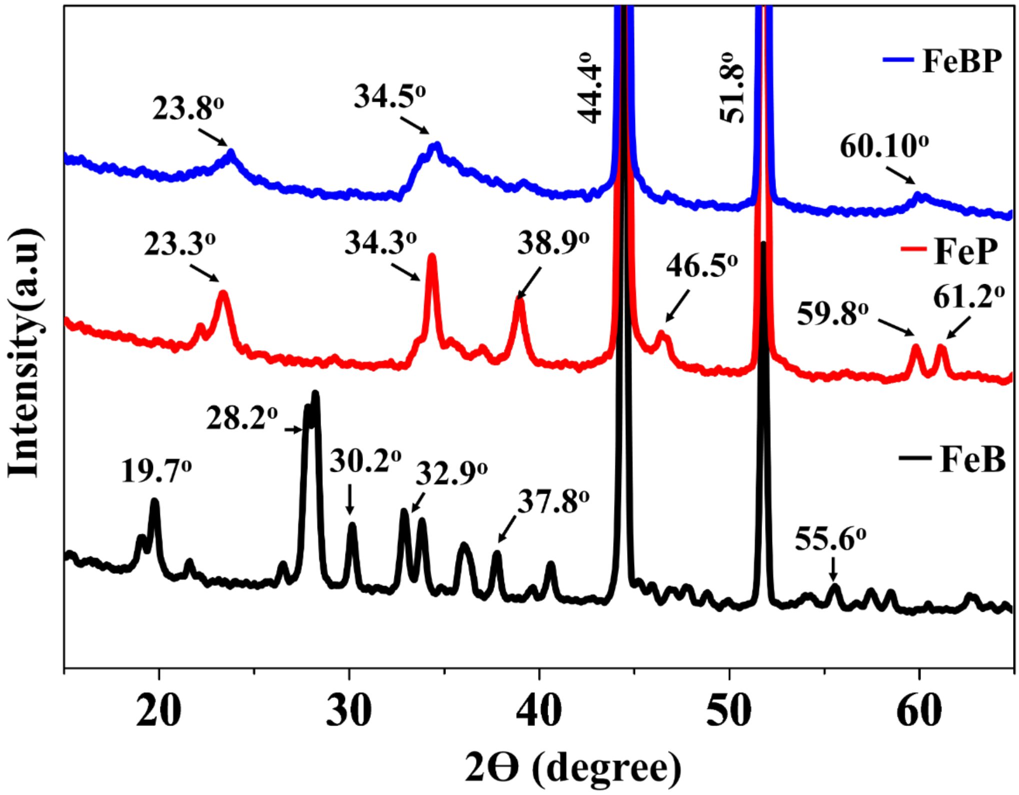

Figure S19. X-ray diffraction (XRD) analysis of FeB, FeP and FeBP. Generally, the FeP and FeB demonstrated sharper peaks and the FeBP demonstrated broad peaks. This could be due to the short-range polycrystalline phase of FeBP.

Boron and Phosphorus Concentration Variation

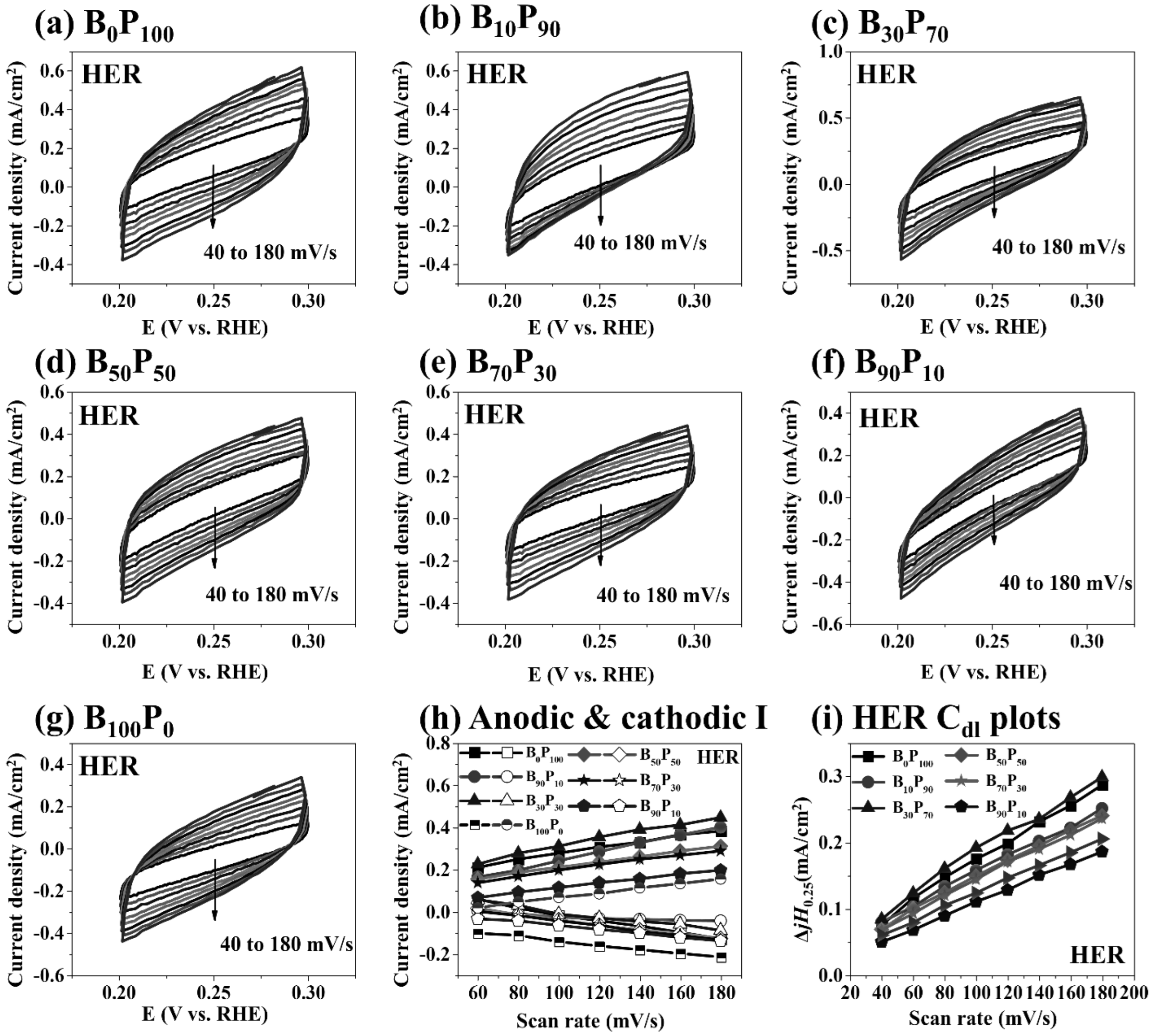

Figure S20. (**a**)–(**g**) HER CV curves of B and P concentration variation set as labeled. (**h**) Linear plots for the anodic and cathodic current density versus scan rates of the CV. (**i**) HER double layer capacitance (C_{dl}) plots.

Boron and Phosphorus Concentration Variation

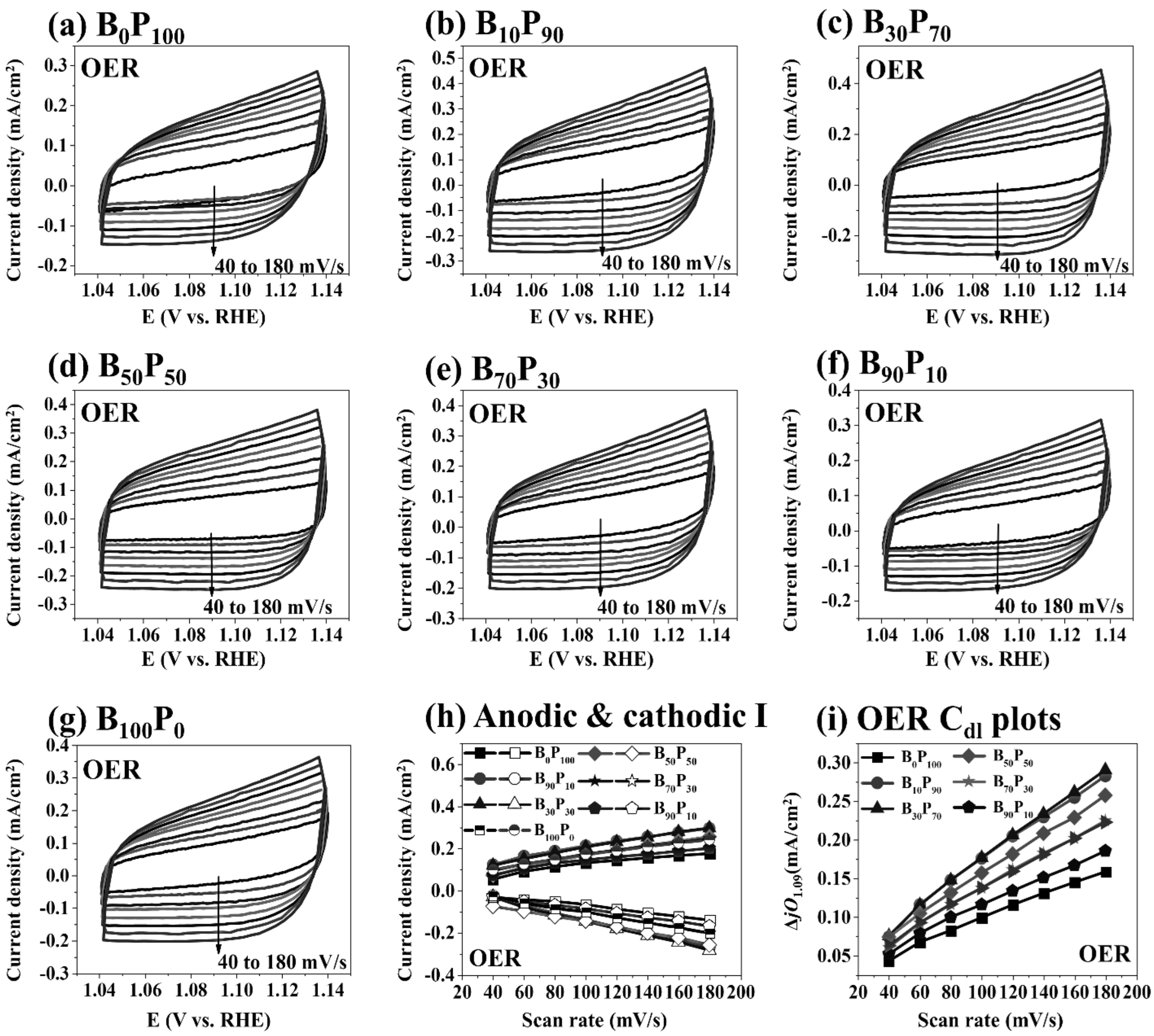

Figure S21. (**a**)–(**g**) OER CV curves of B and P concentration variation set as labeled. (**h**) Linear plots of anodic and cathodic current density versus scan rates of the CV plot. (**i**) OER double layer capacitance (C_{dl}) plots.

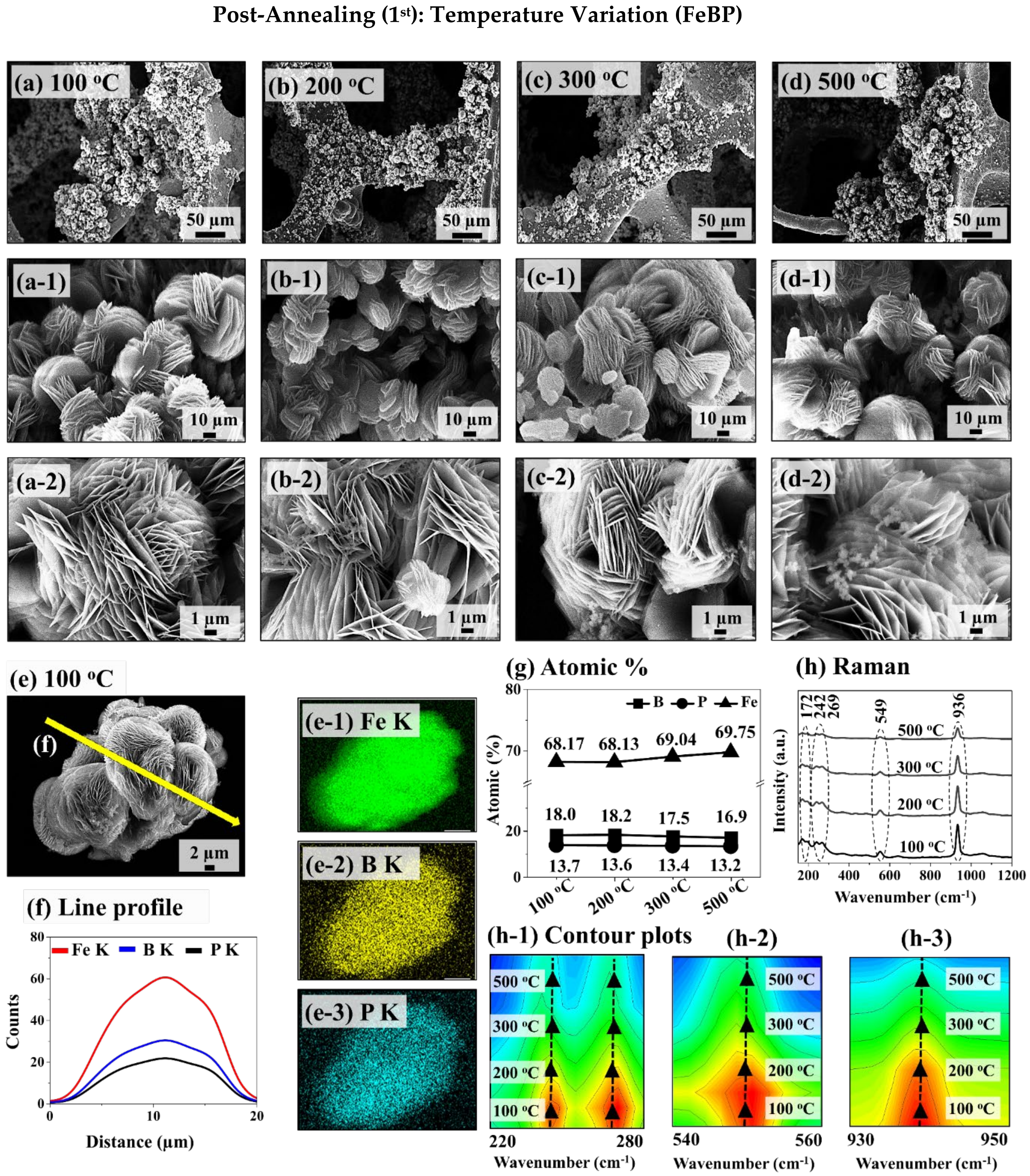

Figure S22. Post annealing temperature variation between 100 and 500 °C. The best sample (1 mM Fe, 20 mM CH_4N_2O, 3.6 mM H_3BO_3 and 8.4 mM NaH_2PO_2. H_2O) fabricated at 100 °C for 12 h was adapted for annealing. (**a**)–(**d**) SEM images. (**a-1**)–(**d-1**) and (**a-2**)–(**d-2**) Enlarged SEM images. (**e**)–(**e-3**) SEM images and its corresponding EDS maps of Fe K, B K and P K. (f) EDS line profile plot corresponds to yellow line. (**g**) Atomic percentage plot. (**h**)–(**h-3**) Raman spectra and contour plots.

Post-Annealing (1st): Temperature Variation (FeBP)

(a) 100 °C

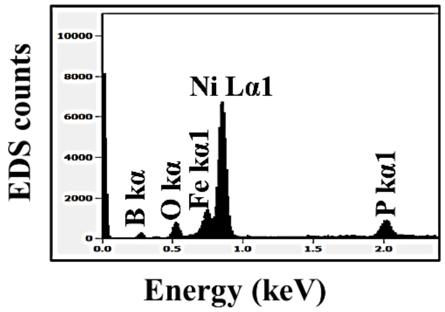

Element	Wt %	At %
B K	4.41	18.07
P K	9.63	13.77
Fe K	85.96	68.17

(b) 200 °C

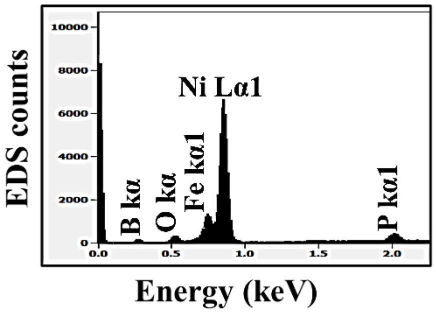

Element	Wt %	At %
B K	4.45	18.21
P K	9.56	13.66
Fe K	85.99	68.13

(c) 300 °C

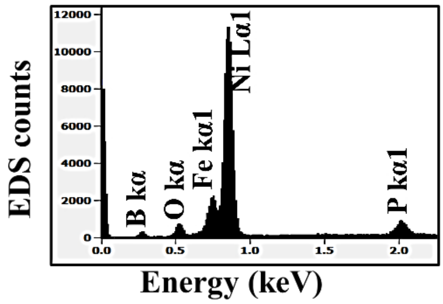

Element	Wt %	At %
B K	4.25	17.54
P K	9.32	13.42
Fe K	86.43	69.04

(d) 500 °C

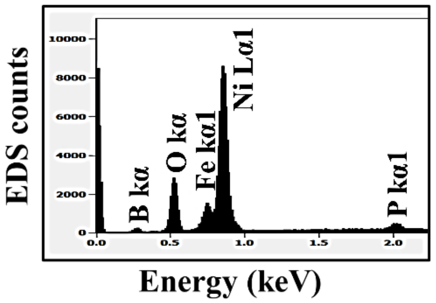

Element	Wt %	At %
B K	4.09	16.99
P K	9.15	13.26
Fe K	86.76	69.75

Figure S23. (**a**)–(**d**) EDS spectra of the post-annealing temperature variation set. Inset tables show the atomic and weighting percentages of Fe, B, and P.

Post-Annealing (1st): Temperature Variation (FeBP)

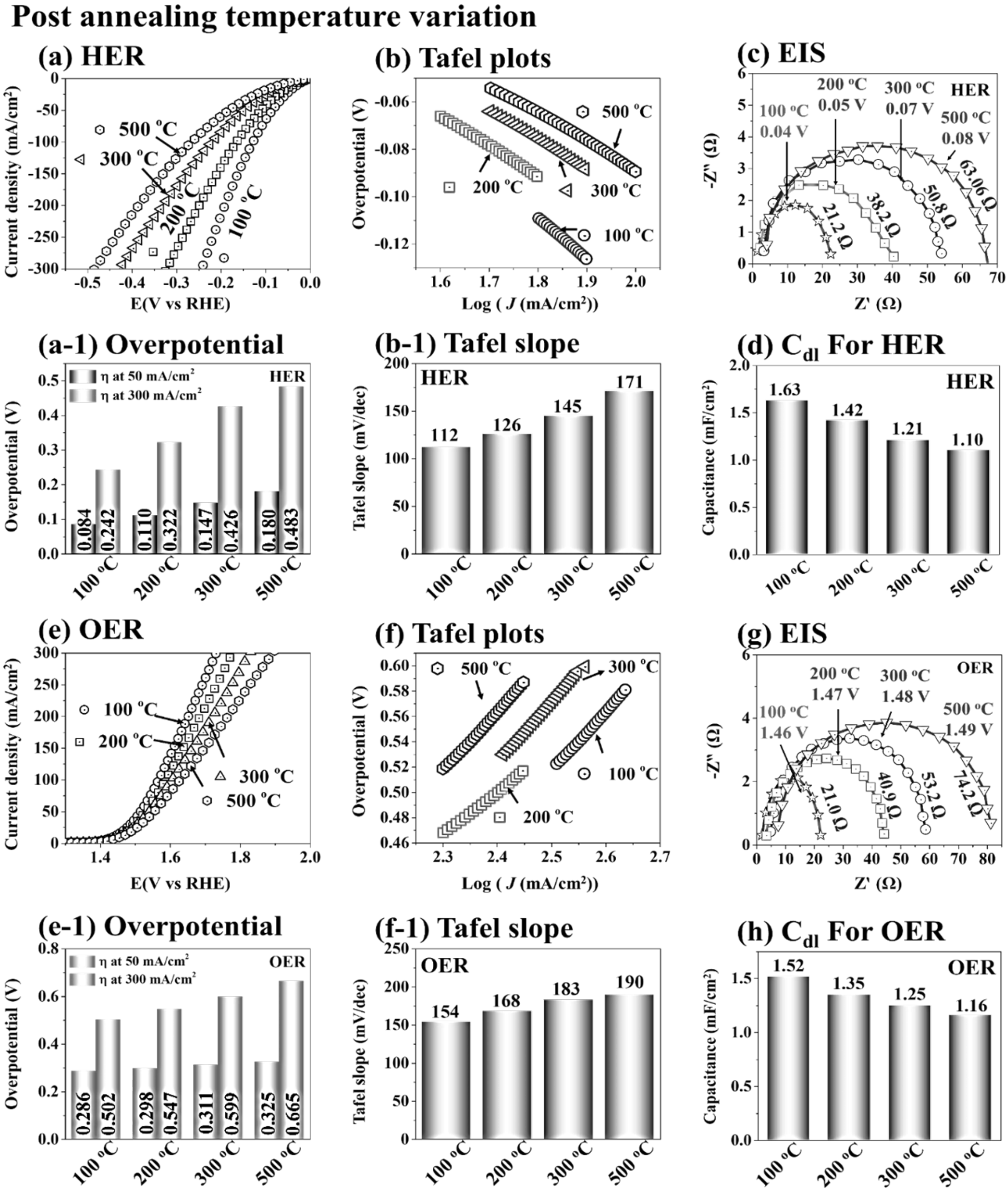

Figure S24. Electrochemical performance of post annealing temperature variation set. (**a**) and (**e**) HER and OER polarization curves of FeBP electrodes in 1 M KOH. (**a-1**) and (**e-1**) HER and OER overpotential bar graphs at 50 and 300 mA/cm². (**b**) and (**f**) Tafel slopes derived from polarization curves. (**b-1**) and (**f-1**) Tafel slope values. (**c**) and (**g**) HER and OER Nyquist plots. (**d**) and (**h**) HER and OER C_{dl} values.

Post-Annealing (1st): Temperature Variation (FeBP)

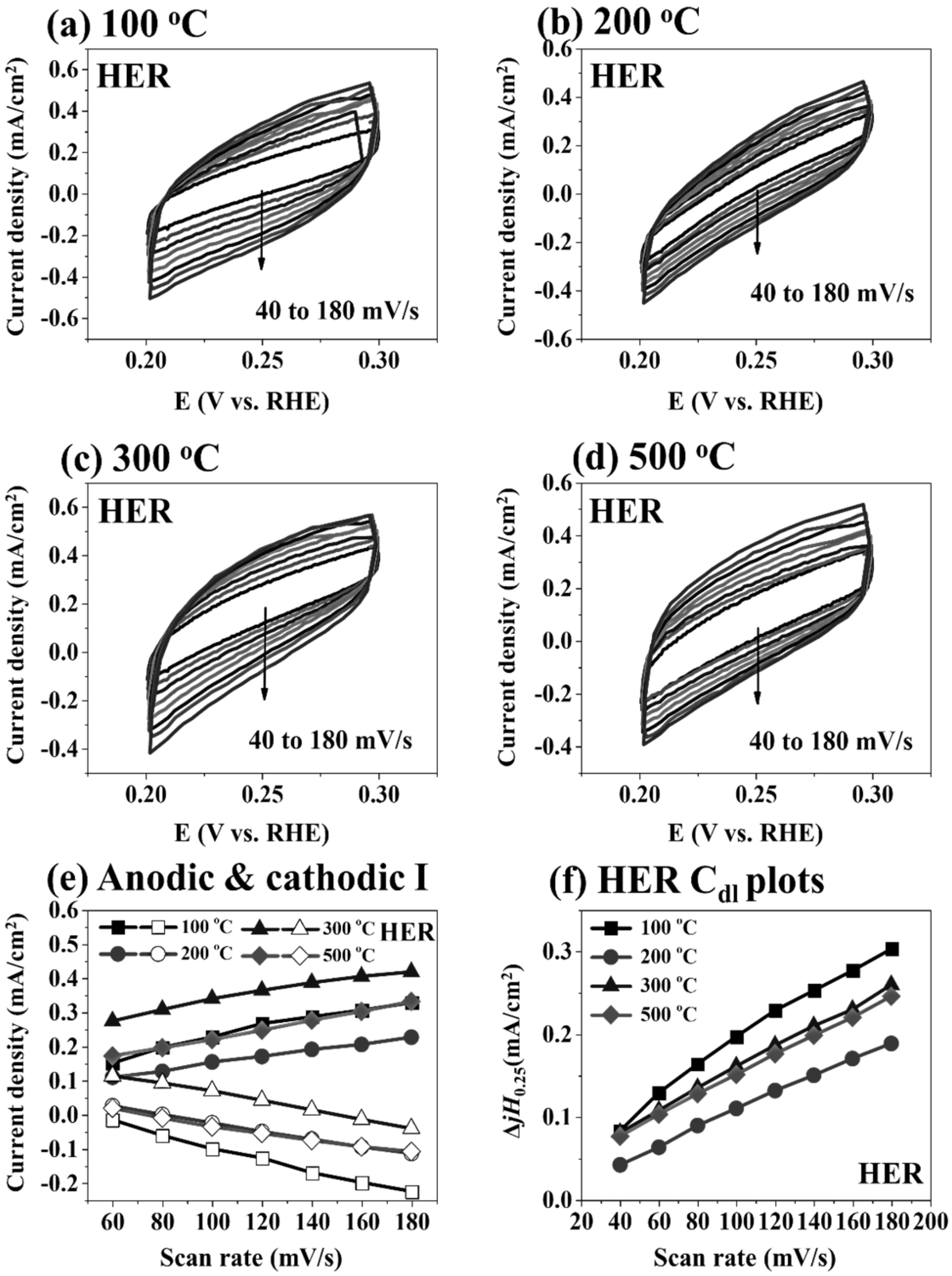

Figure S25. (**a**)–(**d**) HER CV curves of post annealing temperature variation as labeled. (**e**) Linear plots for the anodic and cathodic current density versus scan rates of the CV. (**f**) HER double layer capacitance (C_{dl}) plots.

Post-Annealing (1st): Temperature Variation (FeBP)

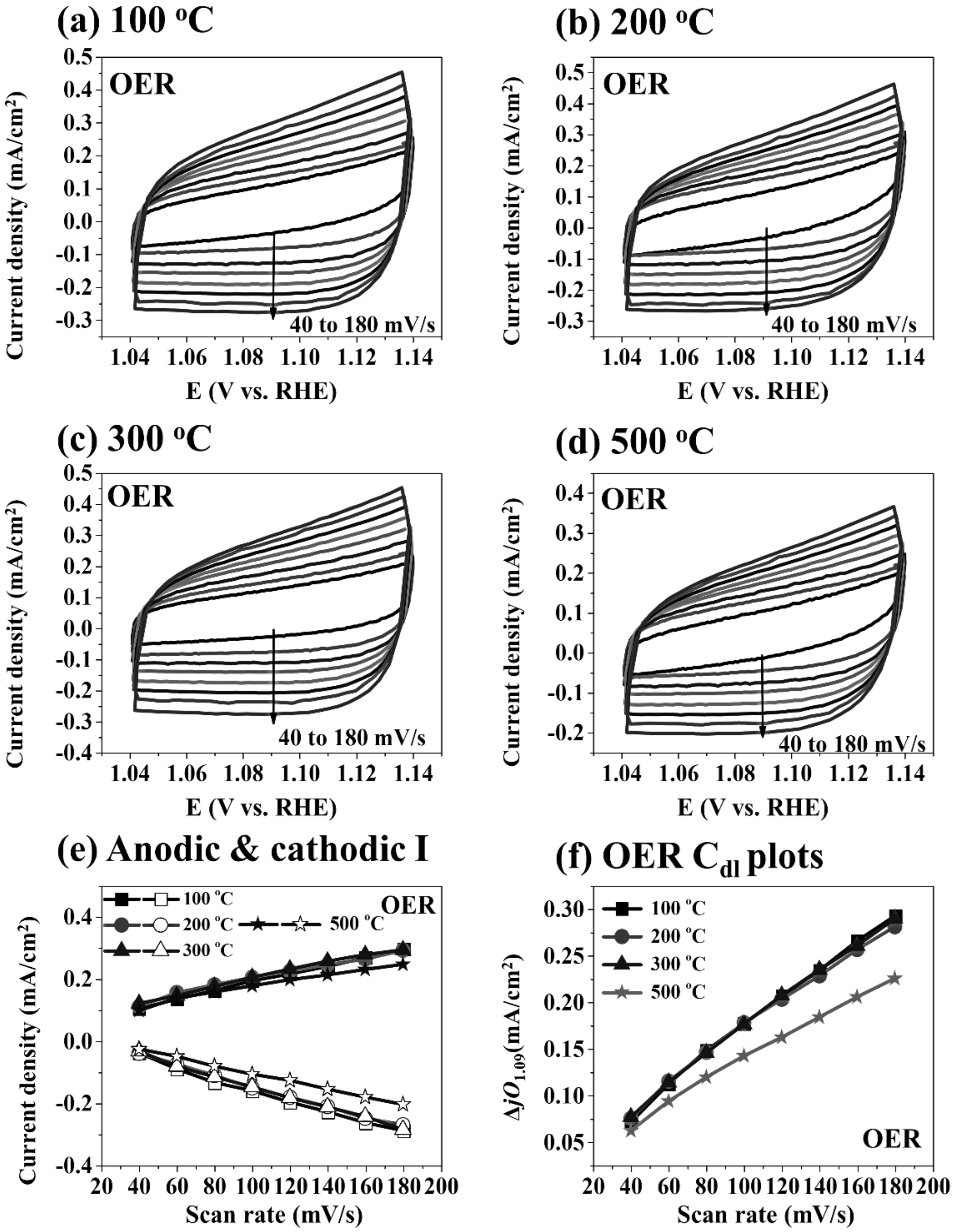

Figure S26. (**a**)–(**d**) OER CV curves of post annealing temperature variation as labeled. (**e**) Linear plots for the anodic and cathodic current density versus scan rates of the CV. (**f**) OER C_{dl} plots.

Vanadium Doping (V-FeBP): Temperature Variation

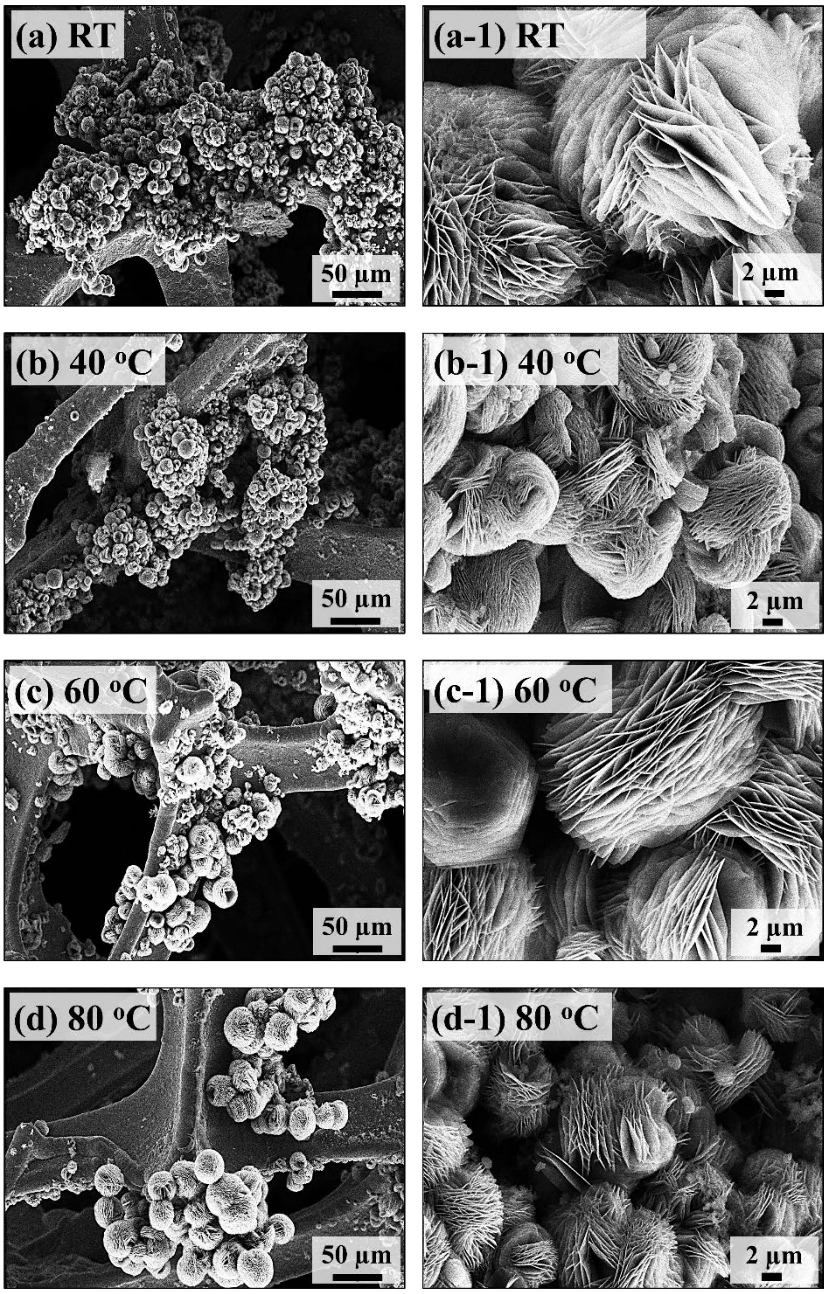

Figure S27. (**a**)–(**d**) SEM images of V-FeBP electrodes, V-doped with soaking approach at different temperature as labelled. (**a-1**) – (**d-1**) Enlarged SEM images of the corresponding electrodes.

Vanadium Doping (V-FeBP): Temperature Variation

(a) RT

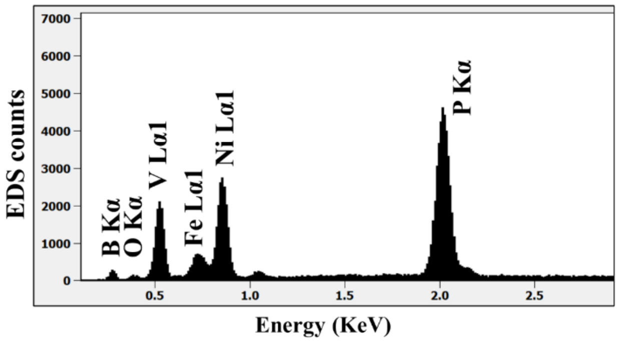

Elements	Wt %	At %
**B**	2.57	7.91
**P**	70.45	75.66
**Fe**	20.77	12.37
**V**	6.21	4.06

(b) 40 °C

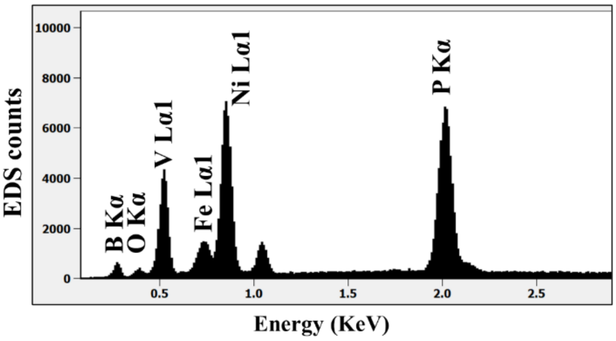

Elements	Wt %	At %
**B**	2.75	8.40
**P**	70.96	75.70
**Fe**	20.28	12.00
**V**	6.01	3.90

(b) 60 °C

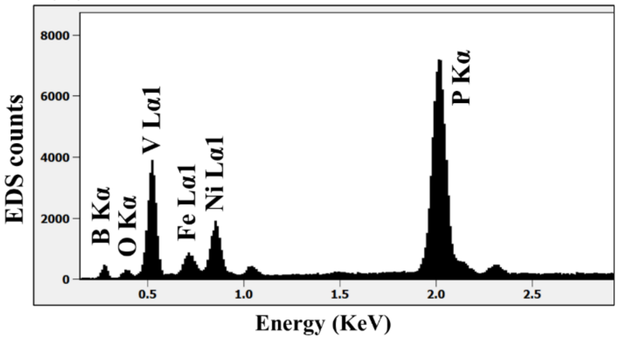

Elements	Wt %	At %
**B**	3.15	9.46
**P**	72.69	76.17
**Fe**	18.29	10.63
**V**	5.87	3.74

(b) 80 °C

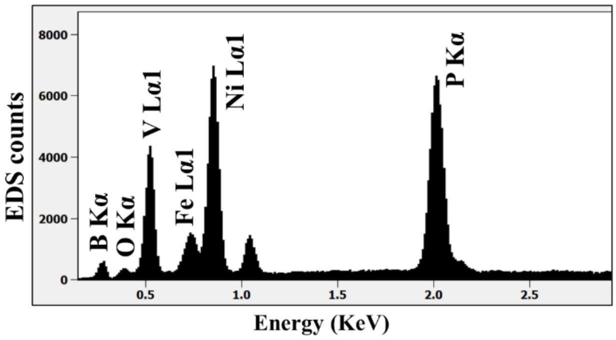

Elements	Wt %	At %
**B**	3.72	10.94
**P**	74.21	76.20
**Fe**	16.84	9.59
**V**	5.23	3.27

Figure S28. (**a**)–(**d**) EDS spectra and corresponding atomic percentage tables of the V-FeBP electrodes by the soaking temperature variation.

Vanadium Doping (V-FeBP): Temperature Variation

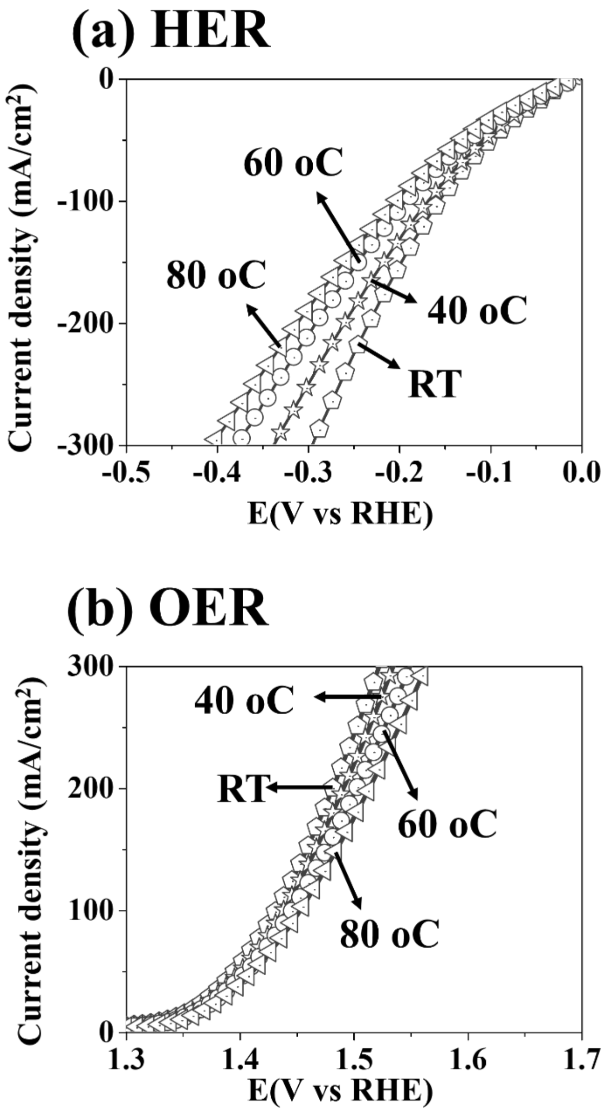

Figure S29. (**a**) and (**b**) HER and OER polarization curves of the V-FeBP electrodes by the soaking temperature variation. The room temperature (RT) sample demonstrated the best HER and OER performances.

Vanadium Doping (V-FeBP): V Concentration Variation

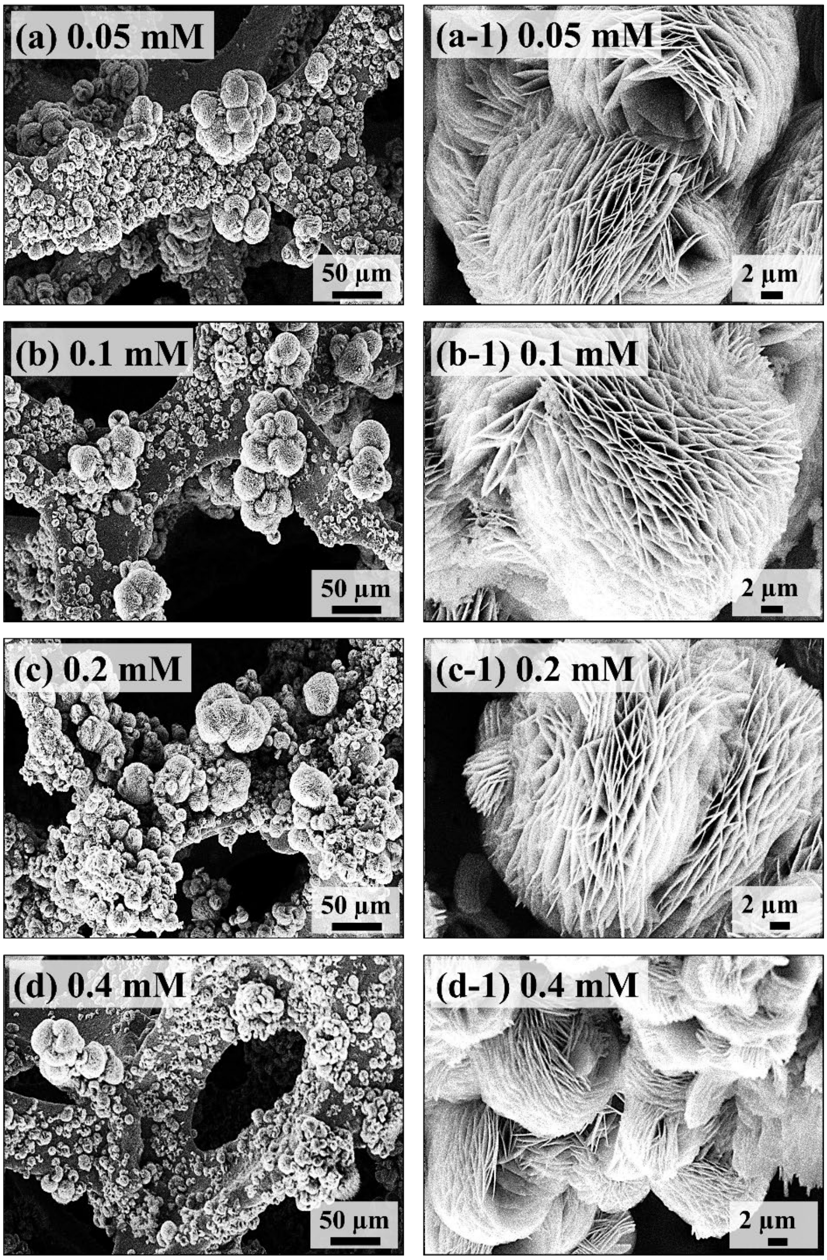

Figure S30. (**a**)–(**d**) SEM images of V-FeBP electrode fabricated with the V concentration variation. (**a-1**)–(**d-1**) Enlarged SEM images for the corresponding electrodes.

Vanadium Doping (V-FeBP): V Concentration Variation

(a) 0.05 mM

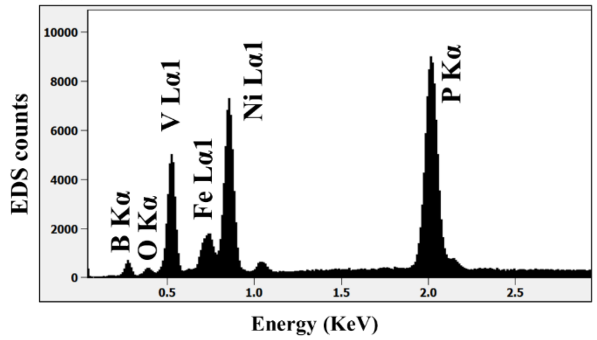

Elements	Wt %	At %
B	2.86	8.55
P	74.92	78.22
Fe	15.68	9.08
V	6.54	4.15

(b) 0.1 mM

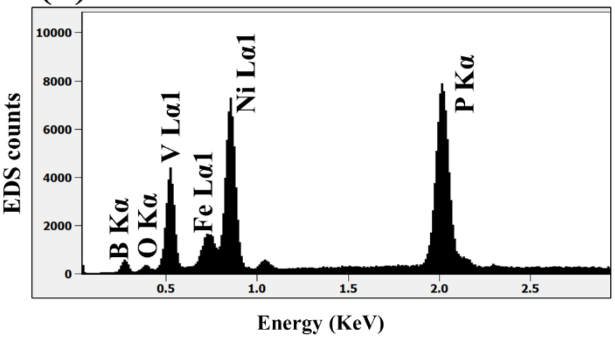

Elements	Wt %	At %
B	3.12	9.48
P	70.09	74.30
Fe	18.54	10.90
V	8.25	5.32

(c) 0.2 mM

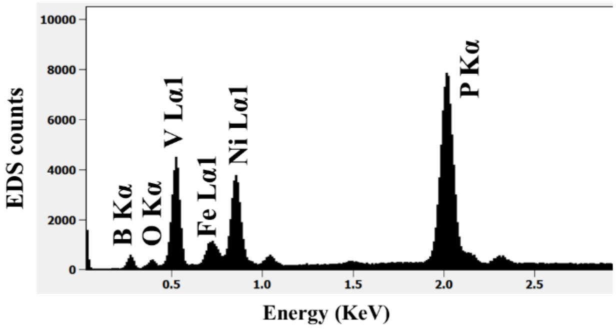

Elements	Wt %	At %
B	3.56	10.58
P	67.02	71.28
Fe	20.21	11.92
V	9.21	5.96

(d) 0.4 mM

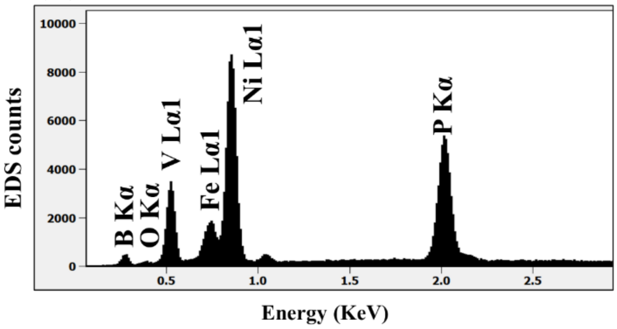

Elements	Wt %	At %
B	3.98	12.23
P	62.95	67.49
Fe	22.23	13.22
V	10.84	7.06

Figure S31. (**a**)–(**d**) EDS spectra and corresponding atomic percentage tables of the V concertation variation set.

Vanadium Doping (V-FeBP): V Concentration Variation

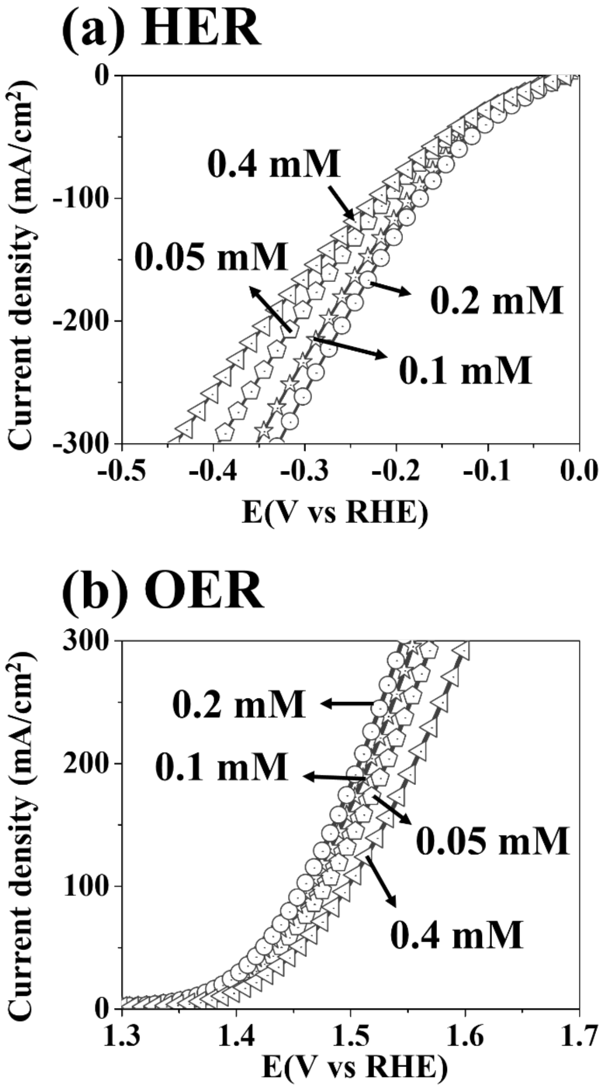

Figure S32. (**a**) and (**b**) HER and OER LSV curves of the V-FeBP electrodes with the V concentration variation. The 0.2 mM sample demonstrated the best HER and OER performances.

Vanadium Doping (V-FeBP): Soaking Time Variation

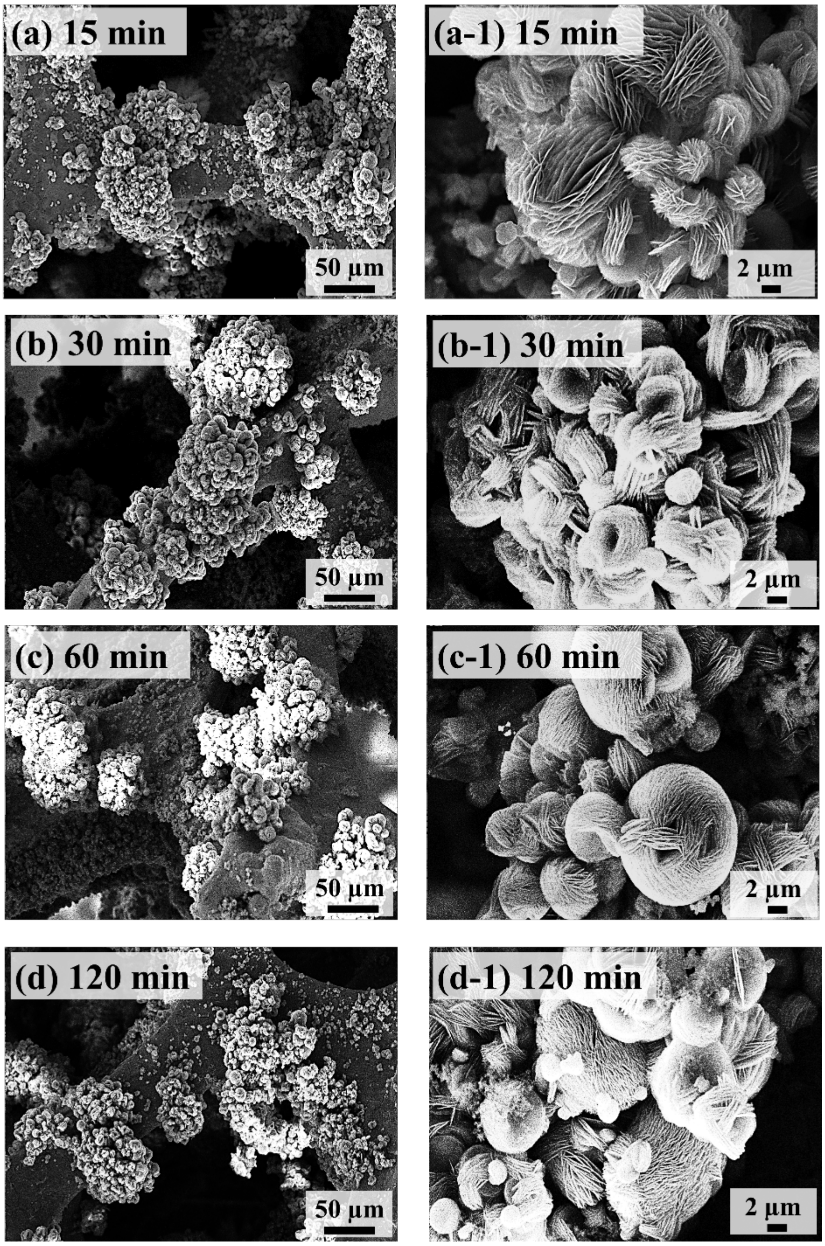

Figure S33. (**a**)–(**d**) SEM images of V-FeBP electrodes fabricated with the soaking duration variation. (**a-1**)–(**d-1**) Enlarged SEM images for the corresponding electrodes.

Vanadium Doping (V-FeBP): Soaking Time Variation

(a) 15 min

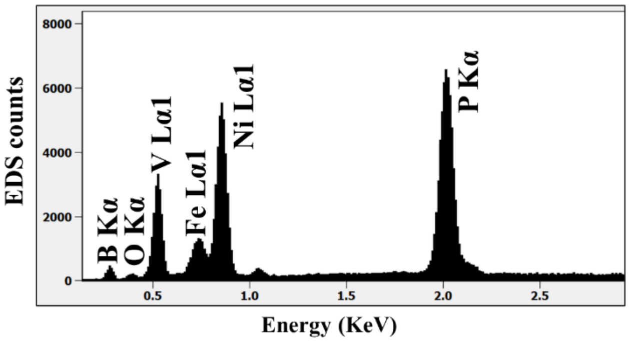

Elements	Wt %	At %
**B**	2.36	7.23
**P**	72.58	77.60
**Fe**	19.72	11.69
**V**	5.34	3.47

(b) 30 min

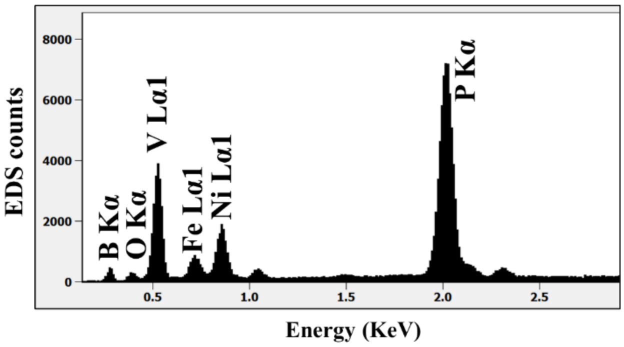

Elements	Wt %	At %
**B**	3.21	9.68
**P**	71.25	75.02
**Fe**	18.7	10.92
**V**	6.84	4.38

(c) 60 min

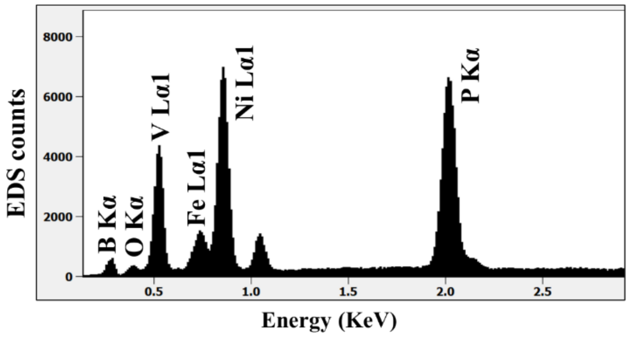

Elements	Wt %	At %
**B**	3.65	10.96
**P**	69.74	73.09
**Fe**	18.1	10.52
**V**	8.51	5.42

(d) 120 min

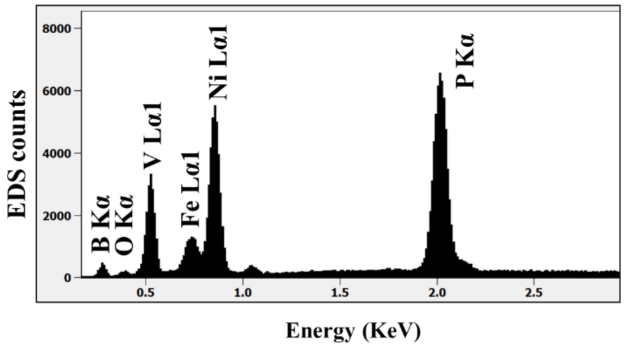

Elements	Wt %	At %
**B**	4.98	14.76
**P**	65.41	67.66
**Fe**	18.77	10.77
**V**	10.84	6.82

Figure S34. (**a**)–(**d**) EDS spectra and corresponding atomic percentage tables of the soaking time variation set.

Vanadium Doping (V-FeBP): Soaking Time Variation

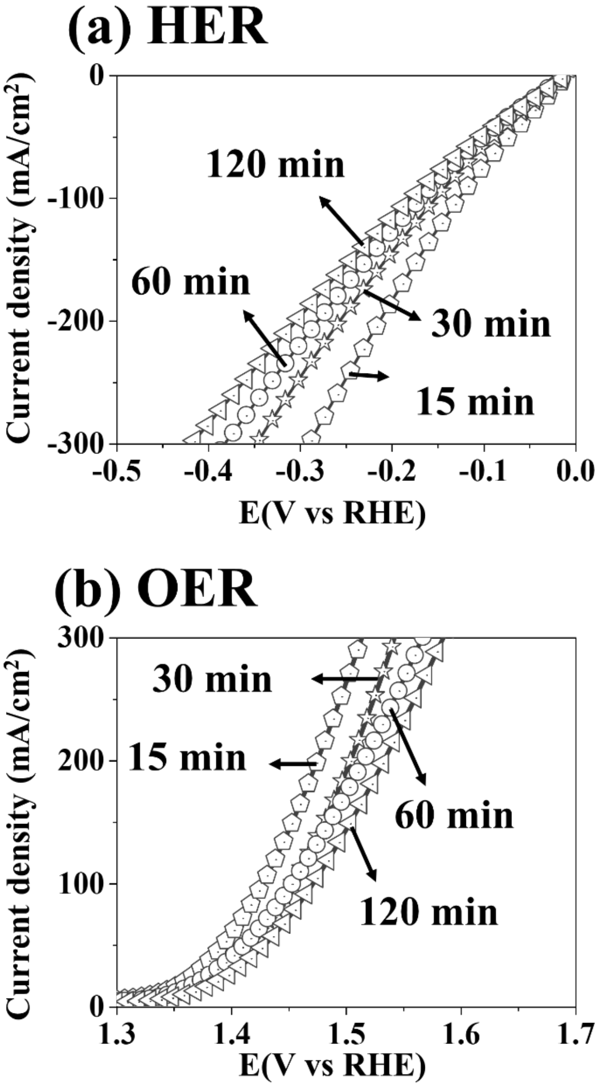

Figure S35. (**a**) and (**b**) HER and OER polarization curves of the V-FeBP electrodes with the soaking time variation. The 25-min sample demonstrated the best HER and OER performances.

Post-Annealing (2nd): Duration Variation (V-Doped FeBP)

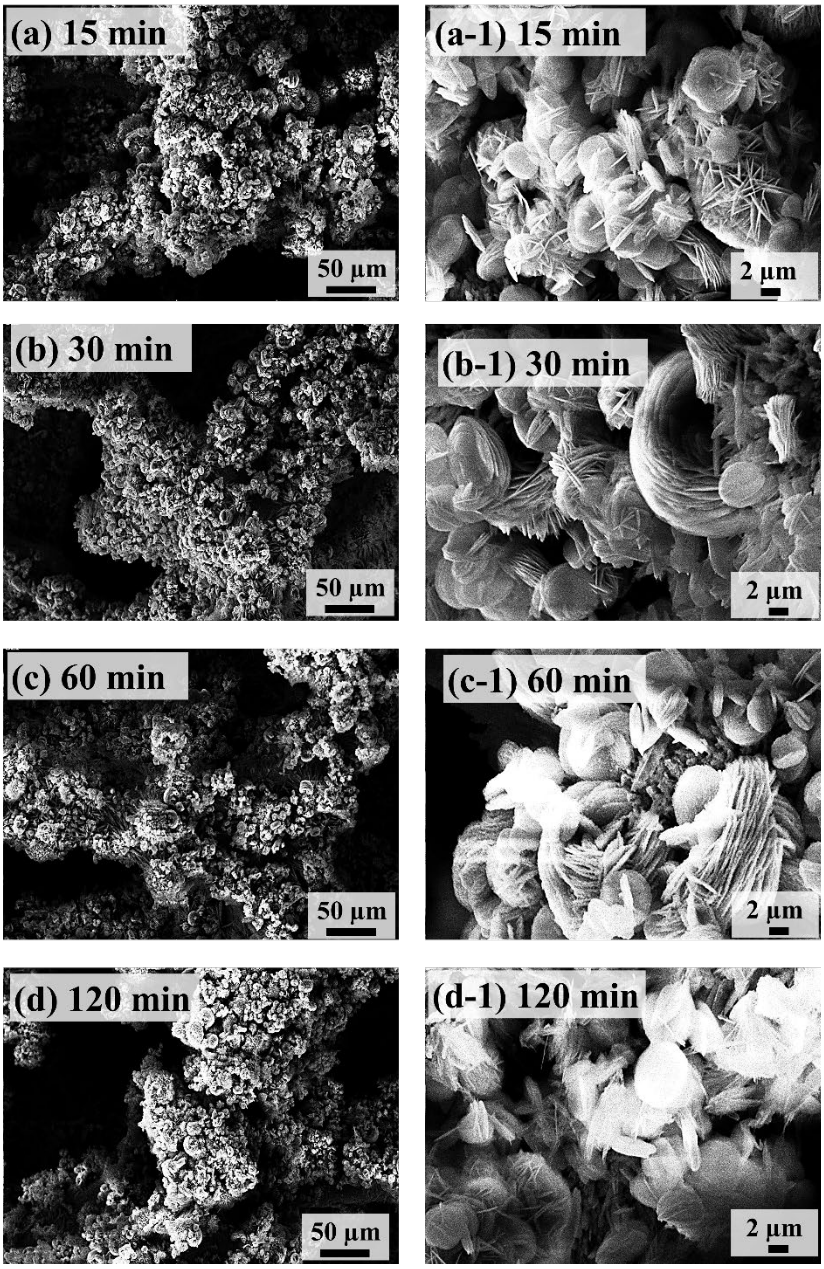

Figure S36. (**a**)–(**d**) SEM images of V-FeBP electrode fabricated with post annealing duration variation at 100 °C annealing temperature. (**a-1**)–(**d-1**) Enlarged SEM images for the corresponding electrodes.

Post-Annealing (2nd): Duration Variation (V-doped FeBP)

(a) 15 min

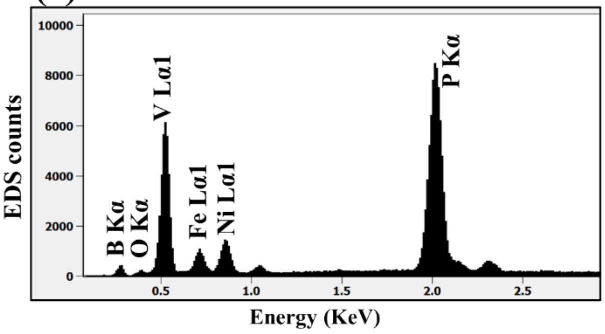

Elements	Wt %	At %
**B**	3.52	10.79
**P**	65.6	70.18
**Fe**	18.52	10.99
**V**	12.36	8.04

(b) 30 min

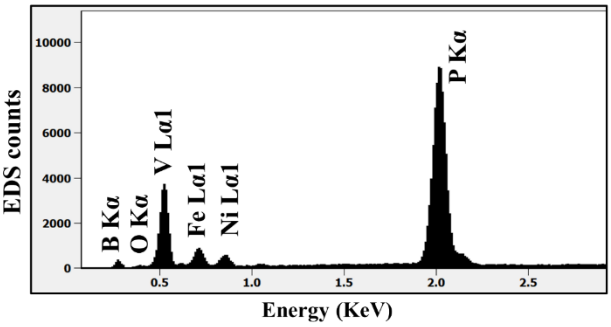

Elements	Wt %	At %
**B**	3.21	9.84
**P**	67.6	72.30
**Fe**	19.54	11.59
**V**	9.64	6.27

(c) 60 min

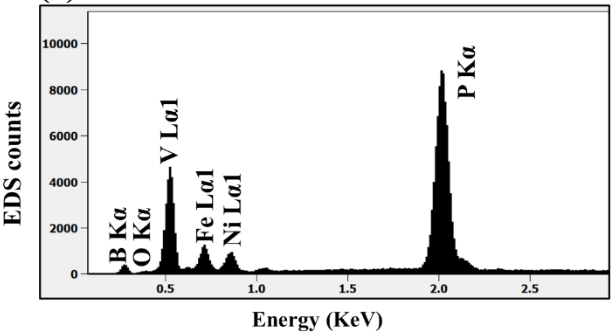

Elements	Wt %	At %
**B**	2.85	8.78
**P**	68.4	73.59
**Fe**	20.21	12.06
**V**	8.51	5.57

(d) 120 min

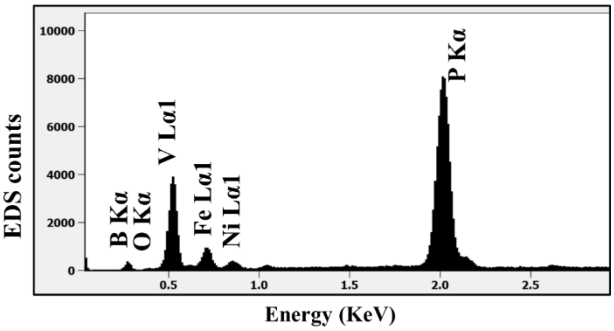

Elements	Wt %	At %
**B**	2.15	6.72
**P**	69.2	75.55
**Fe**	21.12	12.79
**V**	7.44	4.94

Figure S37. (**a**)–(**d**) EDS spectra and corresponding atomic percentage tables of the post annealing duration variation set.

Post-Annealing (2nd): Duration Variation (V-Doped FeBP)

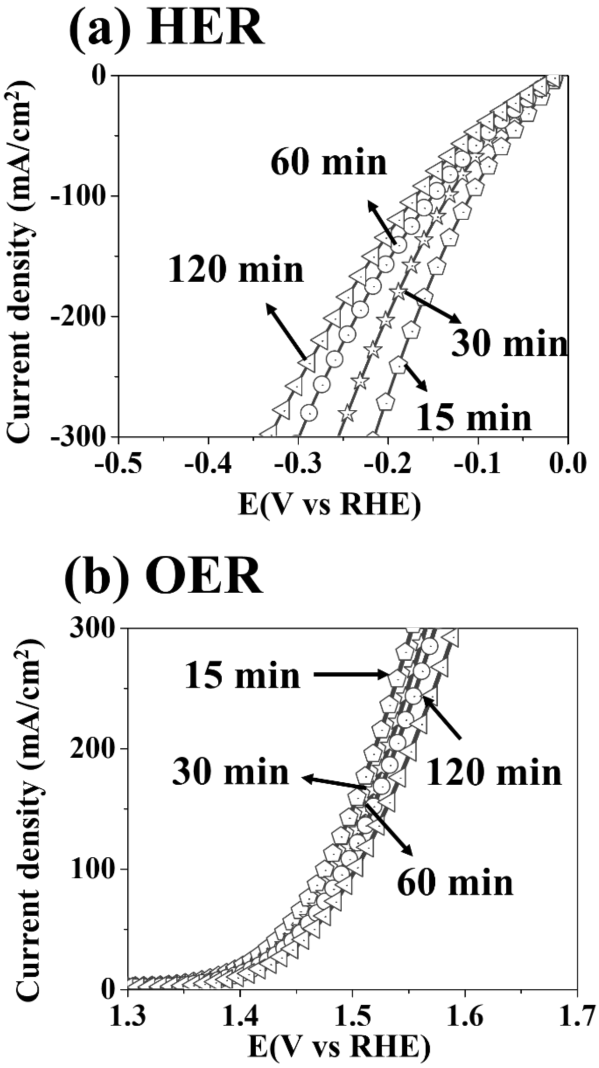

Figure S38. (**a**) and (**b**) Polarization curves of HER and OER for the V-FeBP electrodes of the post annealing duration variations set. The 15-min sample demonstrated the best HER and OER performances.

Post Annealing (2nd): Temperature Variation (V-Doped FeBP)

(a) 50 °C

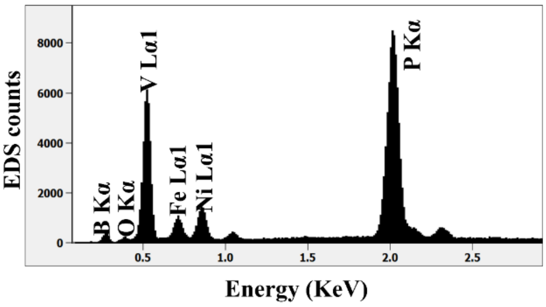

Elements	Wt %	At %
**B**	3.96	11.92
**P**	67.35	70.74
**Fe**	17.45	10.17
**V**	11.24	7.18

(b) 100 °C

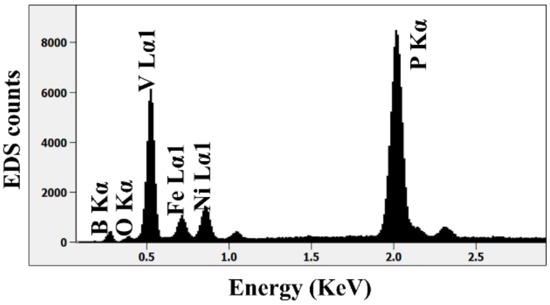

Elements	Wt %	At %
**B**	3.12	9.56
**P**	68.06	72.76
**Fe**	18.35	10.88
**V**	10.47	6.81

(c) 150 °C

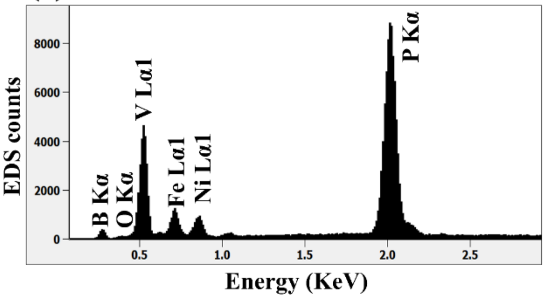

Elements	Wt %	At %
**B**	2.92	9.01
**P**	67.71	72.89
**Fe**	19.54	11.67
**V**	9.83	6.43

(d) 200 °C

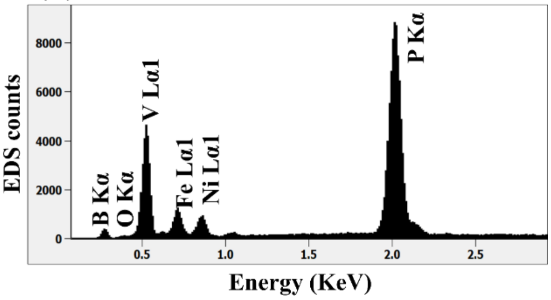

Elements	Wt %	At %
**B**	2.24	7.05
**P**	66.95	73.60
**Fe**	21.22	12.94
**V**	9.59	6.41

Figure S39. (**a**)–(**d**) EDS spectra and corresponding atomic percentage tables of the post annealing temperature variation set followed by vanadium (V) doping (V-FeBP).

Post Annealing (2nd): Temperature Variation (V-Doped FeBP)

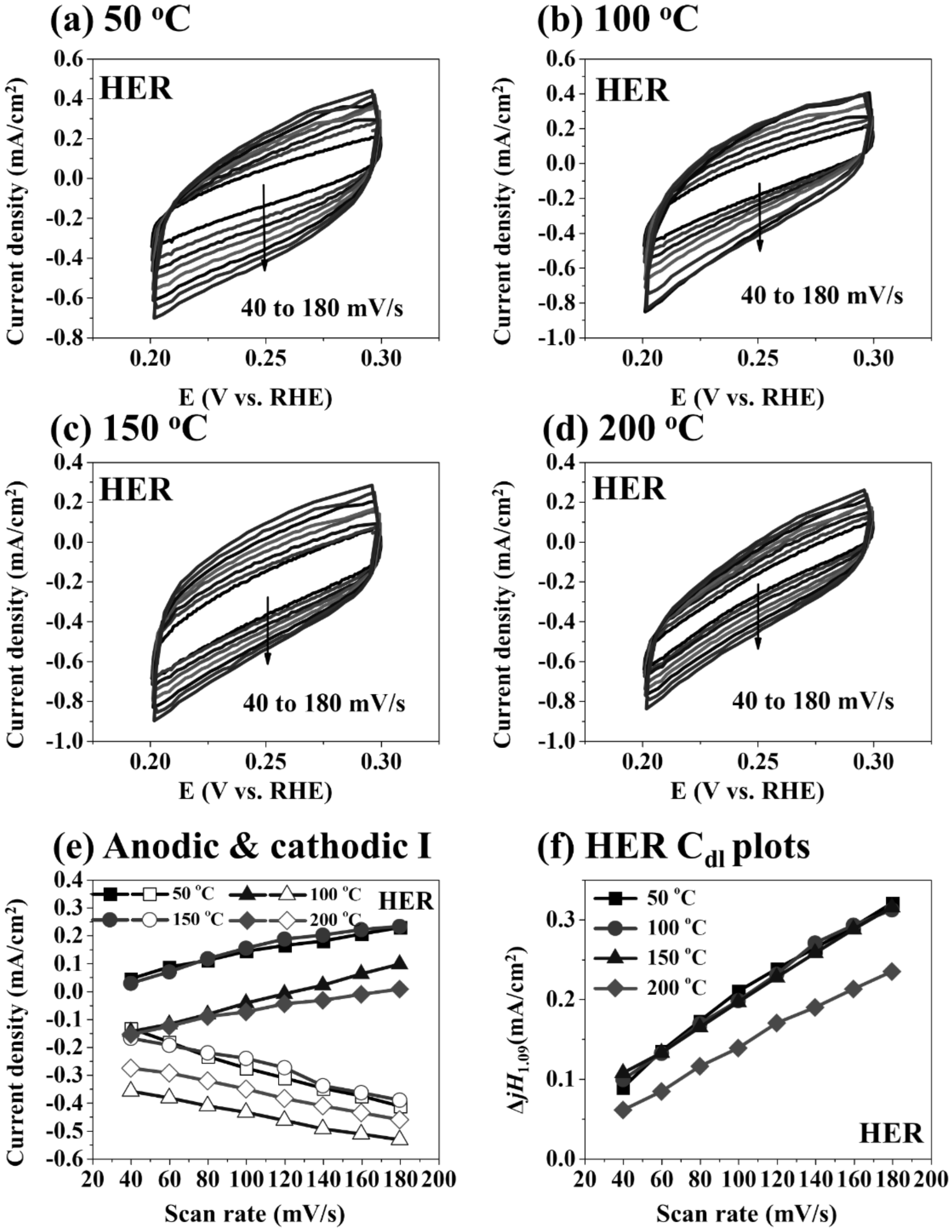

Figure S40. (**a**)–(**d**) HER CV plots of post annealing temperature variation set (V-FeBP). (**e**) Linear plot for the anodic and cathodic current density Vs scan rate. (**f**) HER double layer capacitance (C_{dl}) plots.

Post Annealing (2nd): Temperature Variation (V-Doped FeBP)

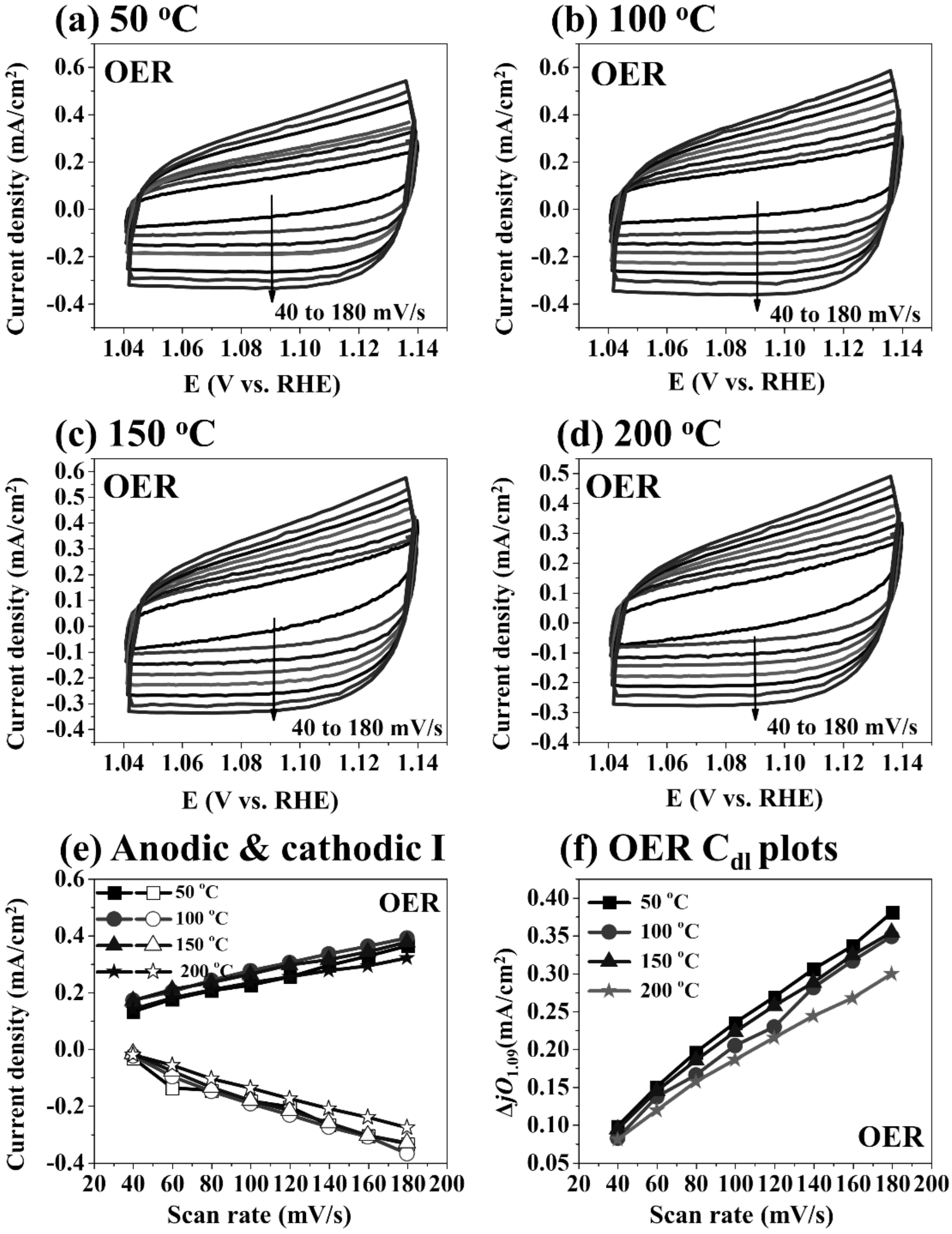

Figure S41. (**a**)–(**d**) OER CV plots of post annealing temperature vernation set (V-FeBP). (**e**) Linear plot for the anodic and cathodic current density Vs scan rate. (**f**) OER C_{dl} plots.

HER Reference: Pt/C Electrode

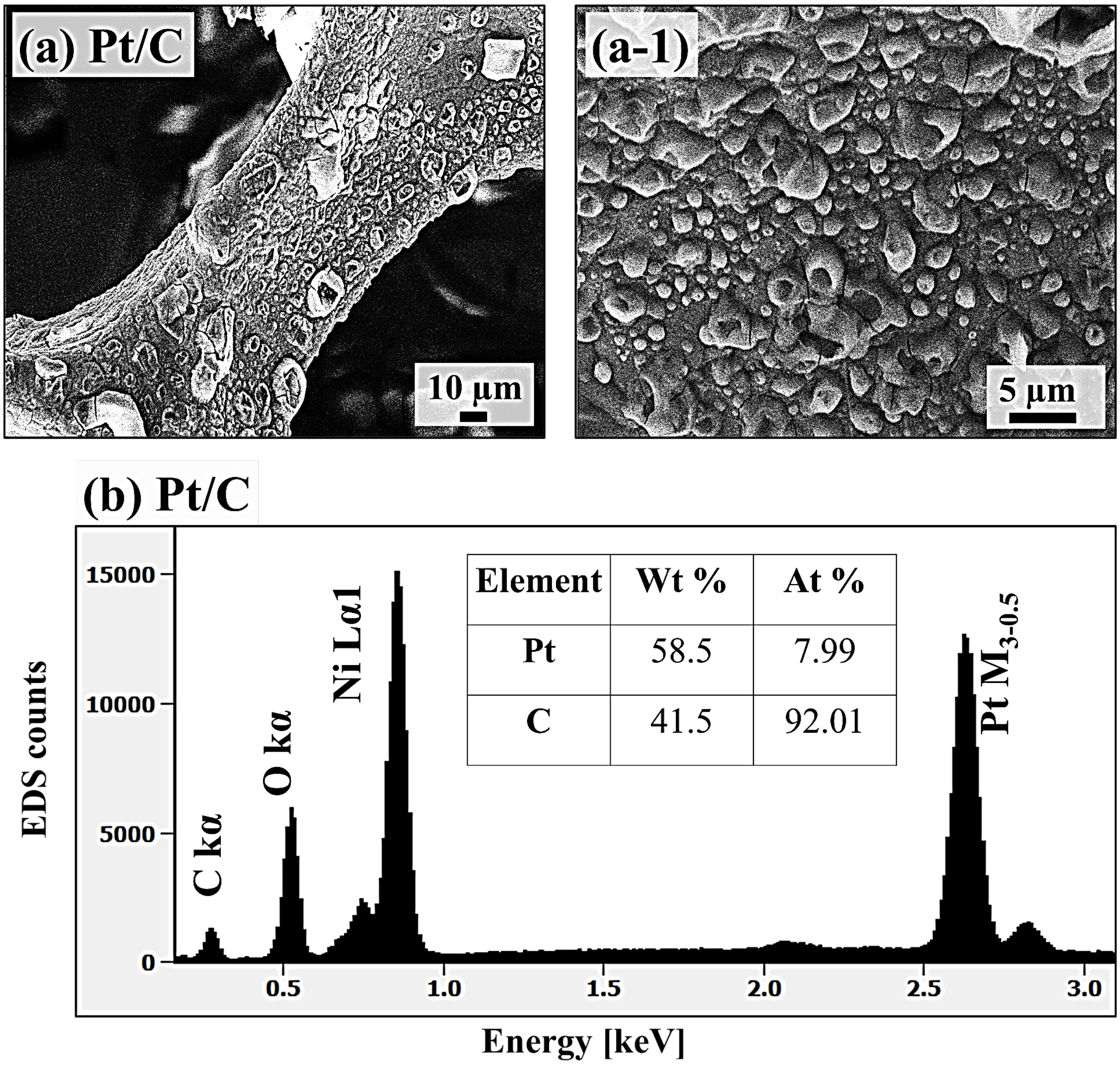

Figure S42. (**a**)–(**a-1**) SEM images of Pt/C electrode. (**b**) EDS spectra with the atomic and weight percentage. For the Pt/C electrode fabrication, 20 mg of Pt/C and 60 µL of 5% Nafion (117 solutions, Sigma-Aldrich, St. Louis, MO, USA) were dispersed into an ethanol and DI water mixture solution (2 ml, 50:50 solution) [6]. The mixture solution was then ultrasonicated for 30 min for uniform dispersion. The bare Ni foam was then immersed in the solution for 30 min. The Pt/C electrode was dried at ambient.

OER Reference: RuO_2 Electrode

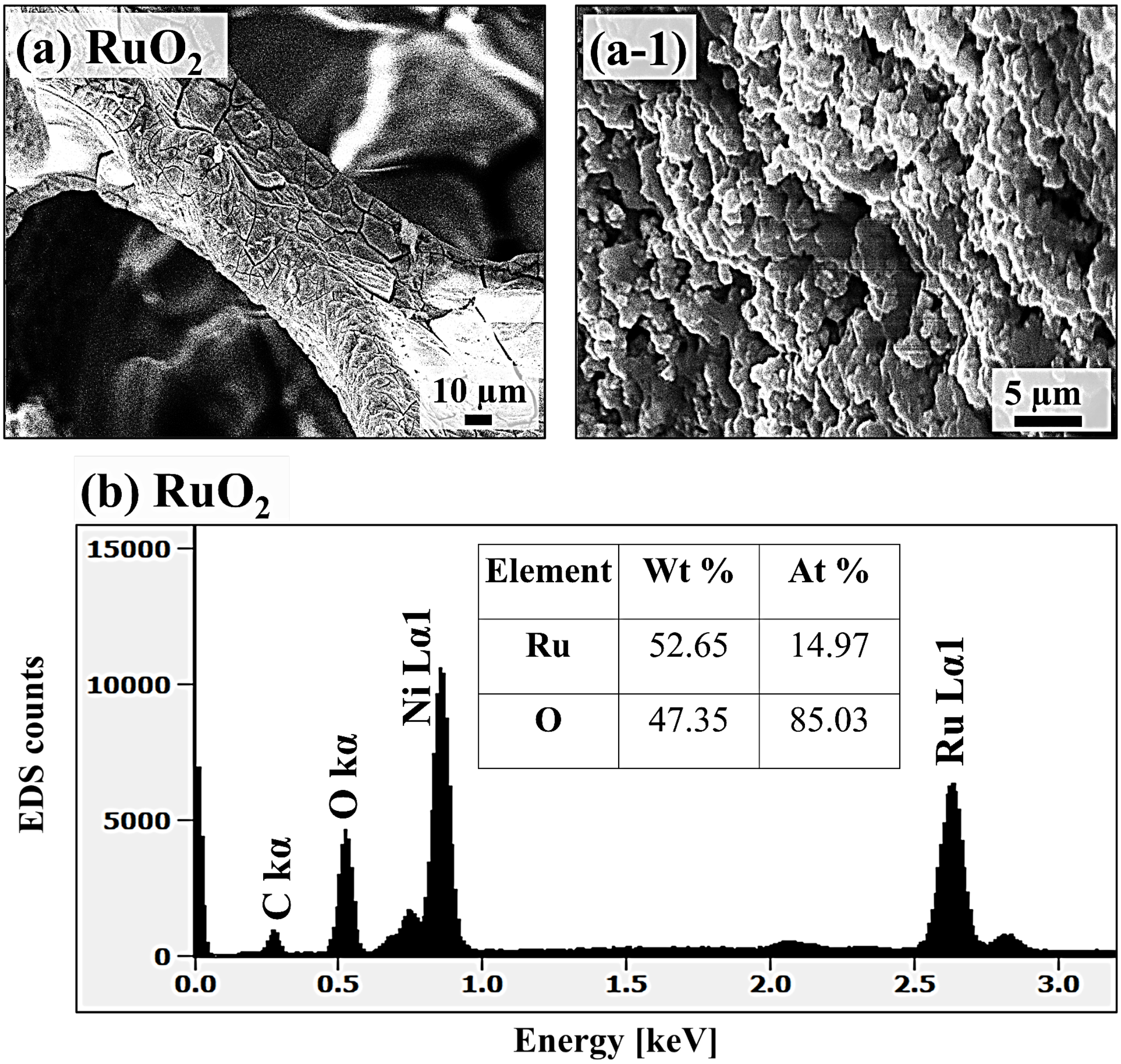

Figure S43. (**a**)–(**a-1**) Morphological analysis of RuO_2 electrode. (**b**) EDS spectra and atomic and weight percentage. To fabricate the RuO_2 electrode for the OER reference, 40 mg of RuO_2 and 60 µL of 5% Nafion (117 solutions, Sigma-Aldrich, St. Louis, MO, USA) were dispersed into the 2 ml mixture of ethanol and DI water at the volume ratio 50:50 [7]. The mixture was ultrasonically mixed for 30 min. The Ni foam was then dipped in the dispersion solution for 30 min followed by being dried in the air.

3-E Steady-State HER Current Comparison of Best Electrode

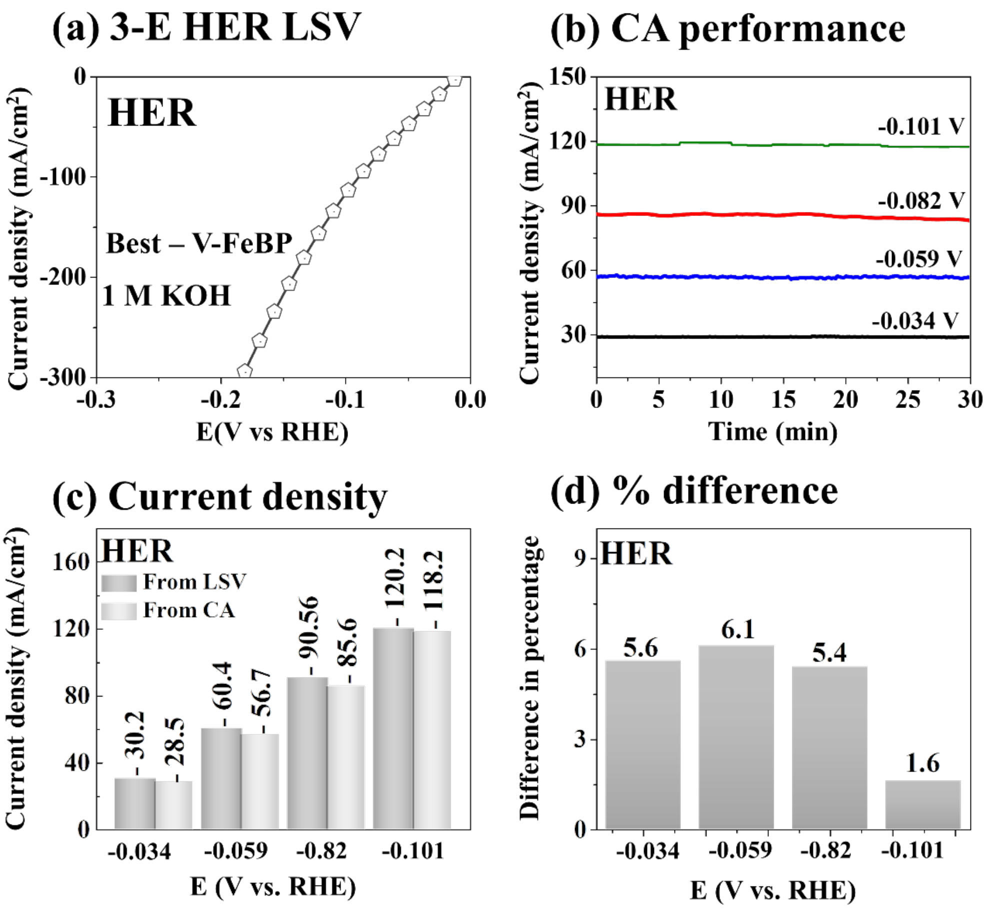

Figure S44. HER LSV and CA current comparison of the best electrode V-FeBP (50 °C) in 1 M KOH. (**a**) HER polarization curves. (**b**) CA response at −0.034, −0.059, −0.082 and −0.101 V. (**c**) Bar plot comparison of current density for LSV and CA. (**d**) Percentage difference.

3-E Steady-State OER Current Comparison of Best Electrode

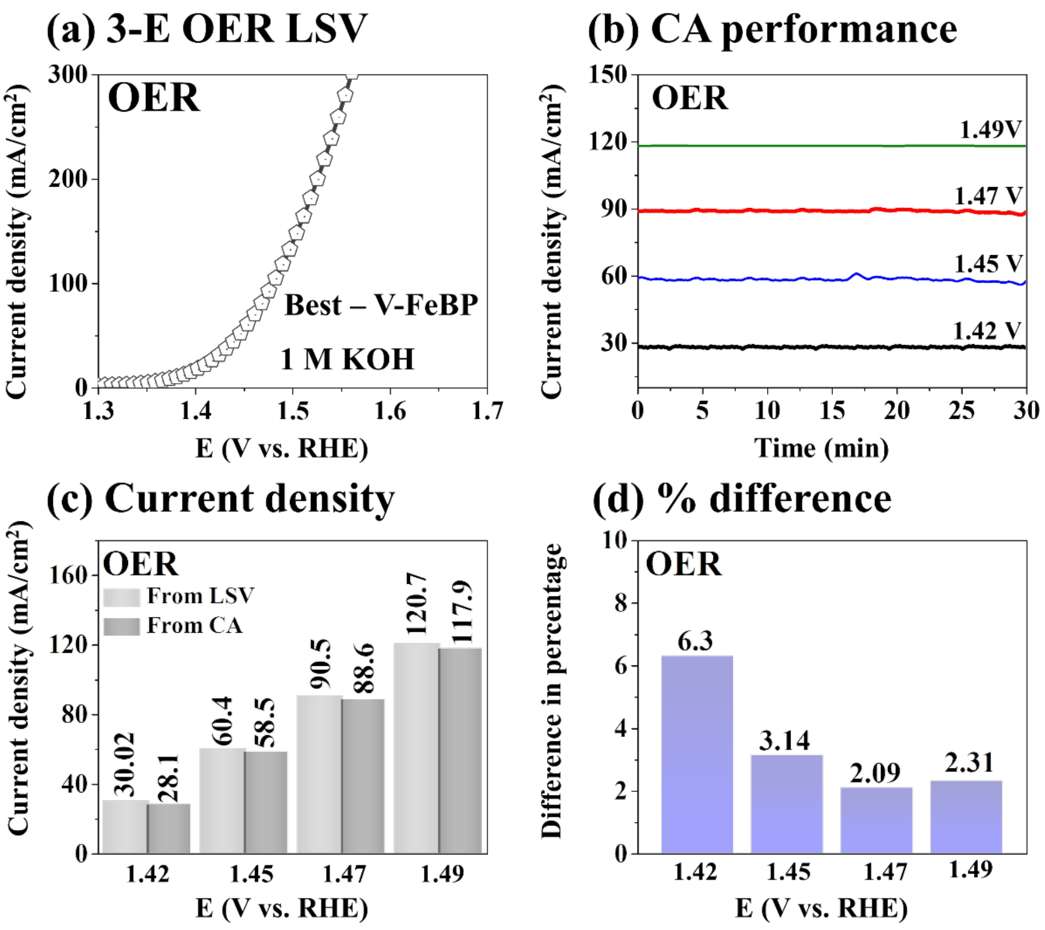

Figure S45. OER LSV and CA current comparison of the best electrode V-FeBP (50 °C) in 1 M KOH. (**b**) CA response at 1.42, 1.45, 1.47 and 1.49 V. (**c**) Bar plot comparison of current density for LSV and CA. (**d**) Percentage difference.

Post Annealing (2nd): Temperature Variation (V-Doped FeBP)

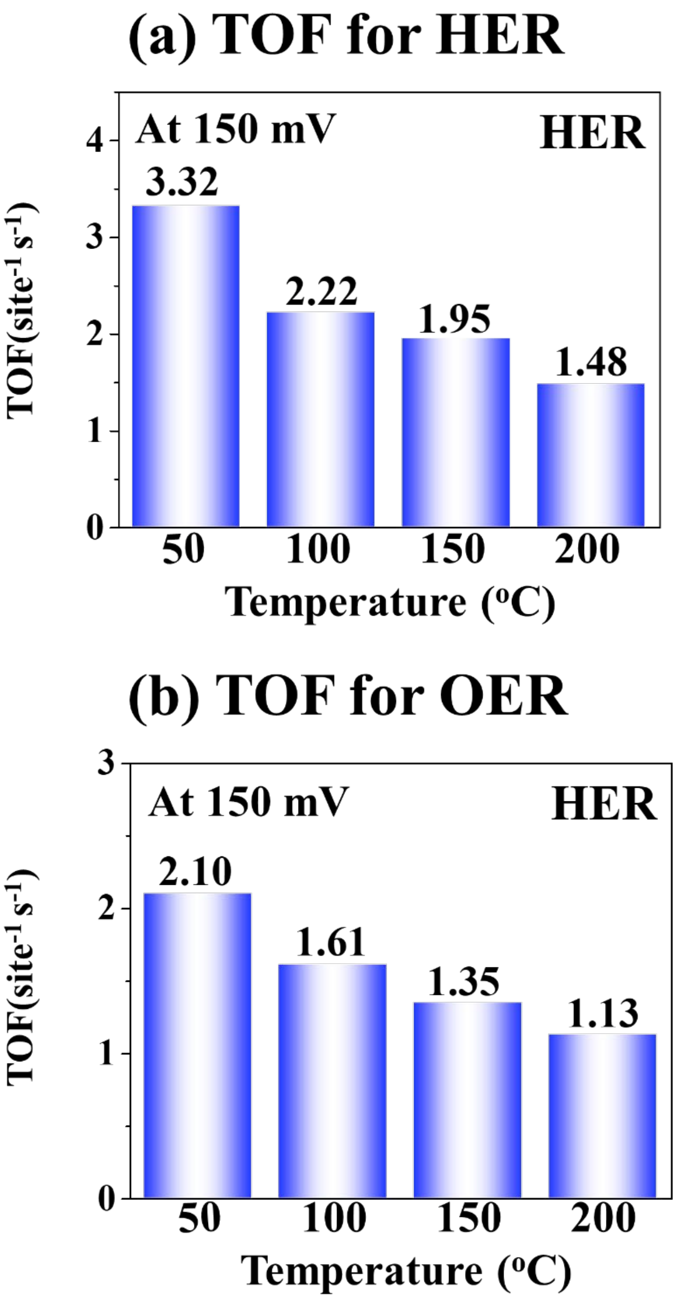

Figure S46. HER and OER turnover frequency (TOF) of post annealing temperature variation set (V-FeBP). (**a**)–(**b**) Vanadium active sites at 150 mV/cm^2. (**c**)–(**d**) Fe active sites at 150 mV/cm^2.

Turnover Frequency (TOF) Calculations for V-FeBP

TOF is defined as the number of hydrogen and oxygen created per atomic site per unit time and is used to estimate the intrinsic activity of each catalytic atomic active site under the specified reaction condition. There are various approaches used for the calculation of TOF, i.e., the redox peak integration and surface concentration and active sites [8]. A current density and number of active sites from the metallic atomic sites in the electrode at a constant overpotential can also be used to derive the intrinsic parameter of TOF [9]. The TOF can be obtained based on the following equation.

$$\mathrm{TOF} = \frac{\frac{\text{Total number of H}^2\text{ or O}^2}{\text{Geometric area (cm)}^2} \times \text{Current density}}{\text{Number of active sites}} \quad (1)$$

Total number of H_2 and O_2 at the turnover

To calculate the TOF, first, the total number of H_2 at the turnover can be calculated by the following equation (2):

$$= \left(\mathrm{j}\,\frac{\mathrm{mA}}{\mathrm{cm}^2}\right)\left(\frac{1\frac{\mathrm{C}}{\mathrm{s}}}{1000\ \mathrm{mA}}\right)\left(\frac{1\frac{\mathrm{mol}}{\mathrm{e}}}{96485\ \mathrm{C}}\right)\left(\frac{1\ \mathrm{mol\ H_2}}{2\frac{\mathrm{mol}}{\mathrm{e}}}\right)\left(\frac{6.022\times10^{23}\mathrm{mol\ H_2}}{1\ \mathrm{mol\ H_2}}\right)$$

$$= 3.12 \times 10^{15}\left(\frac{\mathrm{H_2}/\mathrm{s}}{\mathrm{cm}^2}\right)\ \mathrm{per}\ \left(\frac{\mathrm{mA}}{\mathrm{cm}^2}\right) \quad (2)$$

Total number of O_2 at the turnover can be calculated by the following equation (3):

$$= \left(\mathrm{j}\,\frac{\mathrm{mA}}{\mathrm{cm}^2}\right)\left(\frac{1\frac{\mathrm{C}}{\mathrm{s}}}{1000\ \mathrm{mA}}\right)\left(\frac{1\frac{\mathrm{mol}}{\mathrm{e}}}{96485\ \mathrm{C}}\right)\left(\frac{1\ \mathrm{mol\ O_2}}{4\frac{\mathrm{mol}}{\mathrm{e}}}\right)\left(\frac{6.022\times10^{23}\mathrm{mol\ O_2}}{1\ \mathrm{mol\ O_2}}\right)$$

$$= 1.56 \times 10^{15}\left(\frac{\mathrm{O_2}/\mathrm{s}}{\mathrm{cm}^2}\right)\ \mathrm{per}\ \left(\frac{\mathrm{mA}}{\mathrm{cm}^2}\right) \quad (3)$$

Active Site Calculations

Metallic active sites of Ru/CuBP

$$= \frac{\text{EDS }\%}{100} \times \text{amonut of loading per area} \times \textit{Molecular mass} \times \left(\frac{\text{Avogadro Number}}{\text{Per concentration (mmol)}}\right)$$

$$= \frac{10.28}{100} \times 0.31\frac{\mathrm{mg}}{\mathrm{cm}^2} \times \frac{1\,mmol}{50.94\,mg} \times \frac{19.14}{100} \times 0.31\frac{\mathrm{mg}}{\mathrm{cm}^2} \times \frac{1\,mmol}{55.8\,mg} \times \left(\frac{6.022\times10^{23}\ \mathrm{mmol}}{2\ \mathrm{mmol}}\right)$$

$$= 2.00 \times 10^{17}\ \textit{Active sites}.\,cm^{-2}\ \text{per mole}$$

The above equation can be used to determine the number of active sites. The active sites of best 200 °C V-FeBP sample can be calculated as follows. The percentage of V and Fe are 10.28 and 19.14 (wt%) from the EDS analysis. It was considered that the 1 mmol V and Fe atoms are electrochemically active in the V-FeBP catalytic surface area. The loading material of 0.31 mg for V-FeBP electrode is attained by weighing bare Ni foam (0.0430 g) and after all the growth process of V-FeBP materials (0.0433 g). The molar mass of V is 50.94 mg/mmol, where 1 amu is 1 g/mol. The molar mass of Fe is 55.8 mg/mmol.

HER TOF of V-FeBP

$$\text{HER TOF} = \frac{3.12\times10^{15}\times 95.07}{2.00\times10^{17}} = 1.483\ \mathrm{site}^{-1}\ \mathrm{s}^{-1}$$

The HER TOF of 200 °C V-FeBP electrode can be calculated as above. The 50, 100, 150 and 200 °C electrodes demonstrated the HER current density 213.2, 142.4, 125.5 and 95.07 respectively at 150 mV.

OER TOF of V-FeBP

$$\text{OER TOF} = \frac{1.56 \times 10^{15} \times 145.4}{2.00 \times 10^{17}} = 1.134 \text{ site}^{-1} \text{ s}^{-1}$$

Similarly, for OER TOF of 200 °C V-FeBP can be calculated as above. The 50, 100, 150, and 200 °C V-FeBP electrodes demonstrated the OER current density of 270, 207, 173.5 and 145.4 at 150 mV.

2-E Steady-State Current Comparison of Best Electrode

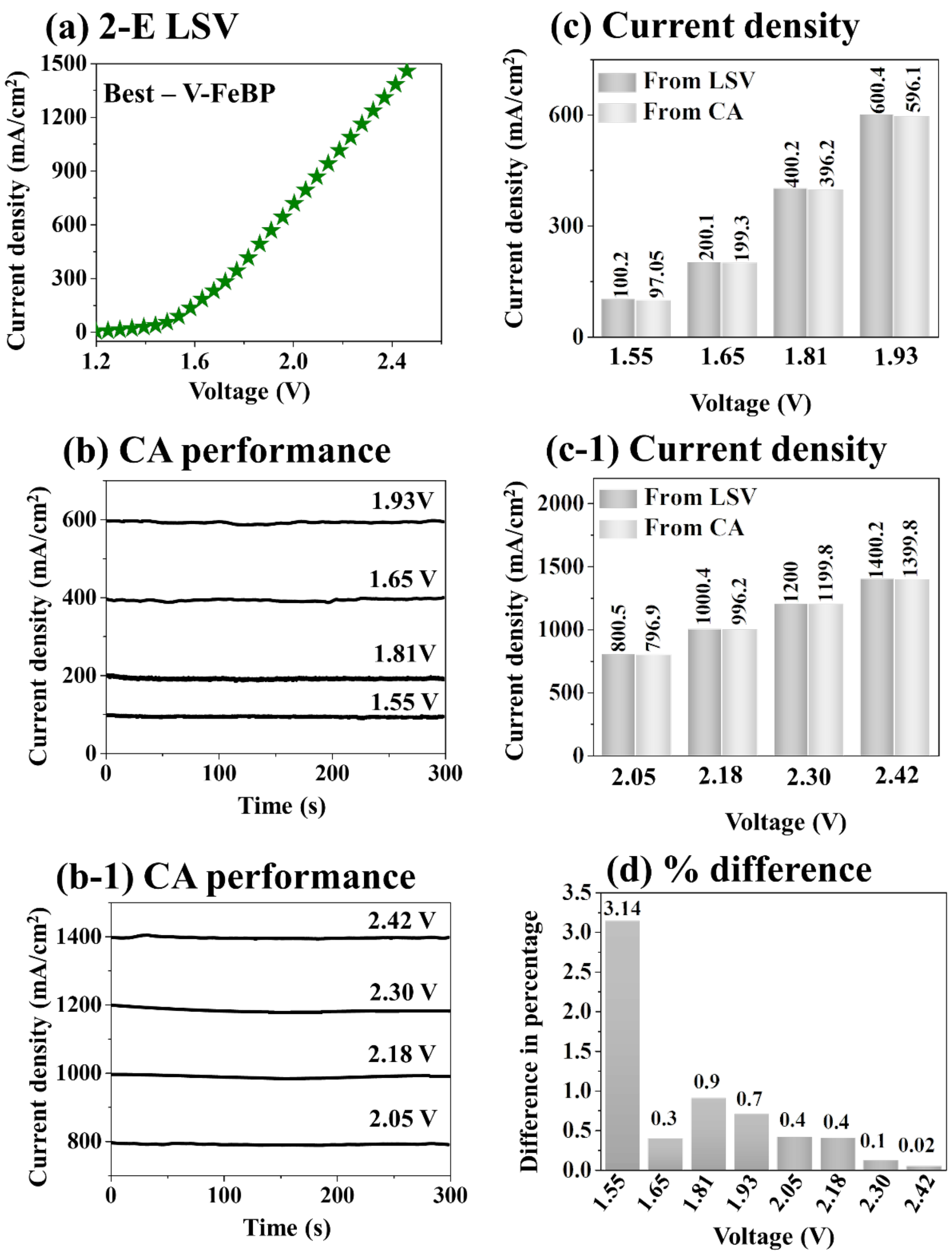

Figure S47. 2-E LSV and CA comparison of the V-FeBP ‖ V-FeBP in 1 M KOH. (**a**) HER polarization curves. (**b**)–(**c-1**) CA responses at 1.55, 1.65, 1.81, 1.93, 2.05, 2.18, 2.30, and 2.42 V. (**c**)–(**c-1**) Bar plots comparison of current density for the LSV and CA. (**d**) Percentage difference.

Comparison with State-of-Art Fe-Based Electrodes

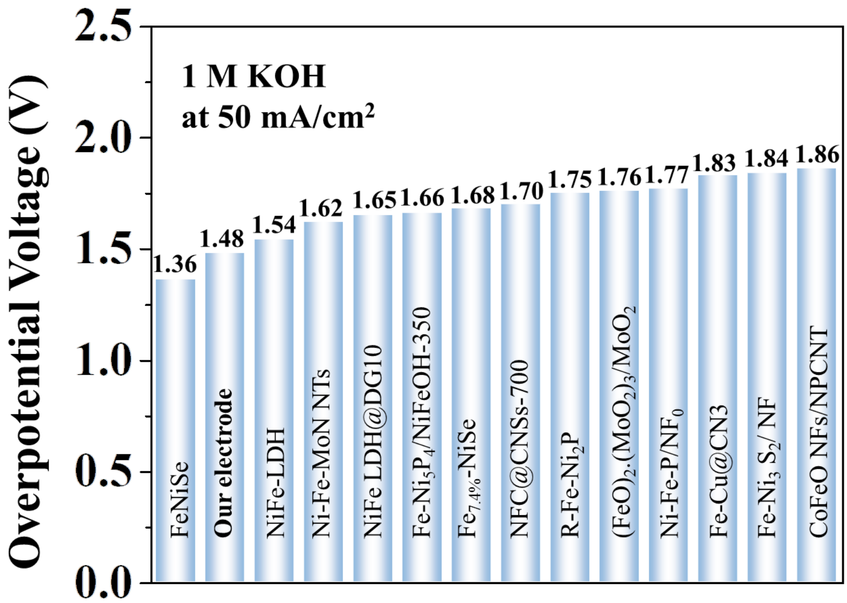

Figure S48. Comparison of 2-electrode performance with the state-of-art Fe-based electrodes at the current density of 50 mA/cm² in 1 M KOH. Related to Table 1 in the main text.

Comparison with State-of-Art Electrodes

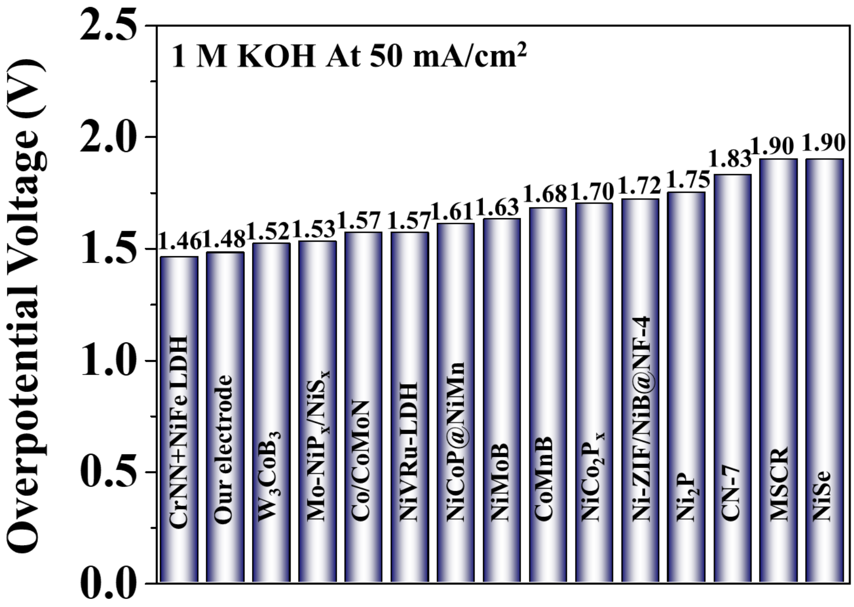

Figure S49. Comparison of 2-electrode performance with the all state-of-art electrodes at the current density of 50 mA/cm² in 1 M KOH. Related to Table S1.

3-E Comparison with State-of-Art Electrodes

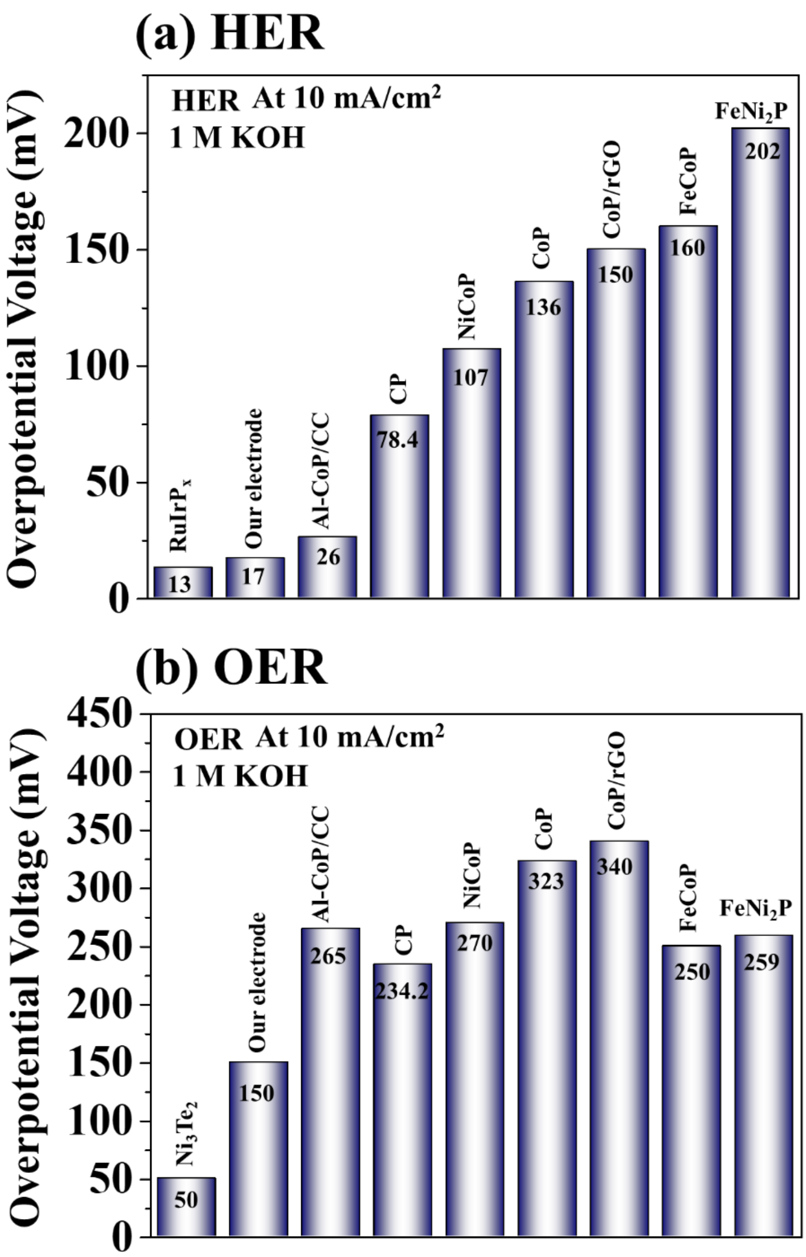

Figure S50. Comparison of 3-electrode performance with the all state-of-art electrodes at the current density of 10 mA/cm^2 in 1 M KOH. (**a**) HER. (**b**) OER Related to Table S2.

Table S1. Comparison of 2-electrode performance with the state-of-art transition metal-based electrodes at density of 50 mA/cm^2 in 1 M KOH.

Electrocatalysts	Electrolyte Solution	Overpotential [V] at 50 mA/cm^2	Year	Reference
CrNN+NiFe LDH	1 M KOH	1.46	2015	[10]
V/FeBP	1 M KOH	1.48	-	(This work)
W_3CoB_3	1 M KOH	1.52	2022	[11]
Mo-NiP_x/NiS_x	1 M KOH	1.53	2021	[12]
Co/CoMoN	1 M KOH	1.57	2022	[13]
NiVRu-LDH	1 M KOH	1.57	2019	[14]
NiCoP@NiMn	1 M KOH	1.61	2019	[15]
NiMoB	1 M KOH	1.63	2022	[16]
CoMnB	1 M KOH	1.68	2022	[17]
$NiCo_2P_x$	1 M KOH	1.70	2017	[18]
Ni-ZIF/NiB@NF-4	1 M KOH	1.72	2019	[19]
Ni_2P	1 M KOH	1.75	2016	[20]
CN-7	1 M KOH	1.83	2021	[21]
MSCR	1 M KOH	1.90	2021	[22]
NiSe	1 M KOH	1.90	2015	[23]

Table S2. Comparison of 3-electrode performance with the state-of-art transition metal-based electrodes at density of 10 mA/cm^2 in 1 M KOH.

Electrocatalysts	Electrolyte Solution	HER Overpotential [mV] at 10 mA/cm^2	OER Overpotential [mV] at 10 mA/cm^2	Year	Reference
$RuIrP_x$	1 M KOH	12	-	2019	[14]
Ni_3Te_2	1 M KOH	-	50	2018	[24]
V/FeBP	1 M KOH	17	150	-	This work
Al-CoP/CC	1 M KOH	26	265	2017	[25]
CP	1 M KOH	78.2	234.2	2020	[26]
NiCoP	1 M KOH	107	270	2018	[27]
CoP	1 M KOH	136	323	2019	[28]
CoP/rGO	1 M KOH	150	340	2016	[29]
FeCoP	1 M KOH	160	250	2018	[30]
$FeNi_2P$	1 M KOH	202	259	2021	[31]

References

1. Anantharaj, S.; Noda, S. Appropriate Use of Electrochemical Impedance Spectroscopy in Water Splitting Electrocatalysis. *ChemElectroChem* **2020**, *7*, 2297–2308. https://doi.org/10.1002/celc.202000515.
2. Yu, F.; Zhou, H.; Huang, Y.; Sun, J.; Qin, F.; Bao, J.; Goddard, W.A.; Chen, S.; Ren, Z. High-Performance Bifunctional Porous Non-Noble Metal Phosphide Catalyst for Overall Water Splitting. *Nat. Commun.* **2018**, *9*, 1–9. https://doi.org/10.1038/s41467-018-04746-z.
3. Chen, C.; Tuo, Y.; Lu, Q.; Lu, H.; Zhang, S.; Zhou, Y.; Zhang, J.; Liu, Z.; Kang, Z.; Feng, X.; et al. Hierarchical Trimetallic Co-Ni-Fe Oxides Derived from Core-Shell Structured Metal-Organic Frameworks for Highly Efficient Oxygen Evolution Reaction. *Appl. Catal. B Environ.* **2021**, *287*, 119953. https://doi.org/10.1016/j.apcatb.2021.119953.
4. Yu, L.; Zhou, H.; Sun, J.; Qin, F.; Yu, F.; Bao, J.; Yu, Y.; Chen, S.; Ren, Z. Cu Nanowires Shelled with NiFe Layered Double Hydroxide Nanosheets as Bifunctional Electrocatalysts for Overall Water Splitting. *Energy Environ. Sci.* **2017**, *10*, 1820–1827. https://doi.org/10.1039/c7ee01571b.
5. Jiang, Y.; Lu, Y. Designing Transition-Metal-Boride-Based Electrocatalysts for Applications in Electrochemical Water Splitting. *Nanoscale* **2020**, *12*, 9327–9351. https://doi.org/10.1039/d0nr01279c.
6. Palma, V.; Vaiano, V.; Matarangolo, M.; Anello, G. Comparison of Pt/C Electrocatalyst Deposition Methods for PEM Fuel Cells. *Chem. Eng. Trans.* **2018**, *70*, 1525–1530. https://doi.org/10.3303/CET1870255.
7. Yu, H.; Quan, T.; Mei, S.; Kochovski, Z.; Huang, W.; Meng, H.; Lu, Y. Prompt Electrodeposition of Ni Nanodots on Ni Foam to Construct a High-Performance Water-Splitting Electrode: Efficient, Scalable, and Recyclable. *Nano-Micro Lett.* **2019**, *11*, 1–13. https://doi.org/10.1007/s40820-019-0269-x.
8. Anantharaj, S.; Karthik, P.E.; Noda, S. The Significance of Properly Reporting Turnover Frequency in Electrocatalysis Research. *Angew. Chemie - Int. Ed.* **2021**, *60*, 23051–23067. https://doi.org/10.1002/anie.202110352.

9. Han, N.; Yang, K.R.; Lu, Z.; Li, Y.; Xu, W.; Gao, T.; Cai, Z.; Zhang, Y.; Batista, V.S.; Liu, W.; et al. Nitrogen-Doped Tungsten Carbide Nanoarray as an Efficient Bifunctional Electrocatalyst for Water Splitting in Acid. *Nat. Commun.* **2018**, *9*, 1–10. https://doi.org/10.1038/s41467-018-03429-z.
10. Gong, M.; Zhou, W.; Kenney, M.J.; Kapusta, R.; Cowley, S.; Wu, Y.; Lu, B.; Lin, M.C.; Wang, D.Y.; Yang, J.; et al. Blending Cr2O3 into a NiO-Ni Electrocatalyst for Sustained Water Splitting. *Angew. Chemie - Int. Ed.* **2015**, *54*, 11989–11993. https://doi.org/10.1002/anie.201504815.
11. Habib, M.A.; Mandavkar, R.; Burse, S.; Lin, S.; Kulkarni, R.; Patil, C.S.; Jeong, J.-H.; Lee, J. Design of Boron-Based Ternary W3CoB3 Electrocatalyst for the Improved HER and OER Performances. *Mater. Today Energy* **2022**, 101021. https://doi.org/10.1016/j.mtener.2022.101021.
12. Wang, J.; Zhang, M.; Yang, G.; Song, W.; Zhong, W.; Wang, X.; Wang, M.; Sun, T.; Tang, Y. Heterogeneous Bimetallic Mo-NiPx/NiSy as a Highly Efficient Electrocatalyst for Robust Overall Water Splitting. *Adv. Funct. Mater.* **2021**, *31*, 1–8. https://doi.org/10.1002/adfm.202101532.
13. Ma, H.; Chen, Z.; Wang, Z.; Singh, C.V.; Jiang, Q. Interface Engineering of Co/CoMoN/NF Heterostructures for High-Performance Electrochemical Overall Water Splitting. *Adv. Sci.* **2022**, *2105313*, 1–9. https://doi.org/10.1002/advs.202105313.
14. Wang, D.; Li, Q.; Han, C.; Lu, Q.; Xing, Z.; Yang, X. Atomic and Electronic Modulation of Self-Supported Nickel-Vanadium Layered Double Hydroxide to Accelerate Water Splitting Kinetics. *Nat. Commun.* **2019**, *10*, 3899. https://doi.org/10.1038/s41467-019-11765-x.
15. Wang, P.; Qi, J.; Chen, X.; Li, C.; Li, W.; Wang, T.; Liang, C. Three-Dimensional Heterostructured NiCoP@NiMn-Layered Double Hydroxide Arrays Supported on Ni Foam as a Bifunctional Electrocatalyst for Overall Water Splitting. *ACS Appl. Mater. Interfaces* **2020**, *12*, 4385–4395. https://doi.org/10.1021/acsami.9b15208.
16. Mandavkar, R.; Habib, A.; Lin, S.; Kulkarni, R.; Burse, S.; Jeong, J.; Lee, J. Electron Enriched Ternary NiMoB Electrocatalyst for Improved Overall Water Splitting : Better Performance as Compared to the Pt / C || RuO 2 at High Current Density. *Appl. Mater. Today* **2022**, *29*, 101579. https://doi.org/10.1016/j.apmt.2022.101579.
17. Lin, S.; Habib, A.; Mandavkar, R.; Kulkarni, R.; Burse, S.; Chung, Y.; Liu, C.; Wang, Z.; Lin, S.; Jeong, J.; et al. Higher Water-Splitting Performance of Boron-Based Porous CoMnB Electrocatalyst over the Benchmarks at High Current in 1 m KOH and Real Sea Water. **2022**, *2200213*, 1–14. https://doi.org/10.1002/adsu.202200213.
18. Bai, X.; Ren, Z.; Du, S.; Meng, H.; Wu, J.; Xue, Y.; Zhao, X.; Fu, H. In-Situ Structure Reconstitution of NiCo2Px for Enhanced Electrochemical Water Oxidation. *Sci. Bull.* **2017**, *62*, 1510–1518. https://doi.org/10.1016/j.scib.2017.10.019.
19. Xu, H.; Fei, B.; Cai, G.; Ha, Y.; Liu, J.; Jia, H.; Zhang, J.; Liu, M.; Wu, R. Boronization-Induced Ultrathin 2D Nanosheets with Abundant Crystalline–Amorphous Phase Boundary Supported on Nickel Foam toward Efficient Water Splitting. *Adv. Energy Mater.* **2020**, *10*, 1–8. https://doi.org/10.1002/aenm.201902714.
20. Menezes, P.W.; Indra, A.; Das, C.; Walter, C.; Göbel, C.; Gutkin, V.; Schmeißer, D.; Driess, M. Uncovering the Nature of Active Species of Nickel Phosphide Catalysts in High-Performance Electrochemical Overall Water Splitting. *ACS Catal.* **2017**, *7*, 103–109. https://doi.org/10.1021/acscatal.6b02666.
21. Niu, J.; Yue, Y.; Yang, C.; Wang, Y.; Qin, J.; Zhang, X.; Wu, Z.S. Ultrarapid Synthesis Ni-Cu Bifunctional Electrocatalyst by Self-Etching Electrodeposition for High-Performance Water Splitting Reaction. *Appl. Surf. Sci.* **2021**, *561*, 150030. https://doi.org/10.1016/j.apsusc.2021.150030.
22. Maiti, A.; Srivastava, S.K. Ru-Doped CuO/MoS2Nanostructures as Bifunctional Water-Splitting Electrocatalysts in Alkaline Media. *ACS Appl. Nano Mater.* **2021**, *4*, 7675–7685. https://doi.org/10.1021/acsanm.1c00791.
23. Tang, C.; Cheng, N.; Pu, Z.; Xing, W.; Sun, X. NiSe Nanowire Film Supported on Nickel Foam: An Efficient and Stable 3D Bifunctional Electrode for Full Water Splitting. *Angew. Chemie - Int. Ed.* **2015**, *54*, 9351–9355. https://doi.org/10.1002/anie.201503407.
24. De Silva, U.; Masud, J.; Zhang, N.; Hong, Y.; Liyanage, W.P.R.; Asle Zaeem, M.; Nath, M. Nickel Telluride as a Bifunctional Electrocatalyst for Efficient Water Splitting in Alkaline Medium. *J. Mater. Chem. A* **2018**, *6*, 7608–7622. https://doi.org/10.1039/C8TA01760C.
25. Zhang, R.; Tang, C.; Kong, R.; Du, G.; Asiri, A.M.; Chen, L.; Sun, X. Al-Doped CoP Nanoarray: A Durable Water-Splitting Electrocatalyst with Superhigh Activity. *Nanoscale* **2017**, *9*, 4793–4800. https://doi.org/10.1039/C7NR00740J.
26. Kim, D.; Qin, X.; Yan, B.; Piao, Y. Sprout-Shaped Mo-Doped CoP with Maximized Hydrophilicity and Gas Bubble Release for High-Performance Water Splitting Catalyst. *Chem. Eng. J.* **2021**, *408*, 127331. https://doi.org/10.1016/j.cej.2020.127331.
27. Hu, E.; Feng, Y.; Nai, J.; Zhao, D.; Hu, Y.; Lou, X.W. Construction of Hierarchical Ni-Co-P Hollow Nanobricks with Oriented Nanosheets for Efficient Overall Water Splitting. *Energy Environ. Sci.* **2018**, *11*, 872–880. https://doi.org/10.1039/c8ee00076j.
28. Ji, L.; Wang, J.; Teng, X.; Meyer, T.J.; Chen, Z. CoP Nanoframes as Bifunctional Electrocatalysts for Efficient Overall Water Splitting. *ACS Catal.* **2020**, *10*, 412–419. https://doi.org/10.1021/acscatal.9b03623.
29. Jiao, L.; Zhou, Y.X.; Jiang, H.L. Metal-Organic Framework-Based CoP/Reduced Graphene Oxide: High-Performance Bifunctional Electrocatalyst for Overall Water Splitting. *Chem. Sci.* **2016**, *7*, 1690–1695. https://doi.org/10.1039/c5sc04425a.
30. Sun, K.; Wang, K.; Yu, T.; Liu, X.; Wang, G.; Jiang, L.; Bu, Y.; Xie, G. High-Performance Fe–Co–P Alloy Catalysts by Electroless Deposition for Overall Water Splitting. *Int. J. Hydrogen Energy* **2019**, *44*, 1328–1335. https://doi.org/10.1016/j.ijhydene.2018.11.182. Ayom, G.E.; Khan, M.D.; Choi, J.; Gupta, R.K.; van Zyl, W.E.; Revaprasadu, N. Synergistically Enhanced Performance of Transition-Metal Doped Ni2P for Supercapacitance and Overall Water Splitting. *Dalt. Trans.* **2021**, *50*, 11821–11833. https://doi.org/10.1039/d1dt01058a.
